# Discovery of the sEH Inhibitor Epoxykynin as a Potent Kynurenine Pathway Modulator

**DOI:** 10.1021/acs.jmedchem.3c02245

**Published:** 2024-03-12

**Authors:** Lara Dötsch, Caitlin Davies, Elisabeth Hennes, Julia Schönfeld, Adarsh Kumar, Celine Da Cruz Lopes Guita, Johanna H.M. Ehrler, Kerstin Hiesinger, Sasikala Thavam, Petra Janning, Sonja Sievers, Stefan Knapp, Ewgenij Proschak, Slava Ziegler, Herbert Waldmann

**Affiliations:** † Department of Chemical Biology, Max Planck Institute of Molecular Physiology, Otto-Hahn-Strasse 11, Dortmund 44227, Germany; ‡ Department of Chemical Biology, Technical University of Dortmund, Otto-Hahn-Strasse 6, Dortmund 44227, Germany; § Compound Management and Screening Center (COMAS), Otto-Hahn-Strasse 15, Dortmund 44227, Germany; ∥ Goethe University Frankfurt, Institute of Pharmaceutical Chemistry, Max-von-Laue-Strasse 9, Frankfurt 60438, Germany; ⊥ Structural Genomics Consortium, Buchmann Institute for Molecular Life Sciences, Goethe University Frankfurt, Max-von-Laue-Strasse 15, Frankfurt 60438, Germany

## Abstract

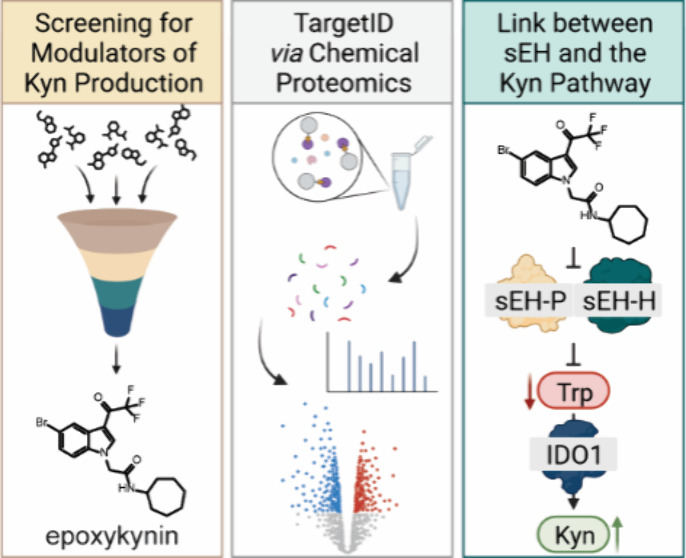

Disease-related phenotypic assays enable unbiased discovery of novel bioactive small molecules and may provide novel insights into physiological systems and unprecedented molecular modes of action (MMOA). Herein, we report the identification and characterization of epoxykynin, a potent inhibitor of the soluble epoxide hydrolase (sEH). Epoxykynin was discovered by means of a cellular assay monitoring modulation of kynurenine (Kyn) levels in BxPC-3 cells upon stimulation with the cytokine interferon-γ (IFN-γ) and subsequent target identification employing affinity-based chemical proteomics. Increased Kyn levels are associated with immune suppression in the tumor microenvironment and, thus, the Kyn pathway and its key player indoleamine 2,3-dioxygenase 1 (IDO1) are appealing targets in immuno-oncology. However, targeting IDO1 directly has led to limited success in clinical investigations, demonstrating that alternative approaches to reduce Kyn levels are in high demand. We uncover a cross-talk between sEH and the Kyn pathway that may provide new opportunities to revert cancer-induced immune tolerance.

## Introduction

Phenotypic screening allows for the identification of small molecules that modulate specific cellular processes in their natural environment, i.e., in the cell. This may lead to the discovery of novel bioactive compounds as well as better understanding of biological pathways. Compared to target-based approaches, phenotypic assays enable a less biased detection of biologically active compounds.^1^ The design of physiologically relevant screening assays should include the use of a disease-relevant system, employing a physiological stimulus and an appropriate downstream readout (phenotypic screening “rule of 3”).^2^

Indoleamine 2,3-dioxygenase 1 (IDO1) is a heme-containing enzyme that catalyzes the conversion of l-tryptophan (Trp) into the metabolite kynurenine (Kyn).^3^ IDO1 plays a critical role in the suppression of the immune system, particularly in the context of cancer.^4−6^ The reduction of Trp in the tumor microenvironment and the simultaneous production of Kyn leads to T cell dysfunction and immune tolerance.^7^ Moreover, IDO1 has additional signaling functions independent of enzymatic activity.^8^ Depending on the stimulus, either tolerance or immunity is induced via two distinct immunoreceptor tyrosine-based inhibitory motifs (ITIMs) of the small, noncatalytic domain of IDO1.^9−11^ Targeting the enzymatic activity of IDO1 has shown promising results in the treatment of cancer in preclinical studies and more than 50 different clinical trials with the most advanced IDO1 inhibitor epacadostat have been launched.^12^ However, the recent failure of a large phase III trial, testing epacadostat in combination with the anti-PD1 antibody pembrolizumab (ECHO-301/KN-252),^13^ has put many trials on hold. Reasons for failure include that the patients for the study were not specifically selected for IDO1 expression in the tumor, compensatory expression of the two other Trp-catabolizing dioxygenases tryptophan 2,3-dioxygenase (TDO) and indoleamine 2,3-dioxygenase 2 (IDO2), disregard of the noncatalytic functions of IDO1 or activation of the aryl hydrocarbon receptor (AhR) by epacadostat, leading to immune tolerance.^14−17^ The negative outcome of ECHO-301 showed that consideration of the Kyn pathway as a whole is crucial for its relevance in immuno-oncology. Therefore, alternative approaches for reduction of Kyn levels and new targets are in high demand.

We recently reported on the development of a cell-based assay monitoring Kyn levels after stimulation of BxPC-3 cells with the cytokine interferon-γ (IFN-γ) to induce expression of IDO1.^34^ Using this assay, we have now identified *N*-substituted indoles as a compound class that potently reduce cellular Kyn levels upon stimulation of cancer cells with IFN-γ.^34,35^ These small molecules do not inhibit IDO1 activity or expression, but modulate the Kyn pathway by targeting soluble epoxide hydrolase (sEH). sEH is a bifunctional enzyme bearing a C-terminal lipid epoxide hydrolase domain (sEH-H) and an N-terminal lipid phosphatase moiety (sEH-P),^18−20^ both separated by a proline-rich linker.^21^ While the biological role of sEH-P still remains elusive,^22^ sEH-H was identified as part of the CYP epoxygenase branch of the arachidonic acid (AA) cascade.^23^ It catalyzes the hydrolysis of epoxy fatty acids, such as epoxyeicosatrienoic acids (EETs), to their corresponding vicinal diols.^21^ Thus, sEH-H plays a vital role in the catabolism of bioavailable epoxides, contributing to the detoxification of xenobiotics and regulation of signaling molecules.^24,25^ sEH-H is involved in a variety of disease-associated pathways, e.g., hypertension,^26^ diabetes,^27^ inflammation,^28^ and cancer progression.^29^ Therefore, several classes of sEH inhibitors have been developed which mimic epoxides, such as ureas, carbamates, and amides.^30,31^ Additionally, the synergism of sEH and proteins assigned to the other two major pathways of the AA cascade has been exploited to design multitarget dual-inhibitors.^32,33^

The most potent derivative, termed epoxykynin, inhibits the hydrolase activity of sEH but not its phosphatase activity. Our results demonstrate a previously unrecognized dependence of the Kyn pathway on sEH function. These findings open up new avenues for the application of sEH inhibitors to modulate disease states linked to increased Kyn production.

## Results and Discussion

We employed a cell-based assay and screened 157,332 commercial and in-house synthesized compounds to identify modulators of Kyn levels in BxPC-3 cells upon stimulation with IFN-γ.^34^ In contrast to IDO1, the expression of the two other Trp-catabolizing enzymes TDO and IDO2 cannot be induced by cytokines.^36−38^ Therefore, compounds identified by this screening method most likely interfere with IDO1-mediated Kyn production.

The 1,3,5-substituted indole **1a** was identified as the initial hit compound and potently reduced cellular Kyn levels by 95.5 ± 3.0% at 7.1 μM with an IC_50_ value of 90 ± 26 nM (Table 1, Table S1). To validate the screening results, the impact of **1a** on cellular Trp and Kyn levels was quantified via LC-MS as an orthogonal assay readout (Figure S1A). Treatment with IFN-γ stimulates the expression of IDO1 in Bx-PC3 cells and, thereby, increases Kyn levels and reduces Trp levels. In the presence of IFN-γ, compound **1a** dose-dependently inhibited Kyn production (Figure S1A). At 10 μM, hardly any Kyn was detectable, and the consumption of supplemented Trp increased in a concentration-dependent manner (Figure S1A). Interestingly, compound **1a** did not inhibit the *in vitro* enzymatic activity of IDO1 (Figure S1B). In IFN-γ-stimulated HeLa cells Kyn production was reduced (Figure S1C), whereas *IDO1* expression and IDO1 protein levels were not affected (Figure S1D-S1F).

**Table 1:**
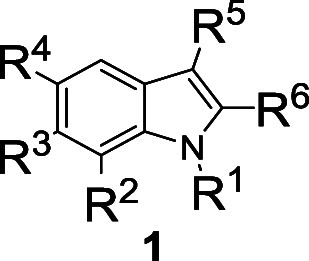

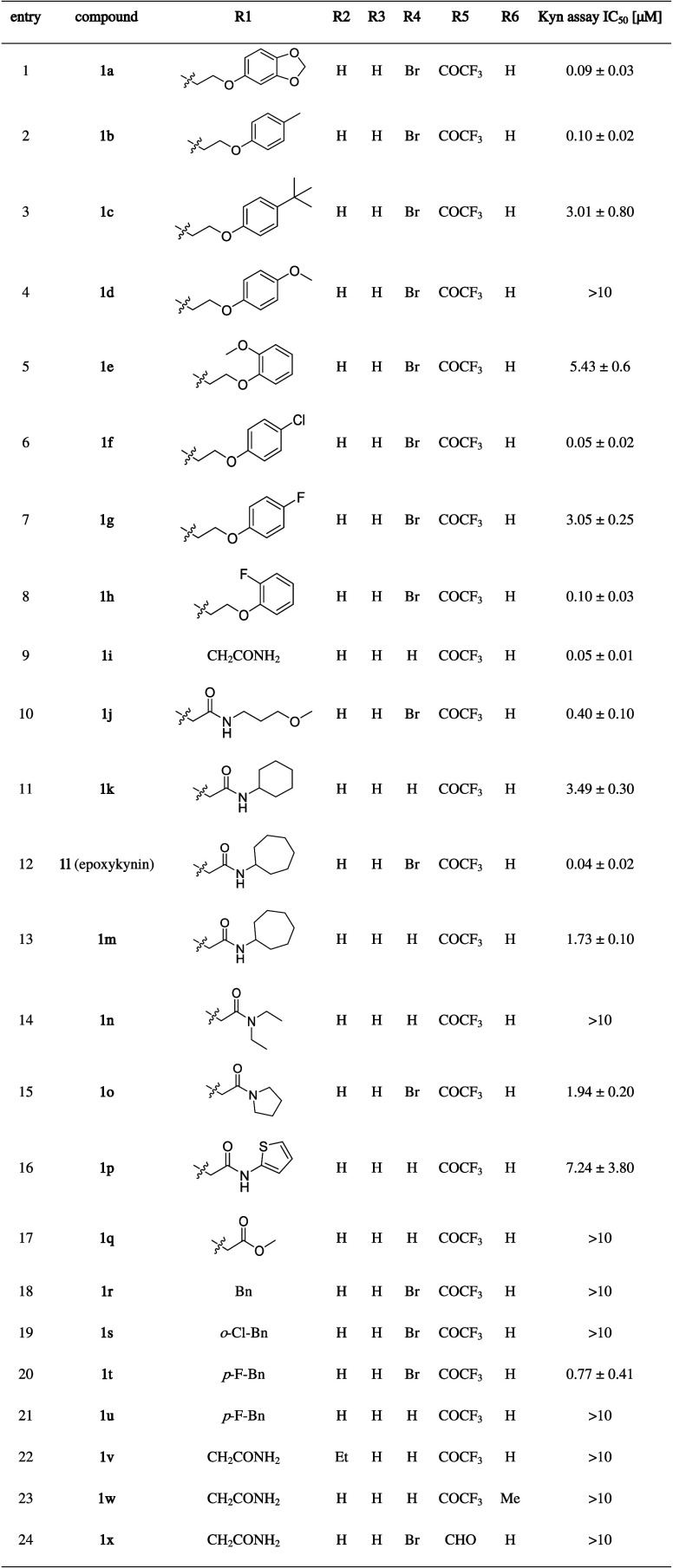
Structure–Activity Relationship (SAR) Determined for Selected Compounds for Reduction of Kyn Levels (See also Table S1)^a^

a IC_50_ values were determined in BxPC-3 cells using the automated Kyn assay. Data are mean values ± SD (*n* ≥ 3).

Investigation of the structure–activity relationship (SAR) for the indole derivative class defined by the structure of **1a** revealed that the *N*-substituent R^1^ is crucial for the biological activity (Table 1 and Table S1). Generally, amide and ether groups as *N*-alkyl substituents in the R^1^ position were well tolerated (Table 1, entries 1–16), while ester or ketone substitutions of the *N*-alkyl group decreased the activity (Table 1, entry 17 and Table S1). Comparison of aromatic ether substituents at the *N*-alkyl group revealed that small, electron-withdrawing groups on the phenyl ring were favorable (Table 1, entries 2–8). Introduction of hydrophobic and lipophilic moieties on the indole nitrogen improved the potency (Table 1, entries 11–13). Aromatic residues with a halogen in *para*-position were favorable (Table 1, entries 18–21), while most heterocycles were less active or inactive (entry 16 and Table S1). Comparison of compounds **1l** and **1m** (Table 1, entries 12 and 13) showed that substituting the R^4^ position with bromine led to a 50-fold increase in activity (see also Table 1, entries 20–21). Introducing substituents in R^2^, R^3^, and R^6^ positions generally resulted in less active compounds (Table 1, entries 22 and 23, and Table S1). Interestingly, only the trifluoroacetyl group in R^5^ position gave good IC_50_ values, which suggests that a small and strong electron-withdrawing group in this position is beneficial for biological activity (Table 1, entries 9 and 24, Table S1). Ultimately, compound **1l**, termed epoxykynin (Table 1, entry 12), which combines a hydrophobic *N*-cycloheptyl acetamide at R^1^ with bromine and trifluoroacetyl residues at R^4^ and R^5^ positions, was identified as the most potent derivative with an IC_50_ value of 36 ± 15 nM.

**Figure 1:**
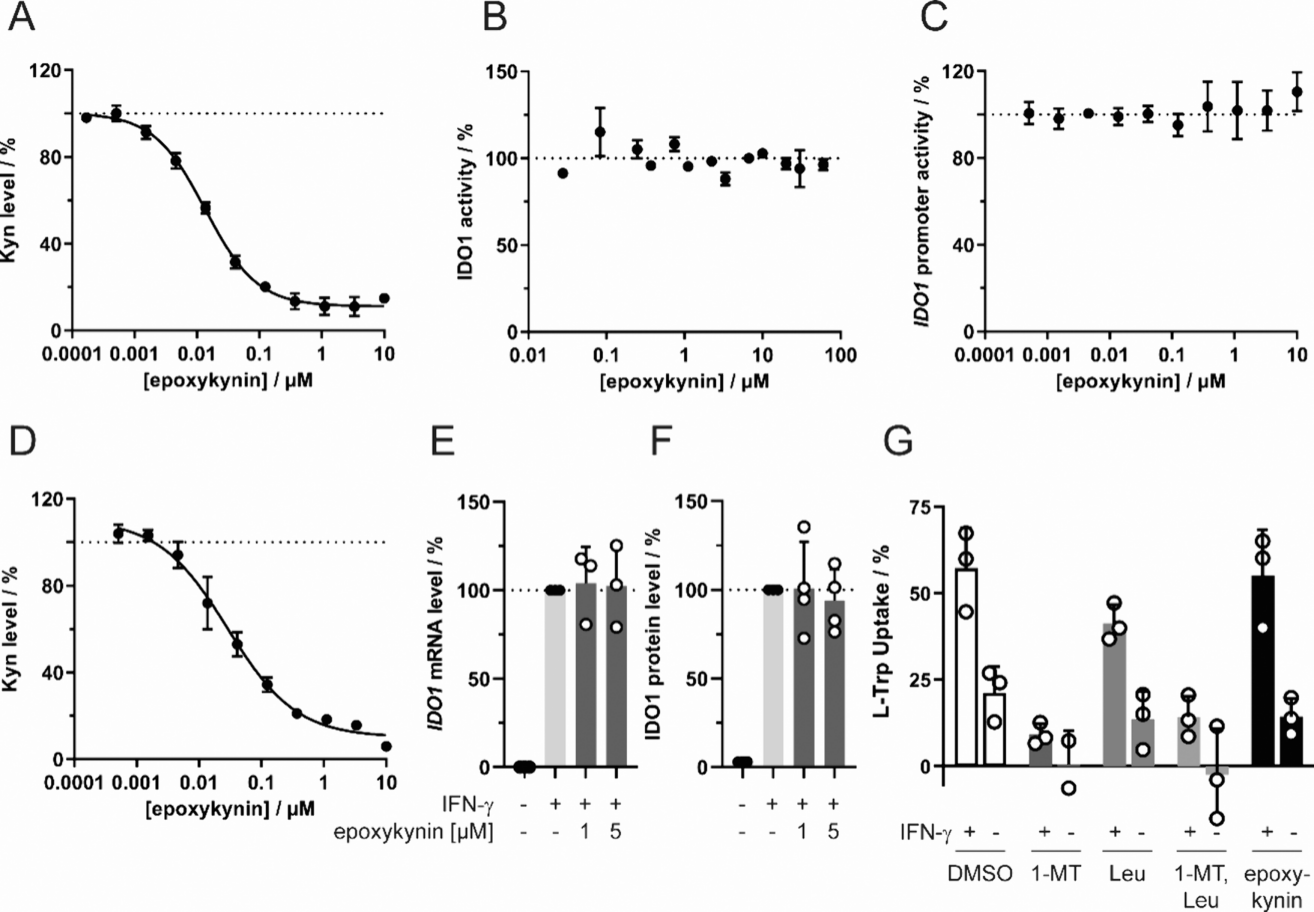
Reduction of cellular Kyn levels by epoxykynin (**1l**) and influence on IDO1 expression and Trp uptake. (A) Kyn assay in IFN-γ-treated HeLa cells. Cells were treated with IFN-γ, Trp, and epoxykynin for 48 h prior to measuring Kyn levels using *para*-dimethylaminobenzaldehyde (*p*-DMAB, mean values ± SD, *n* = 3). (B) *In vitro* IDO1 enzymatic activity. Purified IDO1 was treated with epoxykynin or DMSO for 40 min at 37 °C prior to addition of Trp and incubation for 60 min at 37 °C. Kyn levels were detected using *p*-DMAB (mean values ± SD, *n* = 2). (C) *IDO1* promoter-dependent reporter gene assay in HEK293T cells expressing firefly luciferase (Fluc) under the control of the *IDO1* promoter and constitutive *Renilla* luciferase expression (Rluc). Cells were treated with IFN-γ to induce Fluc expression and simultaneously with epoxykynin for 48 h. Fluc values were normalized to the Rluc signal (mean values ± SD, *n* = 3). (D) Kyn assay in HEK293T cells transiently expressing human IDO1. Cells were treated with Trp and epoxykynin for 24 h prior to measuring Kyn levels with *p*-DMAB (mean values ± SD, *n* = 3). (E) *IDO1* mRNA expression in HeLa cells that were treated with IFN-γ and epoxykynin or DMSO for 24 h prior to quantification of mRNA levels via qPCR (mean values ± SD, *n* = 3). (F) IDO1 protein levels in HeLa cells that were treated with IFN-γ and epoxykynin or DMSO for 24 h prior to quantification of protein levels via immunoblotting (mean values ± SD, *n* = 4). See also Figure S2 for complete blots. (G) Trp uptake in BxPC-3 cells for epoxykynin. BxPC-3 cells were starved for Trp for 72 h and treated with IFN-γ for 24 h prior to addition 5 mM l-leucine (l-Leu), 1 mM 1-methyl-l-tryptophan (1-MT), or 5 μM epoxykynin for 30 min. Afterward, 50 μM Trp was added and the Trp uptake after 30 min was quantified with HPLC-MS/MS (mean values ± SD, *n* = 3). The dotted lines indicate signals of the respective DMSO controls that were set to 100%.

Epoxykynin was approximately 4-fold more active than the initial hit compound **1a** in IFN-γ-stimulated HeLa cells (Figure S1C) and inhibited Kyn production with an IC_50_ value of 13.0 ± 1.2 nM (Figure 1A). This result is comparable to the IC_50_ determined for the screening assay in BxPC-3 cells of 36 ± 15 nM (Table 1, entry 12) and demonstrates that compound activity is not restricted to BxPC-3 cells. Similar to the original screening hit **1a**, epoxykynin did not affect the *in vitro* enzymatic activity of IDO1 (Figure 1B and Figure S1B) and did not alter *IDO1* expression as detected using a reporter gene under the control of the *IDO1* promoter (Figure 1C). Epoxykynin decreased the Kyn levels in HEK293T cells that transiently express IDO1 in the absence of IFN-γ with an IC_50_ value of 29.0 ± 8.4 nM (Figure 1D), demonstrating that the *IDO1* promoter and signaling events upstream of the promoter are not modulated by the compound. In agreement with these findings, compound treatment did not alter the *IDO1* mRNA or IDO1 protein levels (Figure 1E,F). Epoxykynin may interfere with the uptake of the IDO1 substrate Trp and thereby reduce Kyn production. Large essential amino acids, such as Trp or leucine (Leu), can be imported by l-type amino acid transporters (LAT), which can be inhibited by saturating concentrations of Leu.^39,40^ Furthermore, Trp can be transported into the cell by IFN-γ-inducible tryptophanyl-tRNA synthetases (TrpRS),^41^ and treatment with the Trp analogue 1-methyl-l-tryptophan (1-MT) inhibits TrpRS.^41−43^ To analyze both uptake routes, BxPC-3 cells were starved for Trp in the presence or absence of IFN-γ prior to treatment with Leu, 1-MT, and epoxykynin for 30 min (Figure 1G). As expected, IFN-γ increased Trp uptake due to upregulation of TrpRS and IDO1, thus leading to a higher demand for the IDO1 substrate Trp. Trp import was reduced by Leu and 1-MT, but not by epoxykynin. Hence, epoxykynin **1l** decreases cellular Kyn levels in the presence and absence of IFN-γ, but neither by reduction of IDO1 expression and inhibition of IDO1 enzymatic activity, nor by modulation of the uptake of the IDO1 substrate Trp.

**Figure 2:**
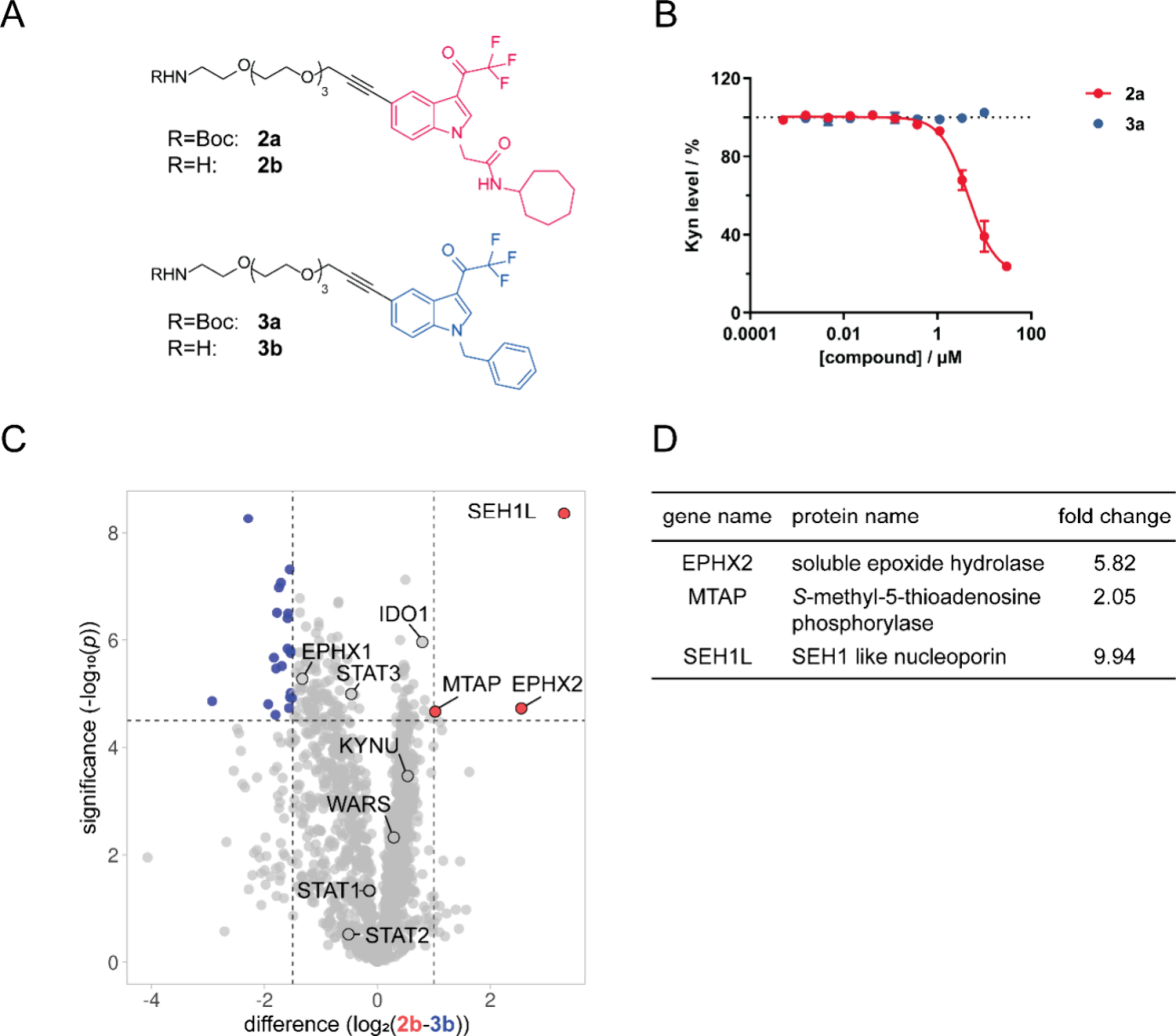
Target identification for epoxykynin. (A) Structures of affinity probes **2a** and **2b** and control affinity probes **3a** and **3b**. (B) Influence of the pulldown probes **2a** and **3a** on Kyn levels. HeLa cells were treated with IFN-γ, Trp, and compounds for 48 h prior to measuring Kyn levels using *p*-DMAB (mean values ± SD, *n* = 3). The dotted line indicates the signal of the DMSO+IFN-γ control that was set to 100%. (C) Volcano plot for proteins enriched using affinity-based chemical proteomics (pulldown) with probe **2b** (red) or control probe **3b** (blue) created with VolcaNoseR.^44^ The affinity probes **2b** and **3b** were immobilized on NHS-activated beads and incubated for 2 h at 4 °C with lysate of HeLa cells that were treated with IFN-γ. Enriched proteins were analyzed using HRMS (*n* = 2, *N* = 4, FDR 0.01), representative replicate is shown, see also Figure S3. (D) Proteins from panel (C) that were significantly enriched with the affinity probe **2b**. For a complete list of enriched proteins, see Tables S2 and S3.

Based on the structure–activity relationship analysis, affinity probes for chemical proteomics (pulldown) were generated. To this end, a Boc-protected amine-PEG4-alkyne linker was attached to epoxykynin and inactive compound **1r** (Table 1, entry 18) to form precursors **2a** and **3a**, respectively (Figure 2A), and removal of the Boc group yielded free amine probes **2b** and **3b**. Active probe **2a** decreased cellular Kyn levels, while negative probe **3a** did not (Figure 2B). Subsequently, probes **2b** and **3b** were immobilized on NHS-activated beads for the affinity pulldown and incubated with HeLa cell lysate followed by HRMS analysis of bound proteins (Figure 2C). In total, 1,579 proteins were identified but only SEH1 like nucleoporin (SEH1L), methylthioadenosine phosphorylase (MTAP), and the soluble epoxide hydrolase (sEH, EPHX2) bound selectively to active affinity probe **2b** (Figure 2C,D, Figure S3, Tables S2 and S3). Several additional proteins involved in the IDO1 pathway were found, namely, IDO1, STAT1–3,^45^ TrpRS (WARS),^42^ and kynureninase (KYNU),^46^ but were not significantly enriched by either of the affinity probes (Figure 2C and Figure S3). Unlike sEH, both MTAP and SEH1L were also enriched using the control probe **3b** to a certain extent (Table S4). The CRAPome^47,48^ database for background contaminants in affinity pulldowns lists SEH1L and other nucleoporins as frequently detected under control conditions. Thus, SEH1L might nonspecifically interact with the sample matrix, whereas sEH and MTAP may represent *bona fide* interactors. To explore MTAP as a possible target of epoxykynin, we employed HCT116 MTAP^(−/−)^ cells that transiently express IDO1 (Figure S4). Epoxykynin inhibited cellular Kyn production in both HCT116 wild-type (wt) and MTAP knockout cells with comparable IC_50_ values of 20.7 ± 11.5 and 20.5 ± 15.9 nM, respectively. Therefore, epoxykynin does not suppress Kyn levels via modulation of MTAP.

**Figure 3:**
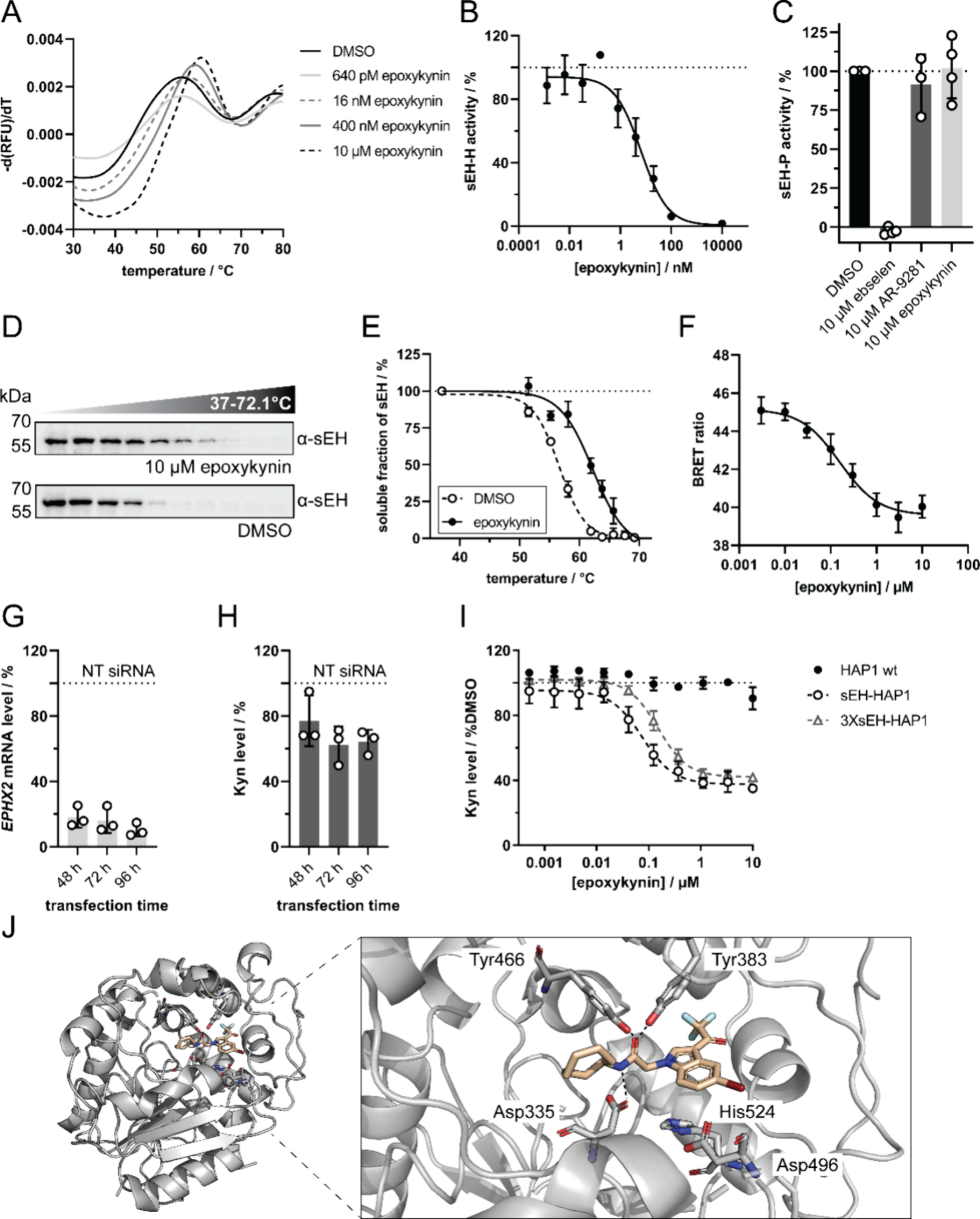
Epoxykynin binds to sEH *in vitro* and *in cellulo* and inhibits the C-terminal lipid epoxide hydrolase activity (sEH-H). (A) Dose-dependent binding of epoxykynin to sEH as detected using nanoDSF. Purified sEH was treated with epoxykynin or DMSO for 10 min at room temperature prior to detection of the intrinsic tryptophan/tyrosine fluorescence upon thermal denaturation. Representative first derivatives of melting curves are shown (*n* = 4, see also Figure S5). (B) Dose-dependent inhibition of sEH-H by epoxykynin. The epoxide hydrolase activity of purified sEH (sEH-H) was measured by means of the conversion of the fluorogenic sEH-H substrate PHOME upon treatment with epoxykynin (mean values ± SD, *n* = 3). See also Figure S6. (C) Epoxykynin does not inhibit sEH-P. The phosphatase activity of purified sEH (sEH-P) was measured by means of an AttoPhos-based assay upon treatment with epoxykynin or AR9281 and ebselen as controls. Representative curves are shown (*n* = 3, see also Figure S7). (D) Cellular thermal shift assay (CETSA) for sEH in Jurkat cells. Cells were treated with 10 μM epoxykynin or DMSO for 15 min prior to heat treatment and cell lysis. Soluble proteins were analyzed using immunoblotting. Representative immunoblots are shown (*n* = 3, see also Figure S8). (E) Thermal stability of sEH upon compound treatment. Quantification of sEH band intensities from D (mean values ± SD, *n* = 3). (F) Dose-dependent displacement of a fluorescent tracer **4**(49) by epoxykynin in HEK293T cells expressing NanoLuc-sEH. HEK293T cells that transiently express NanoLuc-sEH were treated with 60 nM of tracer^49^ and epoxykynin for 5 h prior to determination of the bioluminescence resonance energy transfer (BRET) ratio (mean values ± SD, *n* = 5). See Figure S9 for structure of fluorescent tracer **4**. (G, H) Knockdown (KD) of sEH decreases Kyn levels. HeLa cells were transfected with 50 nM nontargeting (NT) or *EPHX2*-targeting siRNA for 48–96 h and treated with Trp and IFN-γ for 48 h prior to detection of *EPHX2* mRNA (G) and Kyn levels with *p*-DMAB (H) (mean values ± SD, *n* = 3). (I) Overexpression of sEH in IFN-γ-HAP1 cells. HAP1 cells were transiently transfected with different amounts of sEH expression plasmid (1 μg (sEH-HAP1) or 3 μg (3XsEH-HAP1) plasmid DNA per 96-well plate) prior to treatment with epoxykynin, Trp and IFN-γ for 48 h. Kyn levels were quantified using *p*-DMAB (mean values ± SD, *n* = 3). The dotted lines indicate signals of the respective controls that were set to 100%. (J) Crystal structure of epoxykynin bound to human sEH-H (aa 228–547, PDB 8QZD). Epoxykynin (wheat sticks) binds to the sEH-H active site (gray cartoon and sticks) and is stabilized by polar interactions with the two stabilizing residues Tyr383 and Tyr466 and with Asp335 of the catalytic triad Asp335-Asp496-His524 (indicated by the dotted black lines). The amino acids in the active site are labeled with the three-letter code. Heteroatoms of the ligand and amino acid side chains are depicted in red (oxygen), blue (nitrogen), dark red (bromine), and cyan (fluorine). Amino acids 497–500 are omitted for clarity.

In the pulldown, sEH was selectively enriched by affinity probe **2b** in comparison to probe **3b**. Moreover, epoxykynin competed with probe **2b** for binding to sEH (Figure S3). Direct binding of epoxykynin to sEH was analyzed using nano differential scanning fluorimetry (nanoDSF, Figure 3A and Figure S4). Treatment of purified sEH with epoxykynin dose-dependently shifted the thermal denaturation temperature *T*_m_ of sEH by 5.5 ± 1.5 °C at 10 μM, suggesting binding of epoxykynin to sEH. Epoxykynin inhibited the hydrolase activity of purified sEH very potently with an IC_50_ value of 6.7 ± 3.2 nM (Figure 3B). Compounds less active in the Kyn level reduction assay were also weaker inhibitors of sEH-H (Table S5). Additional analysis of inhibition of the phosphatase domain of sEH by means of an AttoPhos-based assay^22^ showed that the sEH-H and sEH-P inhibitor ebselen,^22^ but not the sEH-H inhibitors AR9281^50^ and epoxykynin impeded the activity of sEH-P in this assay (Figure 3C). These findings demonstrate that epoxykynin selectively inhibits the C-terminal hydrolase domain of sEH, but not the N-terminal phosphatase domain. To show target engagement in cells, the thermal stability of sEH was investigated by means of a cellular thermal shift assay (CETSA, Figure 3D,E) in Jurkat cells, which have high sEH levels.^51−53^ Compared to the DMSO control, epoxykynin increased the melting temperature of sEH by 5.9 ± 1.2 °C, which correlates well with the Δ*T*_m_ determined in the nanoDSF experiment (Figure 3A). For further confirmation of cellular target engagement by nano bioluminescence resonance energy transfer (nanoBRET), a fluorescent sEH-H ligand **4** was exposed to HEK293T cells that transiently express NanoLuc-sEH (Figure 3F and Figure S9).^49^ Transfer of energy by the bioluminescent NanoLuc-sEH donor to the fluorescent acceptor **4** can only occur in close proximity.^54^ Thus, a decrease in the BRET ratio indicates displacement of tracer **4** from the NanoLuc-sEH protein. Treatment with epoxykynin dose-dependently decreased the BRET ratio with an IC_50_ value of 159.4 ± 44.0 nM (Figure 3F). These findings demonstrate that epoxykynin binds sEH both *in vitro* and in cells and inhibits sEH-H but does not impair the catalytic activity of sEH-P. In addition, depletion of sEH in HeLa cells with siRNA resulted in sEH knockdown of 82 ± 5%, 84 ± 6%, and 90 + 3% after 48, 72, and 96 h, respectively (Figure 3G). This partial knockdown decreased Kyn levels by 23 ± 13%, 38 ± 9%, and 36 ± 6% after 48, 72, and 96 h, respectively (Figure 3H). Hence, depletion of sEH phenocopies treatment with epoxykynin. We noticed that epoxykynin did not affect IFN-γ-induced Kyn production in IFN-γ-HAP1 cells, whereas IDO1 inhibitors like epacadostat reduced Kyn levels (Figure S10). HAP1 cells express hardly any sEH,^51−53^ which explains the inactivity of epoxykynin in this cell line and makes it particularly useful for sEH overexpression studies. Accordingly, treatment of IFN-γ-HAP1 cells that transiently express sEH with epoxykynin inhibited Kyn production with an IC_50_ value of 68.4 ± 9.7 nM (Figure 3I). Tripling the amount of transfected plasmid DNA shifted the IC_50_ to 159.7 ± 49.1 nM (Figure 3I). These findings prove sEH as the target of epoxykynin that mediates the reduction in Kyn levels.

A cocrystal structure of epoxykynin with human sEH-H was obtained to confirm the binding mode of the compound (Figure 3J, PDB 8QZD). The active site of sEH-H consists of the catalytic triad Asp335-Asp496-His524 as well as the two stabilizing residues Tyr381 and Tyr465 opposite of the catalytic triad serving as an oxyanion hole.^55^ The crystal structure revealed that epoxykynin binds to the catalytic center of sEH-H, occupying the binding site of the endogenous epoxide substrates. The ligand is stabilized by hydrogen bonds between the amide oxygen of epoxykynin and residues Tyr383 and Tyr466 and an additional hydrogen bond between the amide nitrogen of epoxykynin and Asp335 of the catalytic triad (Figure 3J). The cocrystal structure elucidates general trends in the SAR of the epoxykynin derivatives. The *N*-alkyl amide binds to the catalytic triad of sEH-H, while the lipophilic cycloheptane occupies a deep hydrophobic pocket. The constraint indole ring creates distance to the small, electron-withdrawing bromo and trifluoroacetyl substituents.

Structural alignments with previously published structures of sEH-H (Figure S11A–D, PDBs 1S8O, 5AI5, 3WKE, 4HAI) showed high overall similarity with root-mean-square deviation (RMSD) values below 1 Å. Besides the flexible C-terminus, there are minor structural deviations in a disordered loop of the cap domain of sEH-H between residues Ala411 and Lys421 (Figure S11E), indicating structural flexibility upon binding of different ligands. The two nitrogens of urea-derived sEH-H inhibitors (Figure S11F) act as hydrogen bond donors, while amide-based sEH-H inhibitors, such as epoxykynin (Figure 3J), only contain a single nitrogen as potential hydrogen bond donor to Asp335 of the catalytic triad. Yet, not all amide-based sEH-H inhibitors are stabilized by this hydrogen bond in the active site (Figure S11G,H).

Additionally, we tested the urea sEH-H inhibitors AR-9281^50^ and 1-trifluoromethoxyphenyl-3-(1-propionylpiperidin-4-yl)urea^56^ (TPPU) as well as the sEH-P inhibitor SWE101^57^ and sEH-H and sEH-P ebselen^22^ for Kyn reduction in IFN-γ-treated HeLa cells (Figure S12). AR-9281 and TPPU did not inhibit cellular Kyn production (Figure S12A,S12B). Poor pharmacokinetic properties and short drug-target residence times of these urea inhibitors may contribute to the lack of activity in the cellular Kyn assay.^56^ As expected, the sEH-P inhibitor SWE101 did not reduce cellular Kyn levels (Figure S12C). Only ebselen decreased Kyn levels by 43.8 ± 3.6% at 10 μM (Figure S12D). Ebselen is a Cys-reactive compound that targets sEH-P and cooperatively inhibits sEH-H by conformational changes.^22^ It is known to have multiple targets^58^ and, thus, more selective compounds, such as epoxykynin, are better suited to study the effect of sEH-H inhibition in cellular systems.

**Figure 4:**
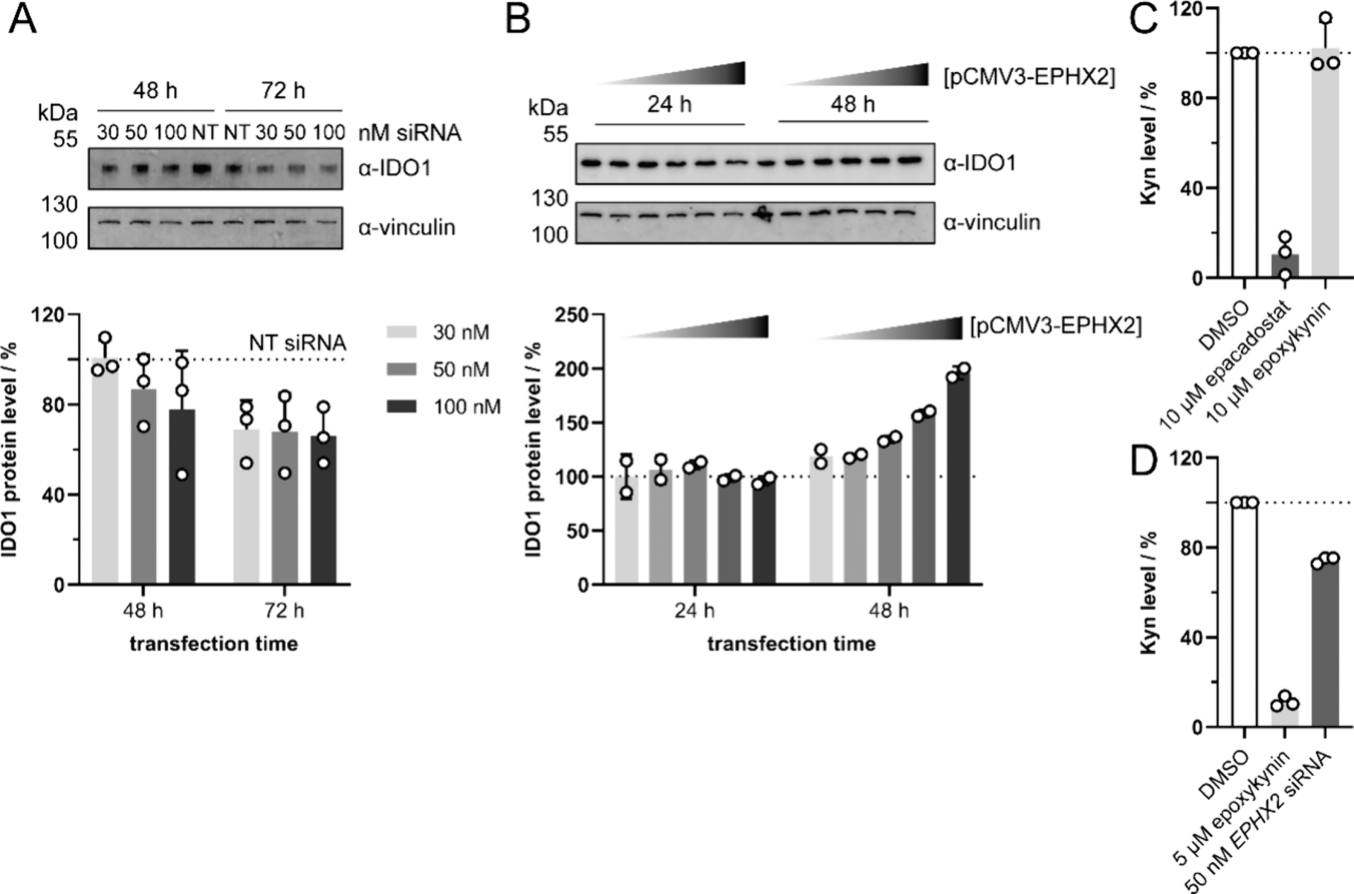
sEH cross-talks with the Kyn pathway, thereby modulating cellular IDO1 and Kyn levels. (A) Knockdown (KD) of sEH. HeLa cells were transfected with nontargeting (NT) or *EPHX2*-targeting siRNA for 48–72 h and treated with IFN-γ for 48 h prior to quantification of IDO1 protein levels via immunoblotting (mean values ± SD, *n* = 3). See also Figure S13 for complete blots. (B) Overexpression of sEH. HeLa cells were transfected with empty vector or pCMV3-EPHX2 for 24–48 h and treated with IFN-γ for 48 h prior to quantification of IDO1 protein levels via immunoblotting (mean values ± SD, *n* = 2). See also Figure S14 for complete blots. (C–D) Kyn assay in cell lysate. (C) BxPC-3 cells were treated with IFN-γ for 24 h prior to cell lysis. The lysate was treated with 10 μM epacadostat, 10 μM epoxykynin or DMSO for 45 min prior to detection of Kyn levels (mean values ± SD, *n* = 3). (D) BxPC-3 cells were treated with 5 μM epoxykynin, 50 nM *EPHX2*-targeting siRNA or DMSO and IFN-γ for 48 h prior to cell lysis and detection of Kyn levels (mean values ± SD, *n* = 3).

The metabolic level of reactive epoxides needs to be precisely balanced by biological systems,^24^ and as a member of the arachidonic acid pathway, sEH is involved in regulation of their levels. Kreiß et al. recently demonstrated that arachidonic acid metabolizing human 5-lipoxygenase regulates the expression of kynureninase,^59^ which suggests a possible functional link between the second branch of the AA acid cascade and the Kyn pathway. To investigate if IDO1 protein levels are regulated by sEH protein, we analyzed the IDO1 level after depletion (Figure 4A) or overexpression of sEH (Figure 4B) in HeLa cells. Knockdown of sEH decreased cellular IDO1 levels by 35 ± 10% after 72 h (Figure 4A), while overexpression of sEH increased IDO1 levels after 48 h, but not after 24 h (Figure 4B). In line with these findings, Zhou et al. found increased *IDO1* mRNA levels in sEH-overexpressing HCT116 cells,^60^ which links sEH to regulation of Kyn levels through IDO1. We then explored whether epoxykynin would still modulate Kyn production when added to a lysate of IFN-γ-stimulated BxPC-3 cells. For example, the IDO1 inhibitor epacadostat reduces Kyn levels when added to cells or to a lysate of IDO1-expressing cells. In contrast, epoxykynin did not affect Kyn levels when added after cell lysis (Figure 4C). This result demonstrates that acute inhibition of sEH in cell lysate does not decrease Kyn levels. However, treatment of BxPC-3 cells with either epoxykynin or *EPHX2*-targeting siRNA decreased Kyn levels (Figure 4D). These findings indicate that inhibition or depletion of sEH in live cells and over a longer period of time is required to modulate Kyn production. A cross-talk between both pathways may operate *in cellulo* since sEH modulates the Kyn pathway and alters IDO1 protein levels.

Kyn is an endogenous agonist of the ligand-activated aryl hydrocarbon receptor (AhR).^61^ Activation of AhR induces IDO1 expression in antigen-presenting cells (APCs) or IDO1-positive cancer cells, promoting long-term immune tolerance.^62,63^ AhR antagonists can decrease plasma levels of AA in mice, while, vice versa, Kyn administration increases AA levels.^64^ Furthermore, the AA metabolite 12(*R*)-hydroxy-5(*Z*),8(*Z*),10(*E*),14(*Z*)-eicosatetraenoic acid (12(*R*)-HETE) activates AhR.^65^ Considering these findings, the connection between sEH overexpression and increased IDO1 levels reported here and noted previously^57^ may be due to an AhR-mediated feedback loop. This hypothesis is supported by the fact that inhibition of sEH by epoxykynin in cell lysate is not sufficient to inhibit Kyn production in lysates, indicating a possible functional cross-regulation of sEH and IDO1 expression by AhR. Since the AA derivative 12(*R*)-HETE acts as an AhR agonist,^65^ other eicosanoids may also bind to AhR, such as the sEH substrate epoxyeicosatrienoic acids (EETs) and dihydroxyeicosatrienoic acids (DHETs).^66^

## Conclusions

sEH has been identified as a promising target for medicinal chemistry programs, and two sEH-targeting compounds have progressed to clinical trials, but have not found regulatory approval,^56,67,68^ such that new approaches are in high demand. To this end, compound identification by means of a less biased cell-based assay may open up new advantageous alternatives to existing approaches. In conclusion, by means of a phenotypic screening for modulators of cellular Kyn production and subsequent target identification and validation, we have identified the potent sEH inhibitor epoxykynin. The compound engages with sEH in cells and inhibits sEH-H, resulting in reduced Kyn levels in cells expressing IDO1. Our data suggest a functional link between the third branch of the AA cascade and the Kyn pathway. Since the direct IDO1 inhibitor epacadostat recently failed clinical trials, alternative approaches to reduce Kyn levels are coveted, and inhibition of the Kyn pathway by modulating sEH may open up novel opportunities to revert cancer-related immune suppression.

## Experimental Section

### Chemical Synthesis

#### General Information

The screening library of in total 157,332 compounds was composed of proprietary (10%) and commercially available compounds (90%). The commercial compounds included reference small molecules and natural products with annotated targets as well as compounds with drug-likeness and accordance to Lipinski’s rule of five. Epoxykynin derivatives were purchased from ChemDiv, US and are >95% pure upon purchase.

Unless otherwise stated, all commercially available compounds were used as provided without further purification. Solvents for chromatography were technical grade.

^1^H NMR, ^13^C NMR, and ^19^F-NMR were recorded on Bruker DRX400 (400 MHz), Bruker DRX500 (500 MHz), INOVA500 (500 MHz), and Bruker DRX700 using CD_2_Cl_2_ as solvent and trimethoxybenzene as internal standard. Data is reported in the following order: chemical shift values (δ) are reported in ppm with the solvent resonance as internal standard (CD2Cl2: δ = 5.32 ppm for ^1^H, δ = 54.00 ppm for ^13^C). Multiplicities are indicated as br s: broad singlet, s: singlet, d: doublet, t: triplet, q: quartet, m: multiplet. Coupling constants (J) are given in Hertz.

HPLC-MS spectra were acquired using the LTQ Fleet (Thermo Fisher Scientific, US), Ultimate 3000 HPLC (Thermo Fisher Scientific, US), and Xcalibur software (Thermo Fisher Scientific, US).

For chromatography, solutions A (ddH2O + 0.1% (v/v) formic acid) and B (acetonitrile +0.1% (v/ 422 v) formic acid) were used with a flow rate of 0.4 mL/min. The gradient started with 10% B and 90%A for 0.5 min. Afterwards, a linear gradient of B was increased upto 95% in 7 min. The column was washed using 95 % B and re-equilibrated to 10% B. A: ddH2O + 0.1% (v/v) formic acid; B: acetonitrile +0.1% (v/v) formic acid; flow rate:0.4 mL/min.

#### Synthesis of the Affinity Pulldown Probes

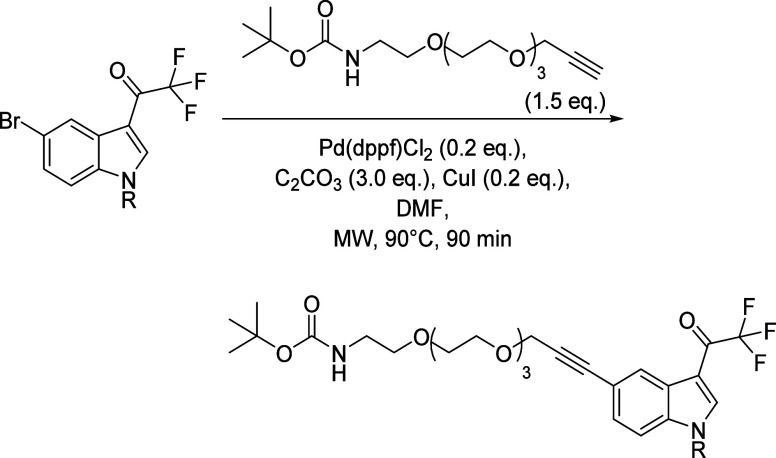



Into a reaction vessel were loaded indole (1.0 equiv), the Pd(dppf)Cl_2_ catalyst (0.2 equiv), C_2_CO_3_ (3.0 equiv), and CuI (0.2 equiv). The vessel was backfilled thrice with argon. 40 mL of anhydrous DMF (0.05 M) was then added. Again, the vessel was backfilled thrice with argon. The mixture was stirred prior to addition of previously degassed amine-alkyne-PEG4 linker (1.5 equiv) in a minimal amount of DMF. The reaction mixture was heated to 90 °C for 90 min in a microwave reactor.

The mixture was poured into 20 mL of 1 N HCl, extracted with 50 mL of ethyl acetate and washed with brine. The organic phase was separated from the aqueous phase and washed with 20 mL of brine and dried over Na_2_SO_4_ and concentrated *in vacuo*. The crude material was purified by silica gel column chromatography (Pent/EtOAc (1:1 to 1:2)) to yield a brown solid.

#### Deprotection of the Affinity Pulldown Probes

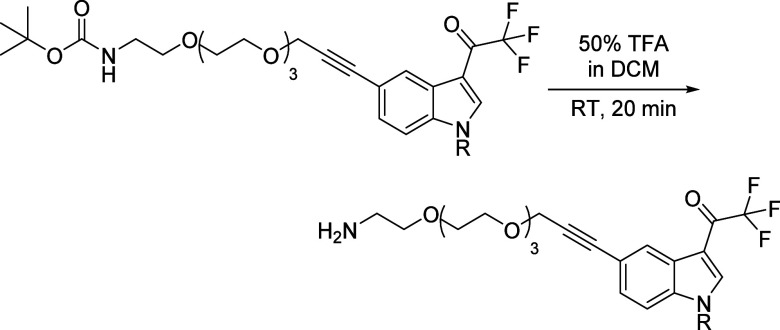



The boc-protected starting material was dissolved in 50% (v/v) TFA in DCM and stirred for 20 min at room temperature. The product was concentrated thrice with toluene *in vacuo* and lyophilized to afford the free amine probe.

### Biological Methods

#### Cell Culture

Human cell lines were maintained at 37 °C and 5% CO_2_ in a humidified atmosphere and subcultivated twice a week. All cell lines were tested regularly for mycoplasma contamination and were always free of mycoplasma. BxPC-3 cells (DSMZ#760, female, RRID:CVCL_0186) and Jurkat cells (DSMZ#282, male, RID:CVCL_0065) were grown in RPMI-1640 medium supplemented with 10% FBS, 2 mM l-glutamine, 1 mM sodium pyruvate, 4.5 g/L glucose, 10 mM HEPES and 1.5 g/L NaHCO_3_. HeLa (DSMZ#57, female, RRID: CVCL_0030), HCT116 (DSMZ#581, male, RRID:CVCL_0291), HCT116 MTAP^(−/−)^ (Horizon Discovery#HD R02–033, male, parental RRID:CVCL_0291), and HEK293T cells (ATCC#11268, female, RRID:CVCL_1926) were cultivated in DMEM supplemented with 10% FBS, 4.5 g/L glucose, 4 mM l-glutamine, 1 mM sodium pyruvate, 1% nonessential amino acids, 3.7 g/L NaHCO_3_. HAP1 cells (Horizon Discovery#c631, male, RRID:CVCL_Y019) were maintained in IMDM medium supplemented with 10% FBS, 4.5 g/L glucose, 4 mM l-glutamine, 25 mM HEPES, and 3.0 g/L NaHCO_3_.

### Kynurenine (Kyn) Assays

#### High-Throughput Kyn Assay

Automated screening for modulators of Kyn levels in BxPC-3 cells was performed as published earlier.^34^ Briefly, BxPC-3 cells (1,000 cells/well) were seeded in black 1536-well plates prior to incubation for 24 h and subsequent treatment with compounds, 380 μM Trp, and 50 ng/mL IFN-γ for 48 h. Afterward, trichloroacetic acid was added to a final concentration of 7% (v/v), the plates were incubated for 10 min at 37 °C and centrifuged for 10 min at 1620*g*. Kyn was detected by addition of 17.5 μM Kyn sensor^69^ in assay buffer (excitation: 535 nm, emission: 595 nm). In total, 157,332 compounds were screened at a concentration of 7.1 μM. The screening library (157,332 compounds) comprised 90% commercial (e.g., LOPAC and Prestwick Chemical Libraries) and 10% in-house synthesized compounds. The epoxykynin compound class was purchased from ChemDiv, US. Compounds that reduced Kyn levels by ≥70% were subjected to IC_50_ determination. Cytotoxic compounds were excluded by nuclear staining with Hoechst 33342 prior to incubation for 30 min at 37 °C and imaging with the ImageXpress Micro XL (excitation: 377/50 nm, emission: 447/60 nm, Molecular Devices, US). Image analysis was performed by means of cell count with the Cell Proliferation HT Application Module of the MetaXpress software (Molecular Devices, US).

#### Manual Kyn Assay

For manual testing, BxPC-3 or HeLa cells (20,000 cells/well or 5,000 cells/well, respectively) were seeded in 96-well plates in medium without phenol red. After 24 h, the Kyn pathway was induced by addition of 50 ng/mL IFN-γ and 380 μM (BxPC-3) or 164.15 μM (HeLa) Trp with simultaneous treatment with the compounds at the indicated concentrations followed by incubation for 48 h. Subsequently, trichloroacetic acid was added to a final concentration of 7% (v/v), the plates were incubated for 15 min at room temperature and centrifuged for 10 min at 1800*g*. For determination of Kyn levels with the Ehrlich reagent, an equal volume of freshly prepared 2% (w/v) *p*-DMAB in glacial acetic acid (Ehrlich reagent) was added prior to measuring the absorbance at 492 and 650 nm on the Spark Multimode Microplate Reader (Tecan, AT). The background absorbance A_650_ was subtracted from the absorbance A_492_ of the Kyn-*p*-DMAB adduct and normalized to the DMSO control. Data analysis was performed using a nonlinear regression curve fit and GraphPad Prism 9.0 (GraphPad Software, Inc., US) to generate dose–response curves and obtain IC_50_ values.

#### Determination of Kyn Levels Using LC-MS

For determination of Kyn levels via LC-MS, BxPC-3 (20,000 cells/well) were seeded in 96-well plates in medium without phenol red. After 24 h, the Kyn pathway was induced by addition of 50 ng/mL IFN-γ and 380 μM Trp with simultaneous treatment with the compounds at the indicated concentrations followed by incubation for 48 h. Subsequently, trichloroacetic acid was added to a final concentration of 7% (v/v), the plates were incubated for 15 min at room temperature and centrifuged for 10 min at 1800*g*. Kyn and Trp was quantified by HPLC-MS/MS using the LTQ Velos Pro and Dionex HPLC (Thermo Fisher Scientific, US). Data analysis was performed with Xcalibur (Thermo Fisher Scientific, US) and represented using GraphPad Prism 9.0 (GraphPad Software, Inc., US).

#### Kyn Assay in IDO1-HEK293T Cells

HEK293T cells (25,000 cells/well) were reverse-transfected with pCMV3-IDO1 (1 μg/96-well plate, Sino Biological, CN) using 3.9 μL of Lipofectamine 2000 (Invitrogen, US) prior to incubation for 20 h. Subsequently, cells were treated with 500 μM Trp and the compounds for 24 h. Thereafter, trichloroacetic acid was added to a final concentration of 7% (v/v), the plates were incubated for 15 min at room temperature and centrifuged for 10 min at 1800*g*. Afterward, an equal volume of freshly prepared 2% (w/v) *p*-DMAB in glacial acetic acid (Ehrlich reagent) was added prior to measuring the absorbance at 492 and 650 nm on the Spark Multimode Microplate Reader (Tecan, AT). To determine the Kyn levels, the background absorbance A_650_ was subtracted from the absorbance A_492_ of the Kyn-*p*-DMAB adduct and normalized to the DMSO control. Data analysis was performed using a nonlinear regression curve fit and GraphPad Prism 9.0 (GraphPad Software, Inc., US) to generate dose–response curves and obtain IC_50_ values.

#### Kyn Assay in IDO1-HCT116 Cells

HCT116 wt and HCT116 MTAP^(−/−)^ cells (30,000 cells/well) were reverse-transfected with pCMV3-IDO1 (2 μg/96-well plate, Sino Biological, CN) using 4 μL of Lipofectamine 3000 and 4 μL of P3000 reagent (Invitrogen, US) prior to incubation for 24 h. Subsequently, cells were treated with 500 μM Trp and the compounds for 36 h. Thereafter, trichloroacetic acid was added to a final concentration of 7% (v/v), the plates were incubated for 15 min at room temperature and centrifuged for 10 min at 1800*g*. Afterward, an equal volume of freshly prepared 2% (w/v) *p*-DMAB in glacial acetic acid (Ehrlich reagent) was added prior to measuring the absorbance at 492 and 650 nm on the Spark Multimode Microplate Reader (Tecan, AT). To determine the Kyn levels, the background absorbance A_650_ was subtracted from the absorbance A_492_ of the Kyn-*p*-DMAB adduct and normalized to the DMSO control. Data analysis was performed using a nonlinear regression curve fit and GraphPad Prism 9.0 (GraphPad Software, Inc., US) to generate dose–response curves and obtain IC_50_ values.

#### Kyn Assay in sEH-HAP1 Cells

HAP1 cells (20,000 cells/well) were reverse-transfected with pCMV3-EPHX2 (1 and 3 μg/96-well plate, Sino Biological, CN) using 2 μL of Lipofectamine 3000 and 2 μL of P3000 reagent (Invitrogen, US) prior to incubation for 20 h. Subsequently, cells were treated with 10 ng/mL IFN-γ and 100 μM Trp and the compounds for 48 h. Thereafter, trichloroacetic acid was added to a final concentration of 7% (v/v), and the plates were incubated for 15 min at room temperature and centrifuged for 10 min at 1800*g*. Afterward, an equal volume of freshly prepared 2% (w/v) *p*-DMAB in glacial acetic acid (Ehrlich reagent) was added prior to measuring the absorbance at 492 and 650 nm on the Spark Multimode Microplate Reader (Tecan, AT). To determine the Kyn levels, the background absorbance A_650_ was subtracted from the absorbance A_492_ of the Kyn-*p*-DMAB adduct and normalized to the DMSO control. Data analysis was performed using a nonlinear regression curve fit and GraphPad Prism 9.0 (GraphPad Software, Inc., US) to generate dose–response curves and obtain IC_50_ values.

#### In Vitro Kyn Assay

To detect direct inhibition, 1 μM recombinant human IDO1 was incubated with compounds for 40 min at 37 °C in 50 mM potassium phosphate buffer (16.9 mM K_2_HPO_4_, 33.1 mM, KH_2_PO_4_, pH 6.5). Subsequently, 10 mM ascorbic acid, 10 μM methylene blue, 2 mM Trp, and 100 μg/mL catalase were added and samples were incubated for 60 min at room temperature or 37 °C. Trichloroacetic acid was added to a final concentration of 7% (v/v), and samples were incubated for 30 min at 70 °C. Afterward, an equal volume of freshly prepared 2% (w/v) *p*-DMAB in glacial acetic acid (Ehrlich reagent) was added prior to measuring absorbance at 492 and 650 nm on the Spark Multimode Microplate Reader (Tecan, AT). To determine the Kyn levels, the background absorbance A_650_ was subtracted from the absorbance A_492_ of the Kyn-*p*-DMAB adduct and normalized to the DMSO control. Data analysis was performed using a nonlinear regression curve fit and GraphPad Prism 9.0 (GraphPad Software, Inc., US) to generate dose–response curves and obtain IC_50_ values.

#### Kyn Assay in Lysates

The Kyn assay in lysate was performed using the commercial Indoleamine 2,3-Dioxygenase 1 (IDO1) Activity Assay Kit (cat# K972–100, BioVision Inc., US). Therefore, BxPC-3 cells (1 × 10^6^ cells) were seeded in 60 mm-dishes and incubated for 24 h. On the next day, cells were treated with 50 ng/mL IFN-γ prior to incubation for 48 h. If the cells were pretreated with compound or siRNA, the compounds or siRNA were added simultaneously with IFN-γ. The plates were washed twice with warm PBS prior to harvesting the cells with 750 μL trypsin/EDTA solution. The cells were washed with ice-cold PBS and collected by centrifugation at 4 °C and 300*g* for 5 min prior to resuspension in 300 μL of IDO1 assay buffer containing protease inhibitors. The cells were lysed by three consecutive freeze/thaw cycler.

For the Kyn detection, the 2× reaction premix was prepared by diluting the antioxidant mix 50-fold in IDO1 assay buffer. A 10× working solution of the IDO1 substrate Trp was prepared by 10-fold dilution of the stock in IDO1 assay buffer (final Trp concentration in the assay: 100 μM). In a final volume of 11 μL containing 5.5 μL of 2× reaction premix, 7 μg of cell lysate was treated with the compounds prior to reaction by addition of 1.1 μL of 10× Trp. The plate was sealed with a sticky silver sealer and incubated for 45 min at 37 °C and 300 rpm in the dark prior to addition of 5 μL of the fluorogenic developer solution. The plate was incubated for another 3 h at 45 °C and 300 rpm in the dark. After cooling down for at least 1 h, the fluorescence was measured at an excitation wavelength of 402 nm and an emission wavelength of 488 nm on the Spark Multimode Microplate Reader (Tecan, AT).

### Expression and Purification of Recombinant IDO1

Recombinant human GST-IDO1 (rhIDO1) was expressed in *E. coli* BL21 DE3 cells using a pGEX6p-2rbs-GST-IDO1 and purified as published previously.^70,71^ Bacteria were transformed with the vector by a heat shock at 42 °C for 60 s and cells derived from a single colony were used to inoculate LB medium containing 100 μg/mL ampicillin and 30 μg/mL chloramphenicol prior to incubation overnight at 37 °C with rotation. On the next day, 2,500 mL of LB medium was inoculated with 50 mL of the bacteria suspension and IDO1 protein expression was induced by addition of 200 μM isopropyl β-d-1-thiogalactopyranoside prior to incubation at 18 °C overnight.

Afterward, the cells were pelleted by centrifugation at 3600*g* and 4 °C for 20 min and resuspended in buffer 1 (50 mM TRIS-HCl, pH 7.4, 100 mM NaCl) and homogenized by sonication followed by mechanical cell lysis. The soluble fraction was separated from the cell debris by centrifugation at 13,000*g* and 10 °C for 35 min and transferred to a GSTrap HP column (Cytiva, US). GST-IDO1 was washed on column with buffer 2 (50 mM TRIS-HCl, pH 7.4, 100 mM KCl) followed by on-column cleavage overnight with 3.2 mg/mL PreScission protease (Cytiva, US) in buffer 3 (50 mM TRIS-HCl, pH 7.0, 150 mM NaCl, 1 mM DTE, 200 μM hemin). The eluted protein was purified by size exclusion chromatography with a HiLoad 16/600 Superdex 200 pg column (Cytiva, US) and purity of the protein was confirmed by SDS-PAGE.

### *IDO1* Promoter Reporter Gene Assay

HEK293T cells (25,000 cells/well) were reverse-transfected with pXPG-IDO1^72^ (4 μg/96-well plate, kindly provided by Gina M. Doody, Leeds, UK) and pRL-TK (300 ng/96-well plate, Promega, US) using 12.9 μL of Lipofectamine 2000 (Invitrogen, USA) prior to incubation for 24 h. Afterward, cells were treated with 50 ng/mL IFN-γ and the compounds for 48 h. Luminescence generated by the Fluc reporter and the control reporter Rluc was measured using the Dual-Glo Luciferase Assay System (Promega, US) on the Spark Multimode Microplate Reader (Tecan, AT). To determine the *IDO1* promoter activity, Fluc signals were divided by the Rluc signals and normalized to the values of the DMSO control. Data analysis was performed using a nonlinear regression curve fit and GraphPad Prism 9.0 (GraphPad Software, Inc., US) to generate dose–response curves and obtain IC_50_ values.

### Trp Uptake Assay

BxPC-3 cells (30,000 cells/well) were seeded in 96-well plates in Trp-free RPMI 1640 medium and incubated for 48 h prior to addition of 50 ng/mL IFN-γ. Trp starvation was continued for another 24 h. Afterward, the medium was exchanged for Trp-free medium containing the control inhibitors 5 mM l-Leu (inhibitor of system-lamino acid transporters (LAT)), 1 mM 1-methyl-l-tryptophan (inhibitor of tryptophanyl-tRNA synthetase (TrpRS)), and compounds, and cells were incubated for 30 min. Subsequently, 50 μM Trp was added and samples were incubated for another 30 min. The supernatant was transferred to a new plate, trichloroacetic acid was added to a final concentration of 7% (v/v), and plates were incubated for 15 min at room temperature. Samples were centrifuged for 10 min at 1800*g* and Trp was quantified by HPLC-MS/MS using the LTQ Velos Pro and Dionex HPLC (Thermo Fisher Scientific, US). Data analysis was performed with Xcalibur (Thermo Fisher Scientific, US) and represented using GraphPad Prism 9.0 (GraphPad Software, Inc., US).

### RNA Purification and RT-qPCR

HeLa cells (250,000 cells/well) were seeded in 6-well plates and incubated for 24 h prior to addition of 50 ng/mL IFN-γ and compounds. After 24 h, RNA was extracted using the RNeasy Plus Mini Kit (QIAGEN, DE) following the manufacturer’s procedure. DNA was removed on column by DNase digestion with RNase-free DNase Set (QIAGEN, DE) according to the manufacturer’s instructions. Following the manufacturer’s protocol, cDNA templates were synthesized from 800 ng of total RNA using the QuantiTect Reverse Transcription Kit (QIAGEN, DE).

The expression levels of the *IDO1* gene and the reference *GAPDH* were assessed by reverse trancription quantitative PCR (qPCR). Therefore, 100 ng of cDNA was amplified using 500 nM of gene-specific primers and SsoAdvanced Universal SYBR Green Supermix (Bio-Rad Laboratories, DE) in a total volume of 10 μL for 50 cycles using the CFX96 Touch Real-Time PCR Detection System (Bio-Rad Laboratories, DE). Relative *IDO1* and *EPHX2* expression levels were calculated using the ΔΔ*C*t method with *GAPDH* as the reference gene^73^ and represented using GraphPad Prism 9.0 (GraphPad Software, Inc., US).

The sequences of the primers for *IDO1* were 5′-GCCTGATCTCATAGAGTCTGGC-3′ (forward) and 5′-TGCATCCCAGAACTAGACGTGC-3′ (reverse). The sequences of the primers for *EPHX2* were 5′-CCTTCATACCAGCAAATCCCAACA-3′ (forward) and 5′-TTCAGCCTCAGCCACTCCT-3′ (reverse).^74^ The sequences of the primers for *GAPDH* were 5′-GTCTCCTCTGACTTCAACAGCG-3′ (forward) and 5′-ACCACCCTGTTGCTGTAGCCAA-3′ (reverse).

### Immunoblotting

HeLa cells (250,000 cells/well) were seeded in 6-well plates and incubated for 24 h prior to addition of 50 ng/mL IFN-γ and compounds. After 24 h, cells were lysed using 1× Laemmli buffer (8% glycerol, 4.4% 0.5 M TRIS, pH 6.8, 3.1 mM SDS, 4.44 mM DTT, 33.2 mM bromophenol blue) and homogenized by sonication. Protein concentration was determined using the DC Protein Assay (Bio-Rad Laboratories, DE). 20 μg to 100 μg total protein was separated using 10% polyacrylamide gels under reducing/denaturing conditions in a TRIS/glycine-based system. Proteins were transferred to a PVDF membrane for 60 min at 100 V (Thermo Fisher Scientific, USA) using wet tank transfer (192 mM glycine, 25 mM TRIS, 10% methanol) in a Mini Trans-Blot Cell (Bio-Rad Laboratories, DE). Afterward, membranes were blocked with 5% nonfat milk in PBS with 0.1% (v/v) Tween-20 (PBS-T) or 50% Odyssey Blocking Buffer (PBS) (LI-COR Biosciences, US) in PBS-T for 60 min at room temperature followed by overnight incubation at 4 °C with the primary antibodies. For detection of IDO1, sEH, and the reference protein vinculin, the primary antibodies anti-IDO1 (1:5,000 in 5% milk in PBS-T, ab211017, Abcam, UK, RRID:AB_2936946), anti-sEH (1:1,000 in 50% Odyssey Blocking Buffer in PBS-T, A1885, ABclonal Science, US, RRID:AB_2763918), and antivinculin (1:10,000 in 5% milk in PBS-T or 50% Odyssey Blocking Buffer in PBS-T, V9131, Merck KGaA, DE, RRID:AB_477629) were used. Protein bands were visualized with secondary antibodies conjugated to IRDye Infrared Fluorescent Dyes (1:5,000 in 50% Odyssey Blocking Buffer in PBS-T, LI-COR Biosciences, US) using the ChemiDoc MP Imaging System (Bio-Rad Laboratories, DE). IDO1 and sEH protein levels were normalized to the levels of the reference protein vinculin.

### Affinity-Based Chemical Proteomics (Pulldown)

HeLa cells (5.54 × 10^5^ cells/flask) were seeded in T175 flasks and incubated for 72 h at 37 °C and 5% CO_2_ in a humidified atmosphere. The medium was exchanged for fresh culture medium containing 50 ng/mL IFN-γ prior to incubation for 24 h. The cells were harvested by trypsinization, resuspended in ice-cold PBS, and washed thrice in ice-cold PBS. The cell pellets were resuspended in 500 μL of NP-40-based lysis buffer (150 mM sodium chloride, 0.4% NP-40 alternative, 50 mM TRIS-HCl, pH 8.0), incubated on ice for 30 min and ultracentrifuged for 20 min at 100,000*g* and 4 °C.

25 μL of NHS Mag Sepharose beads (cat# 28–9440–09, Cytiva, US) were equilibrated in 500 μL of ice-cold 1 mM HCl, the equilibration solution was removed and 500 μL of 10 μM free amine probes **2b** and **3b** (0.1% (v/v)) in 150 mM triethanolamine, 500 mM sodium chloride, pH 8.3 were added. The beads were coated with the probes by overhead rotation for 1 h at room temperature. Subsequently, the residual active groups were quenched by (i) washing with 500 μL block buffer 1 (500 mM ethanolamine, 500 mM sodium chloride, pH 8.3), (ii) washing with 500 μL block buffer 2 (100 mM sodium acetate, 500 mM sodium chloride, pH 4.0), (iii) addition of 500 μL block buffer 1 and incubation for 15 min with overhead rotation, (iv) washing with 500 μL block buffer 2, (v) washing with 500 μL block buffer 1, and (vi) washing with 500 μL block buffer 2.

After the compound immobilization, the beads were washed with 500 μL of NP40-based lysis buffer prior to incubation with cell lysate (500 μL of lysate with a protein concentration of 3 g/L) for 2 h at 4 °C with overhead rotation. For the competition experiment, the cell lysate was preincubated with 10 μM unmodified epoxykynin for 1 h at room temperature prior to addition to the coated beads. Subsequently, the supernatant was removed and the beads were washed twice with 50 mM PIPES (pH 7.4), 50 mM sodium chloride, 75 mM magnesium chloride, 5 mM EGTA, 0.1% NP-40 alternative, 0.1% Triton X-100, and 0.1% Tween20 and twice with PBS for 10 min under overhead rotation at room temperature. The supernatant was removed and the samples were subjected to on-bead tryptic digestion.

Samples were resuspended in 50 μL of denaturing/reducing buffer (8 M urea, 50 mM Tris-HCl (pH 7.5) and 1 mM DTT) and incubated for 30 min at room temperature. Afterward, samples were alkylated by addition of 5.55 μL of 50 mM 2-chloroacetamide in denaturing/reducing buffer prior to incubation for 30 min at room temperature. Afterward, 1 μg of Lys-C (0.5 μg/μL in ddH_2_O, FUJIFILM Wako Pure Chemical Corporation, JP) was added and samples were incubated for 1 h at 37 °C. The supernatants were transferred to new tubes. 165 μL of 50 mM TRIS-HCl, pH 7.5 containing 1 μg of trypsin (Roche, US) was added and incubated for 1 h at 37 °C. The supernatants from both the Lys-C and trypsin digestion were combined and another 1–2 μg trypsin was added. The digestion was proceeded overnight at 37 °C and the reaction was stopped by addition of 10% (v/v) TFA on the next day. The peptides were desalted by StageTip Purification.

Two layers of EmporeTM High Performance Extraction C18 Disks (3 M Bioanalytical Technologies, US) were stacked on top of each other in a 200 μL pipet tip using a syringe. The filters were activated by addition of 100 μL methanol and centrifugation until all liquid has passed through the disks. The filters were washed and equilibrated once with 100 μL of 0.1% formic acid and twice with 100 μL of 80% acetonitrile, 0.1% formic acid prior to loading of the samples and incubation for 1 min. The solution was removed by centrifugation and the filters were washed with 100 μL of 0.1% formic acid. Twenty μL of 80% acetonitrile, 0.1% formic acid were added and samples were incubated for 1 min prior to elution of the sample by centrifugation at 1,500*g* for 5 min. The previous step was repeated once and the supernatants were combined prior to evaporation using a vacuum concentrator at 30 °C. The dried peptides were dissolved in 20 μL of 0.1% TFA and analyzed by nanoHPLC-MS/MS using an Ultimate 3000 RSLC nano-HPLC system and a Hybrid-Orbitrap mass spectrometer Q Exactive Plus or Q Exactive HF, respectively (Thermo Fisher Scientific, US, different instruments were used for the two biological replicates; technical replicates were analyzed on the same instrument). 1 μL (Q Exactive HF) or 2 μL (Q Exactive Plus) of the peptide solution was injected and enriched on a C18 PepMap 100 column (5 mm, 100 Å, 300 mm ID × 5 mm, Dionex, Thermo Fisher Scientific, US,) using 0.1% TFA, at a flow rate of 30 μL/min, for 5 min and separated on a C18 PepMap 100 column (3 mm, 100 Å, 75 mm ID × 50 cm) using a linear gradient (5–30% ACN/H_2_O + 0.1% formic acid over 90 min) with a flow rate of 300 nL/min. The nano-HPLC apparatus was coupled online with the mass spectrometer using a standard coated PicoTip emitter (ID 20 μm, Tip-ID 10 μM, New Objective, US). Signals in the mass range of *m*/*z* 300 to 1650 were acquired at a resolution of 70,000 (Q Exactive Plus) or 60,000 (Q Exactive HF) for full scan followed by up to 10 (Q Exactive Plus) or 15 (Q Exactive HF) high-energy collision-dissociation (HCD) MS/MS scans of the most intense at least doubly charged ions at a resolution of 17,500 (Q Exactive Plus) or 15,000 (Q Exactive HF), respectively. Proteins were relatively quantified by using MaxQuant^75,76^ v.2.0.3.0, including the Andromeda search algorithm and searching in parallel the *Homo sapiens* reference proteome of the UniProt database and a contaminants database implemented in MaxQuant. Briefly, an MS/MS ion search was performed for enzymatic trypsin cleavage, allowing two missed cleavages. Carbamidomethylation was set as a fixed protein modification, and oxidation of methionine and acetylation of the N-terminus were set as variable modifications. The mass accuracy was set to 20 ppm (ppm) for the first search and to 4.5 ppm for the second search. The false discovery rates for peptide and protein identification were set to 0.01. Only proteins for which at least two peptides were quantified were chosen for further validation. Relative quantification of proteins was performed by using the label-free quantification algorithm implemented in MaxQuant.

Statistical data analysis of pulldown samples was performed separately for both biological replicates (four technical replicates each) using Perseus^77,78^ v.1.6.14.0 including proteins, which were identified in at least three out of four technical replicates in at least one of the two compared conditions. Label-free quantification (LFQ) intensities were log-transformed (log_2_); replicate samples were grouped together. Missing values were imputed using small normally distributed values, and a two-sided *t* test was performed. Volcano plots were generated using the VolcaNoseR^44,79^ web app. Proteins with log_2_-fold changes <1 or >1 and −log_10_(*p*) > 4.5 were considered as statistically significant enriched.

### sEH-H Assay

Inhibition of the C-terminal lipid epoxide hydrolase domain of the soluble epoxide hydrolase (seH-H) was tested using the Soluble Epoxide Hydrolase Inhibitor Screening Assay Kit (cat#10011671, Cayman, US). Briefly, 2.5 μL of 50× diluted sEH protein was treated with compounds at indicated concentrations and the reaction was initiated by addition of 250 nM fluorogenic sEH-H substrate PHOME (3-phenyl-cyano(6-methoxy-2-naphthalenyl)methyl ester-2-oxiraneacetic acid)). The final volume for the assay was 100 μL in all wells. After 10 s, the fluorescence was recorded using an excitation wavelength at 330 nm and an emission wavelength at 465 nm at 25 °C on the Spark Multimode Microplate Reader (Tecan, AT) for up to 30 min in a kinetic loop with intervals of 90 s between measurements. The background was subtracted from all values and all values were normalized to the initial activity of each condition. The IC_50_ value was determined by area-under-curve analysis using GraphPad Prism 9.0 (GraphPad Software, Inc., US).

### sEH-P Assay

Inhibition of the N-terminal lipid phosphatase domain of the soluble epoxide hydrolase (sEH-P) was tested using an AttoPhos-based assay as described previously.^22^ 5 μL of 50× diluted sEH protein (cat#600037, Cayman, US, from the Soluble Epoxide Hydrolase Inhibitor Screening Assay Kit) was incubated with compounds at indicated concentrations in AttoPhos assay buffer (25 mM BIS-TRIS, 1 mM MgCl_2_ x 6 H_2_O, 0.1 mg/mL BSA, pH 7.0) for 5 min at 23 °C prior to addition of 7.5 μL of AttoPhos solution (166.7 μM AttoPhos reagent (cat#S1011 Promega, US) in AttoPhos assay buffer to a final concentration of 25 μM. The samples were incubated for 60 min at 23 °C in the dark under mild shaking. Afterward, 25 μL of AttoPhos stop solution (100 mM NaOH in AttoPhos buffer) was added and the fluorescence was recorded using an excitation wavelength at 435 nm and an emission wavelength at 555 nm on the Spark Multimode Microplate Reader (Tecan, AT) for up to 30 min in a kinetic loop with intervals of 90 s between measurements. The final volume for the assay was 75 μL in all wells. The analysis was performed using the data from 15 min after the stop solution was added, i.e., when the fluorescence signal became stable. Therefore, all values were subtracted by the background and normalized to the DMSO control.

### nanoDSF

Purified sEH protein (cat#600037, Cayman, US, from the Soluble Epoxide Hydrolase Inhibitor Screening Assay Kit) was diluted 18× in AttoPhos assay buffer and incubated with compounds at indicated concentrations for 10 min at 22 °C. The thermal protein stability from 20 to 90 °C (1 °C/min) was measured by means of the intrinsic tryptophan/tyrosine fluorescence using high sensitivity capillaries in the Prometheus NT.48 (NanoTemper Technologies, DE). Melting scans, first derivatives of melting scans, and melting temperatures were analyzed using the PR.ThermControl software (NanoTemper Technologies, DE).

### In-Cell CETSA

Jurkat cells (10 × 10^6^ cells/flask) were seeded in two T25 tissue culture flasks and treated with 10 μM compound or DMSO for 20 min at 37 °C. Cells were harvested and washed thrice in ice-cold PBS. Compound- and DMSO-treated samples were distributed equally into 10 tubes each and subjected to heating at different temperatures ranging from 37 to 72.1 °C (37 °C, 51.5 °C, 55.2 °C, 58.1 °C, 61.9 °C, 63.8 °C, 65.7 °C, 67.6 °C, 69.2 °C, 72.1 °C) in the Mastercycler X50s (Eppendorf SE, DE). Afterward, cOmplete EDTA-free Protease Inhibitor Cocktail (Roche, CH) and NP-40 alternative, sodium chloride, and Tris-HCl, pH 8.0 (final concentrations of 0.4% (v/v), 150 and 50 mM, respectively) were added and cells were lysed by three consecutive freeze/thaw cycles. Soluble fractions were separated from denatured proteins by centrifugation at 20,000*g* and 4 °C for 25 min. Supernatants were transferred to new tubes and subjected to immunoblot analysis.

### sEH NanoBRET

Cellular target engagement was studied with the NanoBRET technology using a full-length sEH-NanoLuc construct and the fluorescent sEH-H ligand **4** as published previously.^49^ HEK293T cells (5 × 10^5^ cells/well) were seeded in 6-well plates containing 2 mL growth medium and incubated for 24 h. Afterward, the medium was removed and the cells were washed with PBS prior to transfection with sEH-aa1-aa555_C-NanoLuc_pF32Kp^49^ plasmid (2.5 μg/well) using 3.75 μL of Lipofectamine 3000 and 5 μL of P3000 reagent (Invitrogen, US) in Opti-MEM according to the manufacturer’s protocol. The cells were incubated for 4 h and subsequently, the transfection mix was replaced by growth medium and the cells were incubated for additional 20 h. On the next day, the cells were washed thrice with PBS and harvested in Opti-MEM. The cell titer was determined and the concentration was adjusted to 5 × 10^4^ cells/mL.

4.25 × 10^4^ cells, 60 nM of the fluorescent sEH-H tracer **4**, and epoxykynin at indicated concentrations were mixed in a total volume of 20 μL in a white 384-well plate. The plate was sealed with an AeraSeal film (Sigma-Aldrich, US) and incubated at 37 °C and 5% CO_2_ for 5 h. 9 μL of NanoGlo substrate and 3 μL of extracellular NanoLuc inhibitor (Intracellular TE Nano-Glo Kit, Promega, US) were diluted in Opti-MEM with a total volume of 1.5 mL to yield the NanoGlo mixture. Afterward, 10 μL of the NanoGlo mixture was added to each well prior to short centrifugation and measurement of the BRET signal. First, the donor luminescence was monitored at 458 nm with a bandwidth of 25 nm for 500 ms on the Spark Multimode Microplate Reader (Tecan, AT). Subsequently, the acceptor fluorescence was measured at 578 nm with a bandwidth of 25 nm for 500 ms. The BRET ratio was determined by multiplying the acceptor signal by 1000 and dividing by the donor signal. After plotting of the BRET ratio against the logarithmic compound concentration, data analysis was performed using a nonlinear regression curve fit in GraphPad Prism 9.0 (GraphPad Software, Inc., US) to generate dose–response curves and obtain IC_50_ values.

### Co-crystallization of Epoxykynin and Human sEH-H

The C-terminal hydrolase domain of human sEH (sEH-H, aa222-aa555) was cloned and expressed in *E. coli* as described previously.^80−82^ Briefly, sEH-H protein was expressed in *E. coli* BL21(DE3) using ZYP5052 autoinduction medium^83^ at 16 °C for 36 h. sEH-H was purified by nickel affinity chromatography in 50 mM TRIS-HCl, pH 8.0, 500 mM NaCl, 70 mM imidazole-HCl and eluted in 50 mM TRIS-HCl, pH 8.0, 500 mM NaCl, 400 mM imidazole-HCl followed by size-exclusion chromatography using a HiLoad 16/60 Superdex 200 column (GE Healthcare, US) in 50 mM NaCl, 50 mM sodium phosphate, 10% (v/v) glycerol (98%), 2 mM DTT, pH 7.4. The pure protein was concentrated with an Amicon Ultra-15 (10 kDa cutoff).

440 μM sEH-H (16.7 mg/mL) was mixed with 1 mM epoxykynin for 1 h on ice. Initial crystallization trials were performed using commercially available screens. A drop volume of 200 nL was equilibrated against 20 μL of reservoir solution in 96-well sitting drop plates (SWISSCI, UK). The crystallization plates were incubated at 20 °C. The crystals were obtained in 25% PEG Smear High, 0.1 M PIPES, pH 7.0, 0.1 M magnesium formate, 0.1 M rubidium chloride.

A single crystal was picked from the crystallization plate, treated with 25% ethylene glycol in reservoir solution as the cryoprotectant, and frozen in liquid nitrogen for each complex. Synchrotron X-ray diffraction data was acquired from the X06SA beamline at the Swiss Light Source at the Paul Scherrer Institute, CH. The temperature was maintained at 100 K during the data collection. A total of 900 images were collected, and the data were processed using XDS.^84^ The intensities were scaled and converted into structure factors using AIMLESS^85,86^ of the CCP4 suite.^87^ The structure was solved by molecular replacement using the MOLREP^88^ program of the CCP4 suite with the published sEH-H structure from PDB 7P4K(89) as the search template. The model was built manually using COOT^90^ and the structure was refined by REFMAC of the CCP4 suite. The model was validated using the wwPDB Validation System (https://validate-rcsb-2.wwpdb.org/) prior to deposition. The data collection, processing, and refinement statistics are shown in Table S6. The coordinate was deposited in the Protein Data Bank with the PDB ID 8QZD.

### Knockdown Experiments

HeLa cells (150,000 cells/well) were seeded in 6-well plates in medium without phenol red and incubated for 24 h. On the next day, cells were treated with 50 ng/mL IFN-γ and 164.15 μM Trp and transfected with siRNA. The siRNAs (ON-TARGETplus Human EPHX2 siRNA SMARTPool (cat# L-010006–00–0005, Horizon Discovery, UK) and ON-TARGETplus Nontargeting Control siRNAs (cat# D-001810–01–05, Horizon Discovery, UK)) were diluted to 2 μM in Opti-MEM in a total volume of 50 μL (final siRNA concentration: 30 nM to 100 nM). The DharmaFECT 1 reagent was diluted in Opti-MEM in a total volume of 50 μL (siRNA to transfection reagent ratio of 1 to 15). Both siRNA and transfection reagent were incubated for 5 min at room temperature prior to addition of the DharmaFECT solution to the siRNA solution. The transfection mixture was incubated for another 20 min at room temperature to allow formation of the siRNA:lipid complex. Subsequently, 1.9 mL of culture medium was added to the transfection mixture and the culture medium on the cells was replaced by the transfection mixture. The cells were incubated for up to 96 h. To quantify Kyn levels, 100 μL of the supernatant was transferred to a transparent 96-well plate and Kyn levels were determined with *p*-DMAB. The remaining supernatant was aspirated and RNA was extracted for qPCR analysis as described above or cells were lysed for immunoblotting. Therefore, the cells were resuspended in 500 μL of NP-40-based lysis buffer (150 mM sodium chloride, 0.4% NP-40 alternative, 50 mM TRIS-HCl, pH 8.0), incubated on ice for 30 min, and centrifuged for 25 min at 20,000*g* and 4 °C. The supernatant was subjected to immunoblotting.

### Overexpression Experiments

HeLa cells (50,000 to 100,000 cells/well) were seeded in a 12-well plate in medium without phenol red and incubated for 24–48 h. Subsequently, cells were treated with 50 ng/mL IFN-γ and 164.15 μM Trp and transfected with 55 to 438 ng/well pCMV3-EPHX2 (Sino Biological, CN) using Lipofectamine 3000 (2 μL transfection reagent per μg plasmid DNA, Invitrogen, US) prior to incubation for 24–48 h. To quantify Kyn levels, 100 μL of the supernatant was transferred to a transparent 96-well plate and Kyn levels were determined with *p*-DMAB. The remaining supernatant was aspirated and the cells were resuspended in 500 μL of NP-40-based lysis buffer (150 mM sodium chloride, 0.4% NP-40 alternative, 50 mM TRIS-HCl, pH 8.0), incubated on ice for 30 min and centrifuged for 25 min at 20,000*g* and 4 °C. The supernatant was subjected to immunoblotting.

## Supporting Information Available

Additional experimental details and spectra, supporting figures, raw data, and experimental replicates (PDF)
SMILES of all compounds; screening data (CSV)

## ABBREVIATIONS

Kyn: kynurenine
IDO1: indoleamine 2,3-dioxygenase 1
IFN-γ: interferon-γ
sEH: soluble epoxide hydrolase
