# Supplementary material for: Discovery of the sEH Inhibitor Epoxykynin as a Potent Kynurenine Pathway Modulator
Source: J Med Chem. 2024 Mar 12;67(6):4691–706. doi: 10.1021/acs.jmedchem.3c02245 (PMC10983002; doi:10.1021/acs.jmedchem.3c02245)
Supplement: Supplementary file 1 — jm3c02245_si_001.pdf [file jm3c02245_si_001.pdf]

Supporting Information

Discovery of the sEH Inhibitor Epoxykynin as Potent Kynurenine Pathway Modulator

Lara Dötsch,[1,2] Caitlin Davies†,[1] Elisabeth Hennes,[1] Julia Schönfeld,[4] Adarsh Kumar,[4,5] Celine Da Cruz Lopes Guita,[1] Johanna H.M. Ehrler,[4] Kerstin Hiesinger,[4] Sasikala Thavam,[1] Petra Janning,[1] Sonja Sievers,[3] Stefan Knapp,[4,5] Ewgenij Proschak,[4] Slava Ziegler,[1] Herbert Waldmann*[1,2]

[1] L. Dötsch, C. Davies, E. Hennes, C. Da Cruz Lopes Guita, S. Thavam, P. Janning, S. Ziegler, H. Waldmann
Max Planck Institute of Molecular Physiology, Department of Chemical Biology
Otto-Hahn-Strasse 11, 44227 Dortmund (Germany)
[2] L. Dötsch, H. Waldmann
Technical University of Dortmund, Department of Chemical Biology
Otto-Hahn-Strasse 6, 44227 Dortmund (Germany)
[3] S. Sievers
Compound Management and Screening Center (COMAS)
Otto-Hahn-Strasse 15, 44227 Dortmund (Germany)
[4] J. Schönfeld, A. Kumar, J.H.M. Ehrler, K. Hiesinger, S. Knapp, E. Proschak
Goethe University Frankfurt, Institute of Pharmaceutical Chemistry
Max-von-Laue-Strasse 9, 60438 Frankfurt (Germany)
[5] A. Kumar, S. Knapp
Structural Genomics Consortium, Buchmann Institute for Molecular Life Sciences, Goethe University Frankfurt
Max-von-Laue-Strasse 15, 60438 Frankfurt (Germany)

Corresponding Author:

*Herbert Waldmann
herbert.waldmann@mpi-dortmund.mpg.de

Contents of SI

1. Supplementary Data S2
   1.1 Supporting Tables S2
   1.2 Supporting Figures S18
2. Chemical Synthesis S33
   2.1 NMR Spectra S33
   2.2 HPLC Spectra S37
3. Supplementary References S41

1. Supplementary Data

1.1 Supporting Tables

Table S1. Structure-activity relationship (SAR) for reduction of Kyn levels (related to Table 1). IC_{50} values and Kyn assay inhibitions were determined in BxPC-3 cells using the automated Kyn assay. IC_{50} values are mean values (mean values ± SD, n≥3) and Kyn assay inhibition values were determined as a single point measurement at 7.1 µM. Table is continued on following pages.

entry	Kyn assay IC_{50} [µM]	Kyn assay inhibition [%]	entry	Kyn assay IC_{50} [µM]	Kyn assay inhibition [%]
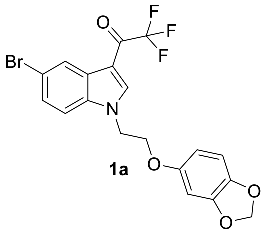 1a	0.09 ± 0.03	95	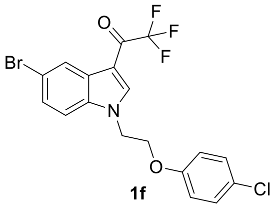 1f	0.05 ± 0.02	98
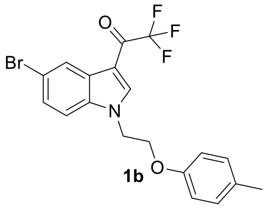 1b	0.10 ± 0.02	83	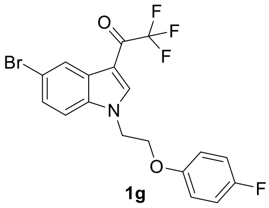 1g	3.05 ± 0.25	100
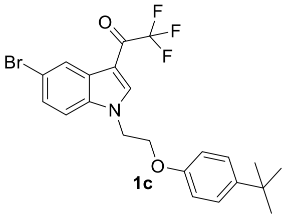 1c	3.01 ± 0.80	20	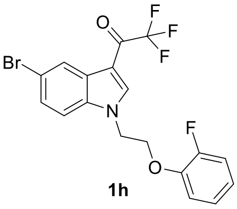 1h	0.10 ± 0.03	99
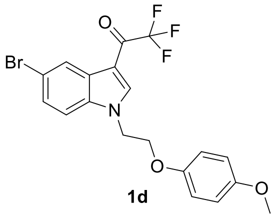 1d	>10	12	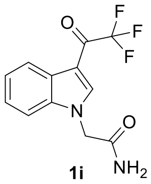 1i	0.05 ± 0.01	112
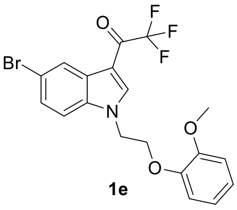 1e	5.43 ± 0.6	54	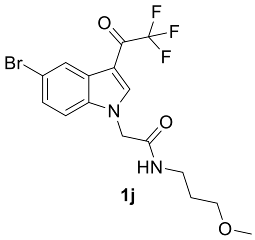 1j	0.40 ± 0.10	95

entry	Kyn assay IC_{50} [µM]	Kyn assay inhibition [%]	entry	Kyn assay IC_{50} [µM]	Kyn assay inhibition [%]
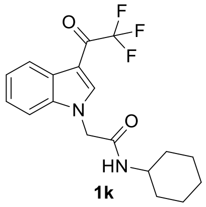 **1k**	3.49 ± 0.30	41	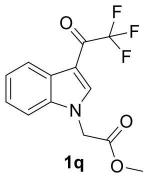 **1q**	>10	-7
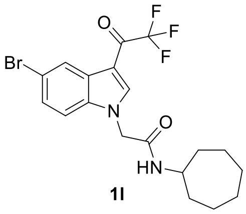 **1l**	0.04 ± 0.02	98	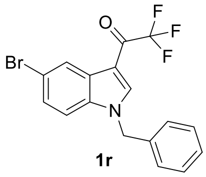 **1r**	>10	12
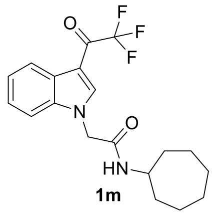 **1m**	1.73 ± 0.10	55	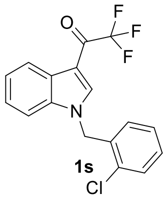 **1s**	>10	11
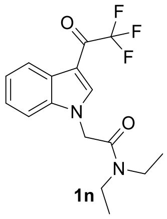 **1n**	>10	0	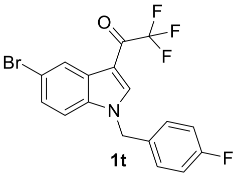 **1t**	0.77 ± 0.41	67
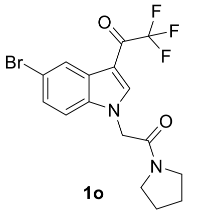 **1o**	1.94 ± 0.20	70	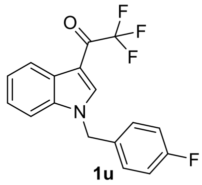 **1u**	>10	28
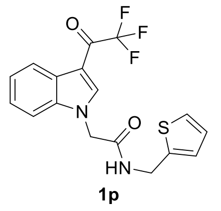 **1p**	7.24 ± 3.80	49	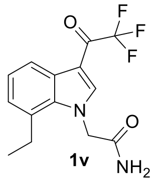 **1v**	>10	2

entry	Kyn assay IC_{50} [µM]	Kyn assay inhibition [%]	entry	Kyn assay IC_{50} [µM]	Kyn assay inhibition [%]
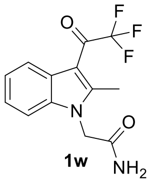 1w	>10	-36	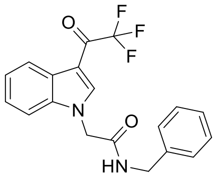	>10	3
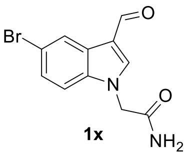 1x	>10	-3	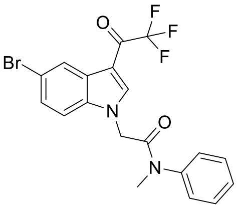	6.65 ± 2.6	12
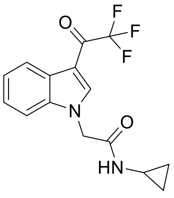	>10	45	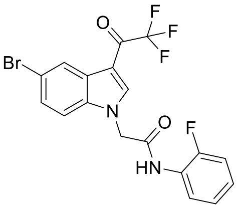	2.27 ± 0.6	52
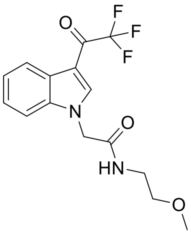	>10	0	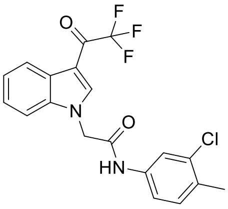	0.753 ± 0.4	85
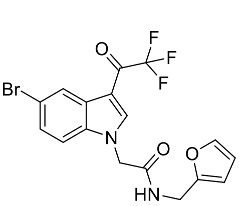	0.564 ± 0.12	93	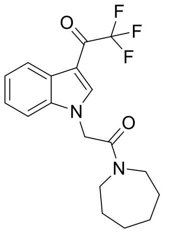	>10	16
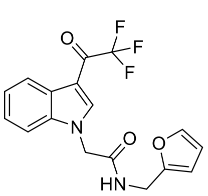	9.12 ± 1.1	34	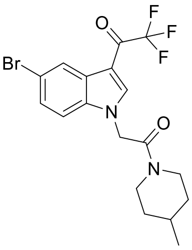	0.343 ± 0.04	96

entry	Kyn assay IC50 [µM]	Kyn assay inhibition [%]	entry	Kyn assay IC50 [µM]	Kyn assay inhibition [%]
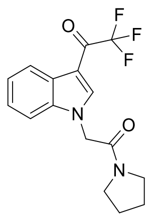	>10	-23	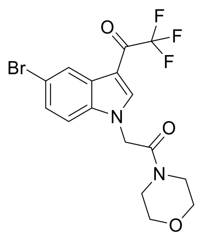	>10	-8
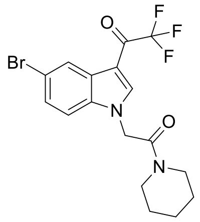	1.43 ± 0.2	88	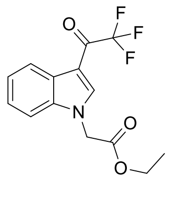	>10	-9
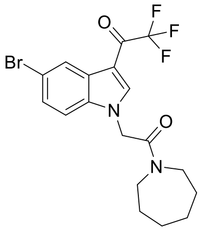	1.23 ± 0.2	88	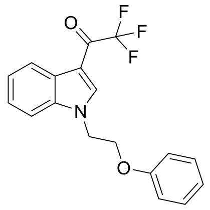	1.18 ± 0.7	84
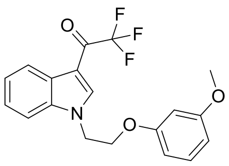	>10	-11	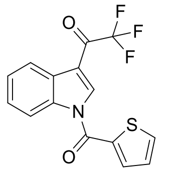	not determined	1
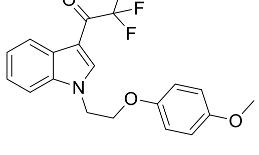	>10	18	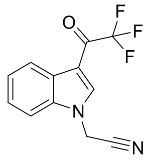	>10	10
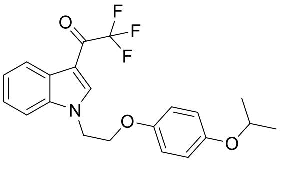	0.493 ± 0.12	96	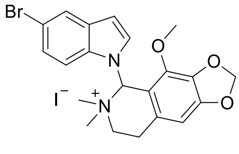	not determined	3

entry	Kyn assay IC50 [µM]	Kyn assay inhibition [%]	entry	Kyn assay IC50 [µM]	Kyn assay inhibition [%]
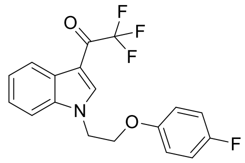	2.05 ± 0.1	108	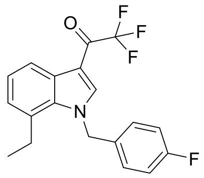	>10	-4
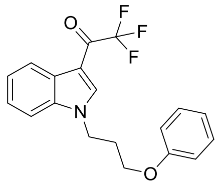	>10	10	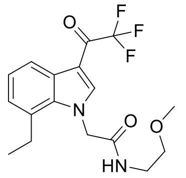	>10	-9
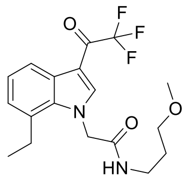	>10	-23	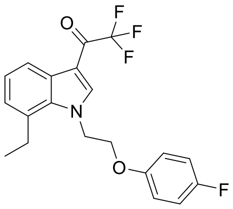	1.12 ± 0.4	77
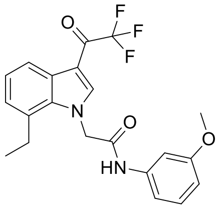	>10	-13	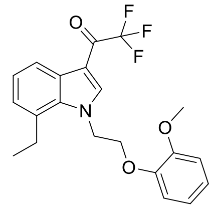	>10	47
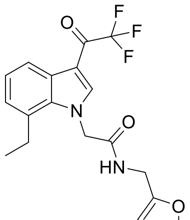	>10	37	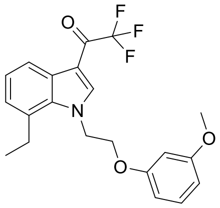	3.89 ± 2.4	74
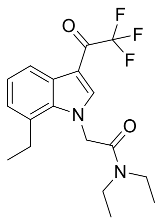	>10	4	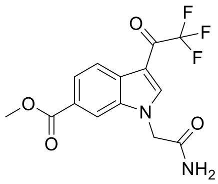	>10	-14

entry	Kyn assay IC_{50} [µM]	Kyn assay inhibition [%]	entry	Kyn assay IC_{50} [µM]	Kyn assay inhibition [%]
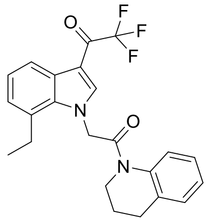	>10	23	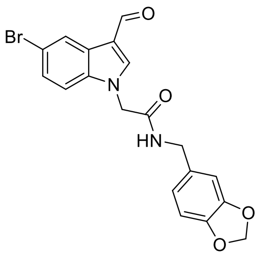	not determined	-19
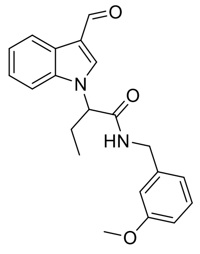	1.99 ± 0.4	71	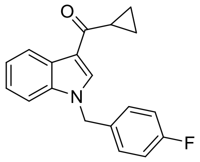	7.18 ± 1.4	59
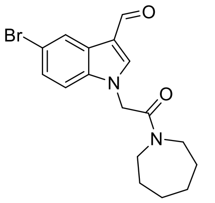	not determined	-22	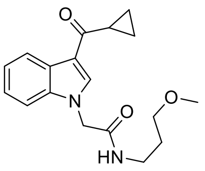	>10	-7
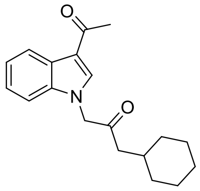	>10	-11	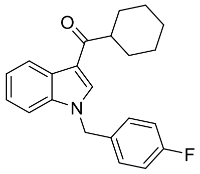	3.94 ± 0.7	21
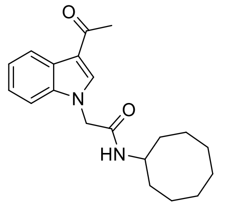	>10	-18	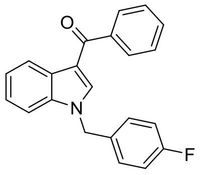	>10	0
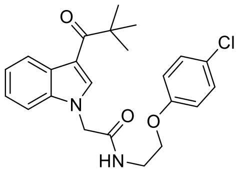	2.14 ± 0.1	70	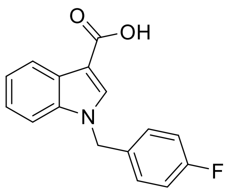	>10	7

entry	Kyn assay IC_{50} [µM]	Kyn assay inhibition [%]	entry	Kyn assay IC_{50} [µM]	Kyn assay inhibition [%]
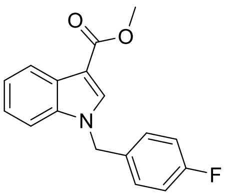	9.32 ± 1.0	42	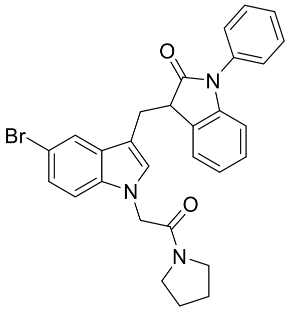	not determined	36
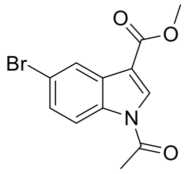	not determined	-18	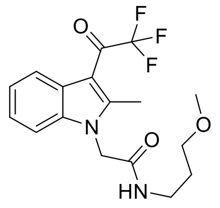	>10	-2
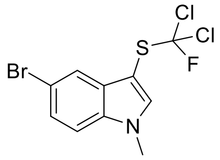	not determined	42	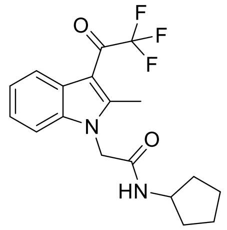	4.29 ± 1.6	63
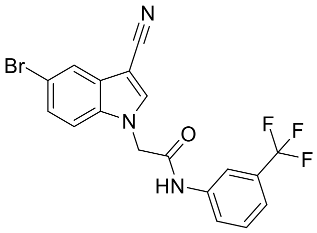	not determined	-30	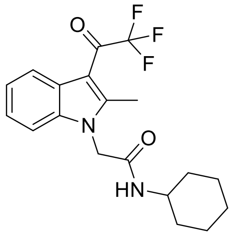	4.4 ± 0.4	12
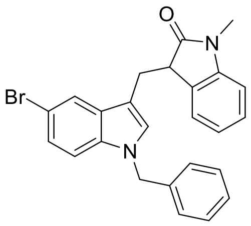	not determined	23	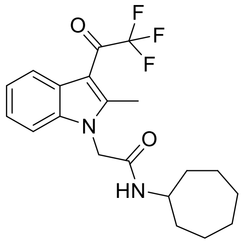	1.7 ± 0.1	82
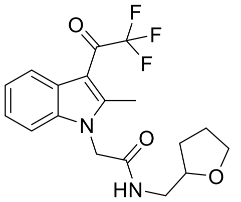	>10	1	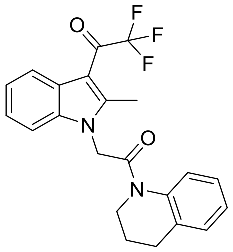	>10	-15

entry	Kyn assay IC_{50} [µM]	Kyn assay inhibition [%]	entry	Kyn assay IC_{50} [µM]	Kyn assay inhibition [%]
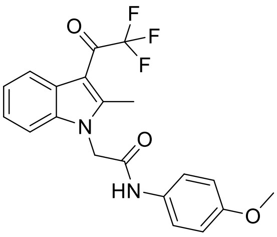	>10	-2	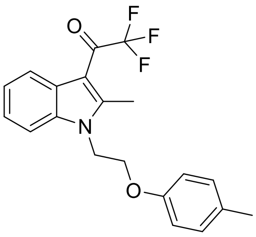	>10	-22
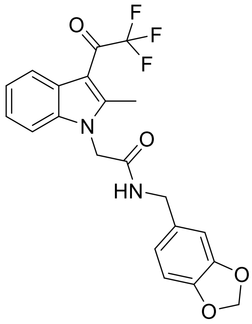	>10	-4	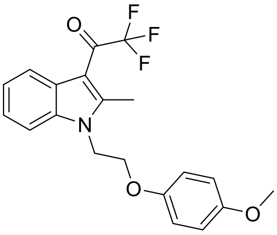	0.275 ± 0.08	97
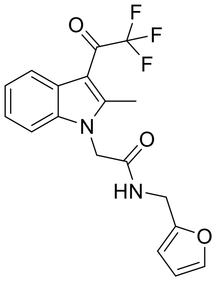	>10	23	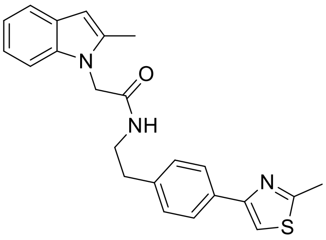	1.6 ± 0.0	87
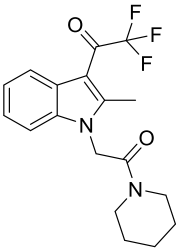	>10	-1	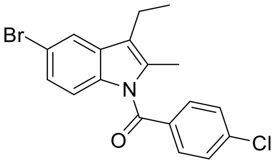	not determined	-7
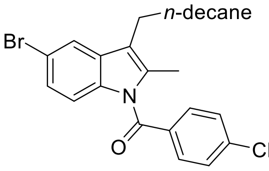	not determined	2	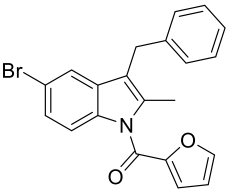	>10	42
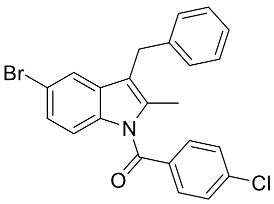	not determined	12	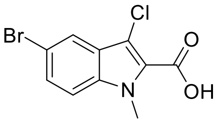	not determined	4

entry	Kyn assay IC$_{50}$ [µM]	Kyn assay inhibition [%]	entry	Kyn assay IC$_{50}$ [µM]	Kyn assay inhibition [%]
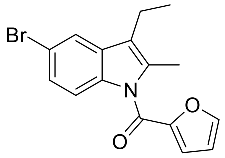	not determined	18	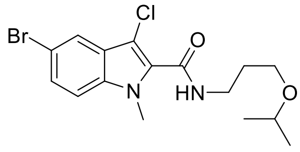	not determined	-4

Table S2. Affinity-based chemical proteomics (pulldown). Enriched proteins on affinity probe **2b** as identified by HRMS (n=2, N=4, FDR 0.01). Proteins that were identified in both experimental replicates are highlighted in grey. Related to Figure 2.

	n1, N=4		n2, N=4	
**gene name**	**significance** (-log(*p*))	**difference** (log_2(2b-3b))	**significance** (-log(*p*))	**difference** (log_2(2b-3b))
EPHX2	2.14	1.99	4.74	2.54
HIBCH			4.47	1.11
HPCAL1;HPCA			3.54	1.63
LYPLA2			4.32	1.14
MTAP	6.47	2.34	4.65	1.04
RPL31			1.88	1.46
SEH1L	5.77	2.38	8.38	3.31
TRA2B	4.44	2.36		

Table S3. Affinity-based chemical proteomics (pulldown). Enriched proteins on control probe **3b** as identified by HRMS (n=2, N=4, FDR 0.01). Proteins that were identified in both experimental replicates are highlighted in grey. Table is continued on the next pages. Related to Figure 2.

	n1, N=4		n2, N=4	
**gene name**	**significance** (-log(*p*))	**difference** (log_2(2b-3b))	**significance** (-log(*p*))	**difference** (log_2(2b-3b))
ABCB7			3.99	-1.10
ADK	3.63	-2.53	8.27	-2.29
AKAP8			1.95	-4.07
ALG5			2.87	-1.30
APOO			5.01	-1.54
ARL6IP5			4.54	-1.40
ARL8B;ARL8A			3.03	-1.47
ATL3			6.25	-1.26
ATP1B3			2.16	-1.22
ATP5EP2;ATP5E			1.06	-2.06
ATP5F1			4.90	-1.08
ATP5J	1.99	-2.04		
ATP5J2;ATP5J2-PTCD1			4.92	-1.08
ATP5L			4.75	-1.05
BCAP31			4.09	-1.71
BET1;DKFZp781C0425			1.61	-1.71
BST2			4.99	-1.48
C1GALT1			4.11	-1.56
CCDC134			1.98	-1.64
CD44			5.52	-1.38
CD97			4.32	-1.08
CDC42			3.03	-1.19
CERS2			1.23	-1.78
CHCHD3			4.86	-1.25
CISD1			5.84	-1.59
CISD2			5.47	-1.79
CLPTM1L			5.41	-1.34
COMT			4.56	-1.18
COX4I1			4.13	-1.46
COX5A			4.64	-1.11
CSE1L			5.70	-1.04
CYB5R3			4.03	-1.14
DAD1			2.98	-1.72
DEGS1			3.87	-1.27
DHCR7			5.03	-1.17
DHRS1			5.12	-1.39
DHRS3			1.78	-1.73
EDIL3			4.63	-1.13
ELOVL1			1.52	-1.73
ELOVL5			4.25	-1.24
EPHX1			5.28	-1.33
ERGIC1			5.45	-1.18
FADS1			6.51	-1.37
FADS2			5.42	-1.08
FAM105A			3.82	-1.31
FKBP11	2.29	-2.35	3.88	-1.09
FUNDC2	3.94	-2.88	3.25	-2.34
GALNT2			4.70	-1.22
GLUD1; GLUD2	5.49	-1.67		
GNB2			4.58	-1.47
GNG5			3.32	-2.39
GPR89A;GPR89B			4.26	-1.52
GPX8	1.74	-2.91	5.58	-1.27
HCCS			4.07	-1.51
HEATR1			4.34	-1.08
HEBP1	5.95	-2.58	7.07	-1.71

	n1, N=4		n2, N=4	
**gene name**	**significance** (-log(*p*))	**difference** (log_2(2b-3b))	**significance** (-log(*p*))	**difference** (log_2(2b-3b))
HLA-A			5.18	-1.12
HLA-B			5.85	-1.21
HLA-C;HLA-Cw			6.13	-1.09
HMOX2			2.90	-1.19
IKBIP			3.59	-1.09
IMMT			5.80	-1.54
ITPA	7.11	-2.85		
LCLAT1			4.33	-1.19
LMAN1			5.19	-1.07
LMAN2			4.80	-1.93
LMAN2L			3.30	-1.41
LMF2			5.73	-1.19
MAGT1			3.07	-1.27
MBOAT7			3.91	-1.07
MCU			4.73	-1.13
MGST1			7.32	-1.55
MLEC			2.87	-1.49
MPC1;BRP44L			3.22	-1.41
MPC2			2.09	-2.13
MPDU1			5.77	-1.38
MRRF	1.72	-2.19		
MT-ATP8			3.10	-1.76
MTCH2			4.98	-1.12
MT-CO2			5.61	-1.26
MTOR			4.15	-1.45
MTX1			3.19	-1.68
NDC1			5.47	-1.10
NDUFA8			1.71	-2.00
NDUFAF2			3.64	-1.78
NDUFB11			3.56	-2.54
NDUFB3			2.91	-1.38
NDUFB5			2.58	-1.33
NT5C3B			4.26	-2.45
PDCD6			4.93	-1.04
PFDN6	2.68	-2.03		
PGM3			1.60	-1.89
PGRMC1			2.03	-1.66
PGRMC2	5.25	-2.35	5.06	-1.35
PIGS			4.47	-1.31
PIGT			3.43	-1.68
PITPNB	3.07	-1.85		
POR			2.96	-1.15
PRKDC			6.33	-1.04
PSMD8	1.60	-2.76		
PTGES2	2.46	-1.91	4.52	-1.10
PTRH2			3.44	-2.13
RAB10			5.76	-1.54
RAB11B;RAB11A			4.73	-1.57
RAB14			4.70	-1.27
RAB18			4.35	-2.48
RAB1A			2.32	-1.98
RAB1B			5.56	-1.10
RAB21			6.51	-1.78
RAB2A			4.94	-1.55
RAB31;RAB22A			4.19	-1.76
RAB5A			1.75	-1.80
RAB5C			5.12	-1.35
RAB6A; RAB6B	3.54	-1.75		
RAB7A			5.39	-1.45
RAB8A			4.36	-1.10

	n1, N=4		n2, N=4	
**gene name**	**significance** (-log(*p*))	**difference** (log_2(2b-3b))	**significance** (-log(*p*))	**difference** (log_2(2b-3b))
RAP1B; RAP1A	5.15	-3.86	4.61	-1.80
RDH11			5.07	-1.16
RETSAT			2.60	-1.31
RFT1			3.56	-1.83
RHOA			1.44	-1.98
RHOG			3.93	-2.42
S100A10			2.24	-2.67
SACM1L			4.85	-1.26
SAMM50			6.98	-1.74
SAR1A			2.86	-1.74
SCAMP3	4.90	-2.82		
SCARB1			4.86	-2.93
SCCPDH	3.90	-1.94	2.87	-1.17
SCPEP1	3.14	-2.01		
SEC11A			6.78	-1.37
SEC22B			4.69	-1.43
SEC61B			6.41	-1.59
SEC61G			5.67	-1.83
SEC63			1.62	-2.21
SECTM1			1.95	-2.08
SIGMAR1			2.04	-2.18
SLC30A7			2.68	-1.65
SMPD4			2.01	-1.41
SOAT1			4.68	-1.17
SPCS2			4.77	-1.24
SPCS3			4.57	-1.17
SPR			6.49	-1.58
SSR3			1.19	-1.65
STT3A			5.09	-1.01
SYPL1			2.75	-1.36
TAMM41			2.34	-1.24
TAPBP			3.64	-1.41
TBL2	4.26	-1.49	4.86	-1.15
TM9SF1			3.10	-1.19
TM9SF2			5.99	-1.03
TM9SF3			4.62	-1.12
TM9SF4			2.22	-1.71
TMED2			3.30	-1.38
TMED7-TICAM2;TMED7			4.25	-1.18
TMEM109			1.98	-1.97
TMEM205			1.66	-2.06
TMPO			4.49	-1.16
TOMM20			1.36	-2.28
TOMM7			4.28	-1.26
TOR1AIP1			3.54	-1.08
TPP1			1.83	-1.41
TSPO			4.84	-1.35
TUBGCP4			2.45	-1.20
VAPA			4.92	-1.51
VAPB			2.70	-1.28
VDAC1			5.11	-1.31
VMP1			1.88	-1.51
XPO7			6.26	-1.04
YIF1A			3.68	-1.24
ZMPSTE24			5.52	-1.69

Table S4. Affinity-based chemical proteomics (pulldown). LFQ intensities for proteins selectively enriched on affinity probe **2b** in comparison to control probe **3b** as determined by HRMS (n=2, N=4, FDR 0.01).

	gene name	affinity probe 2b				control probe 3b			
		LFQ1	LFQ2	LFQ3	LFQ4	LFQ1	LFQ2	LFQ3	LFQ4
n1	EPHX2	7,352,600	6,270,200	6,809,100	6,688,400	0	0	0	0
	MTAP	13,678,000	16,810,000	18,212,000	16,267,000	3,253,500	3,132,500	2,994,100	3,442,400
	SEH1L	75,267,000	88,492,000	108,110,000	80,662,000	15,245,000	18,745,000	16,230,000	17,053,000
n2	EPHX2	23,157,000	15787000	15518000	21115000	0	0	0	0
	MTAP	17,151,000	20,828,000	17,404,000	18,929,000	9,893,700	8,366,300	8,462,400	9,450,700
	SEH1L	281,169,984	254,340,000	262,070,000	260,070,000	24,289,000	25,395,000	28,607,000	28,273,000

Table S5. Structure-activity relationship (SAR) for reduction of Kyn levels (related toTable S1) and sEH-H inhibition. Kyn assay IC_{50} values (mean values ± SD, n≥3) and Kyn assay inhibitions were determined in BxPC-3 cells using the automated Kyn assay. Inhibition of human sEH-H was measured by means of the conversion of the fluorogenic sEH-H substrate PHOME (mean values ± SD, n=3).

entry	Kyn assay IC_{50}	Kyn assay inhibition]	sEH-H assay IC_{50}	sEH-H assay inhibition
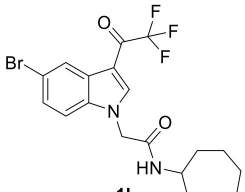 **1l** (epoxykynin)	36.0 ± 15.0 nM	98%	6.7 ± 3.2 nM	84%
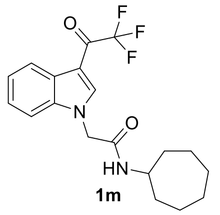 **1m**	1.7 ± 0.1 µM	55%	50.1 ± 11.2 nM	82%
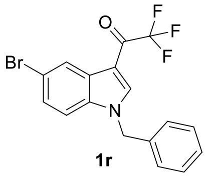 **1r**	>10 µM	12%	>10 µM	n.d.
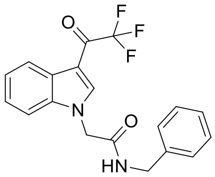	>10 µM	3%	619.6 ± 71.2 nM	44%

Table S6. Data collection, processing, and refinement statistics for the sEH-H-epoxykynin complex (pdb 8qzd).

	**8QZD**
**Data collection and reduction**	
Wavelength (Å)	1.0
Space group	$P22_12_1$
Resolution range (Å)	44.54 - 1.30
Last resolution shell (Å)	1.33 - 1.30
Unit cell parameters	
a,b,c (Å)	45.92, 80.31, 89.08
α, β, γ (°)	90, 90, 90
Total number of observations	518234 (21137)
Unique reflections	80739 (3673)
Mosaicity (˚)	0.20
Multiplicity	6.4 (5.8)
Mean I/σ(I)	10.7 (1.5)
Completeness (%)	99.6 (93.3)
R_{merge}^{b}	0.072 (1.071)
R_{meas}^{c}	0.085 (1.298)
R_{pim}^{d}	0.045 (0.723)
**Refinement**	
Resolution range (Å)	44.54 - 1.30
Number of reflections used	76583
Number of Free R flagged reflections	4091
R_{cryst}^{e}	0.16279
R_{free}^{f}	0.18041
rmsd Bond length (Å)	0.012
rmsd Bond angle (˚)	1.926
Ramachandran plot, residues in	
Most favored region (%)	98.2
Additionally allowed region (%)	1.5
Average B-factor ($Å^2$)	18.140

[a]Values for the last resolution shell are in parentheses.

[b]$R_{merge} = \Sigma_{hkl}\Sigma_i|I_i(hkl)-\langle I(hkl)\rangle| / \Sigma_{hkl}\Sigma_i I_i(hkl)$, where I(hkl) is the intensity of reflection hkl

[c]$R_{meas} = \Sigma_{hkl}(n/(n-1))^{1/2}\Sigma_i|I_i(hkl)-\langle I(hkl)\rangle| / \Sigma_{hkl}\Sigma_i I_i(hkl)$

[d]$R_{pim} = \Sigma_{hkl}(1/(n-1))^{1/2}\Sigma_i|I_i(hkl)-\langle I(hkl)\rangle| / \Sigma_{hkl}\Sigma_i I_i(hkl)$

[e]$R_{cryst} = \Sigma_{hkl} ||F_{obs}|-|F_{calc}|| / \Sigma|F_{obs}|$

[f]R_{free} is the cross-validation R-factor computed for the test set of unique reflections.

1.2 Supporting Figures

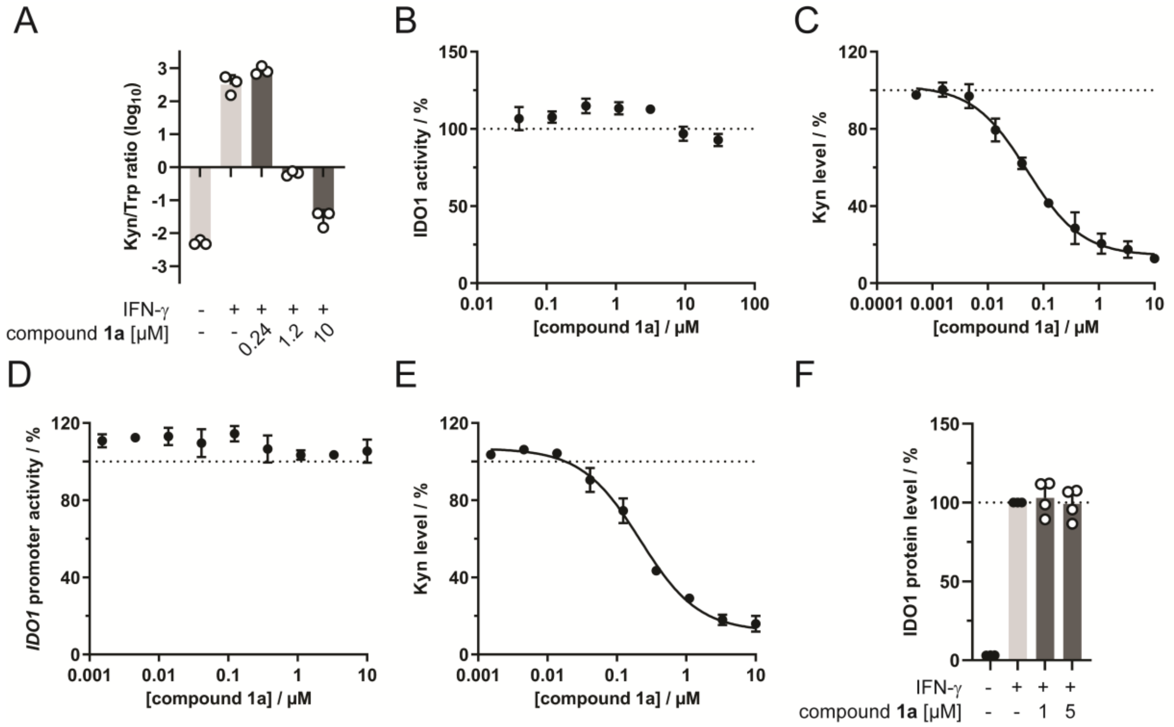

Figure S1. Reduction of cellular Kyn levels by initial hit compound (**1a**) and influence on IDO1 expression. A) Kyn/Trp ratio in BxPC-3 cells upon treatment with compound **1a**. Cells were treated with IFN-γ, Trp and compound **1a** prior to quantification of Trp and Kyn levels by LC-MS (mean values ± SD, n=3). B) *In vitro* IDO1 enzymatic activity. Purified IDO1 was treated with compound **1a** or DMSO for 40 min at 37°C prior to addition of Trp and incubation for 60 min at 37°C. Kyn levels were detected using p-DMAB (mean values ± SD, n=4). C) Kyn assay in IFN-γ-HeLa cells. Cells were treated with IFN-γ, Trp and compound **1a** for 48 h prior to measuring Kyn levels using *para*-dimethylaminobenzaldehyde (*p*-DMAB, mean values ± SD, n=3). IC_{50} = 55.4 ± 17.4 nM. D) *IDO1* promoter-dependent reporter gene assay in HEK293T cells expressing firefly luciferase (Fluc) under the control of the *IDO1* promoter and constitutive *Renilla* luciferase expression (Rluc). Cells were treated with IFN-γ to induce Fluc expression and simultaneously with compound **1a** for 48 h. Fluc values were normalized to the Rluc signal (mean values ± SD, n=3). E) Kyn assay in HEK293T cells transiently expressing human IDO1. Cells were treated with Trp and epoxykynin for 24 h prior to measuring Kyn levels with *p*-DMAB (mean values ± SD, n=3). IC_{50} = 214.8 ± 55.7 nM. F) IDO1 protein levels in HeLa cells that were treated with IFN-γ and compound **1a** or DMSO for 24 h prior to quantification of protein levels via immunoblotting (mean values ± SD, n=4). See also Figure S2 for complete blots. The dotted lines indicate signals of the respective DMSO controls that were set to 100%.

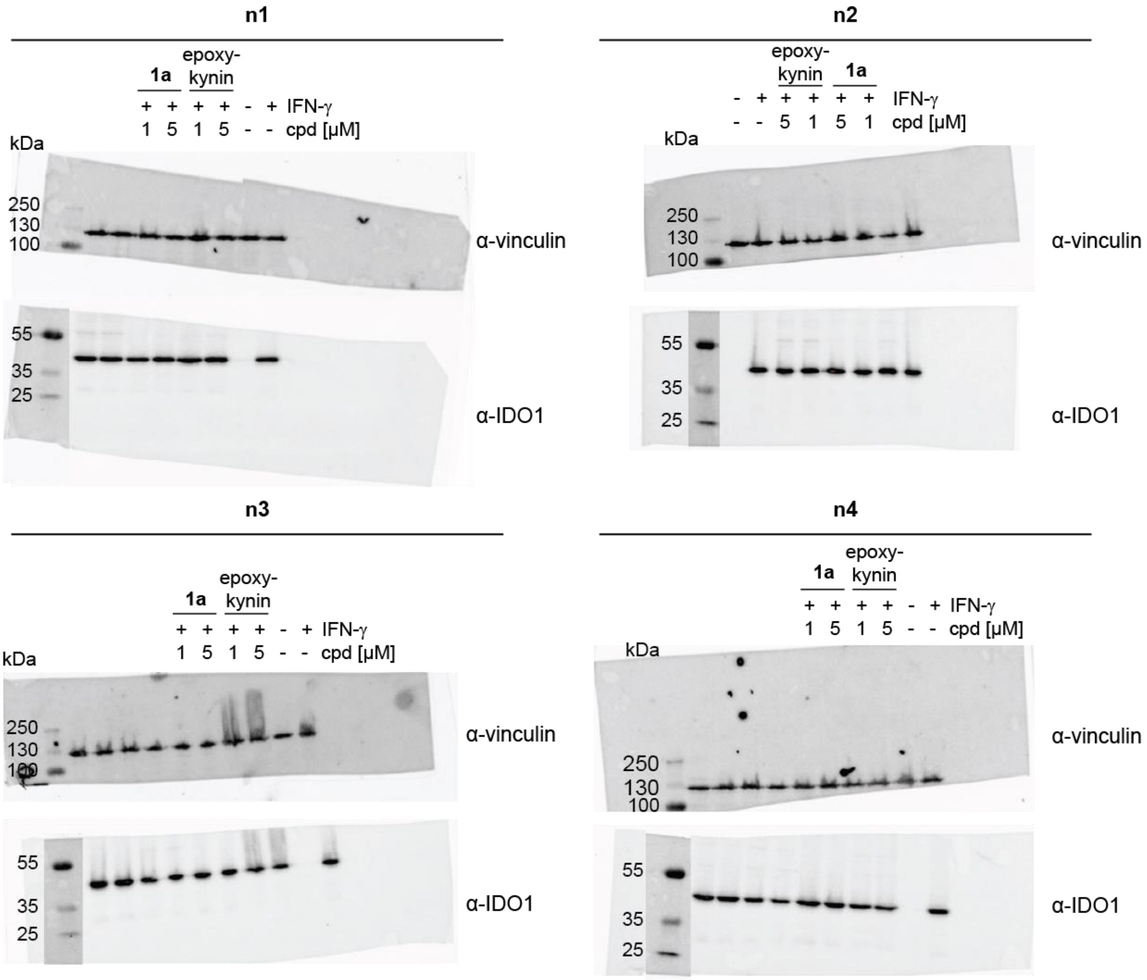

Figure S2. IDO1 protein levels in HeLa cells that were treated with IFN-γ and compound **1a** and epoxykynin or DMSO for 24 h prior to quantification of protein levels via immunoblotting. IDO1 protein bands were monitored in the IRDye800CW channel, the vinculin control bands and marker bands were monitored in the IRDye680RD channel. Related to Figure 1H.

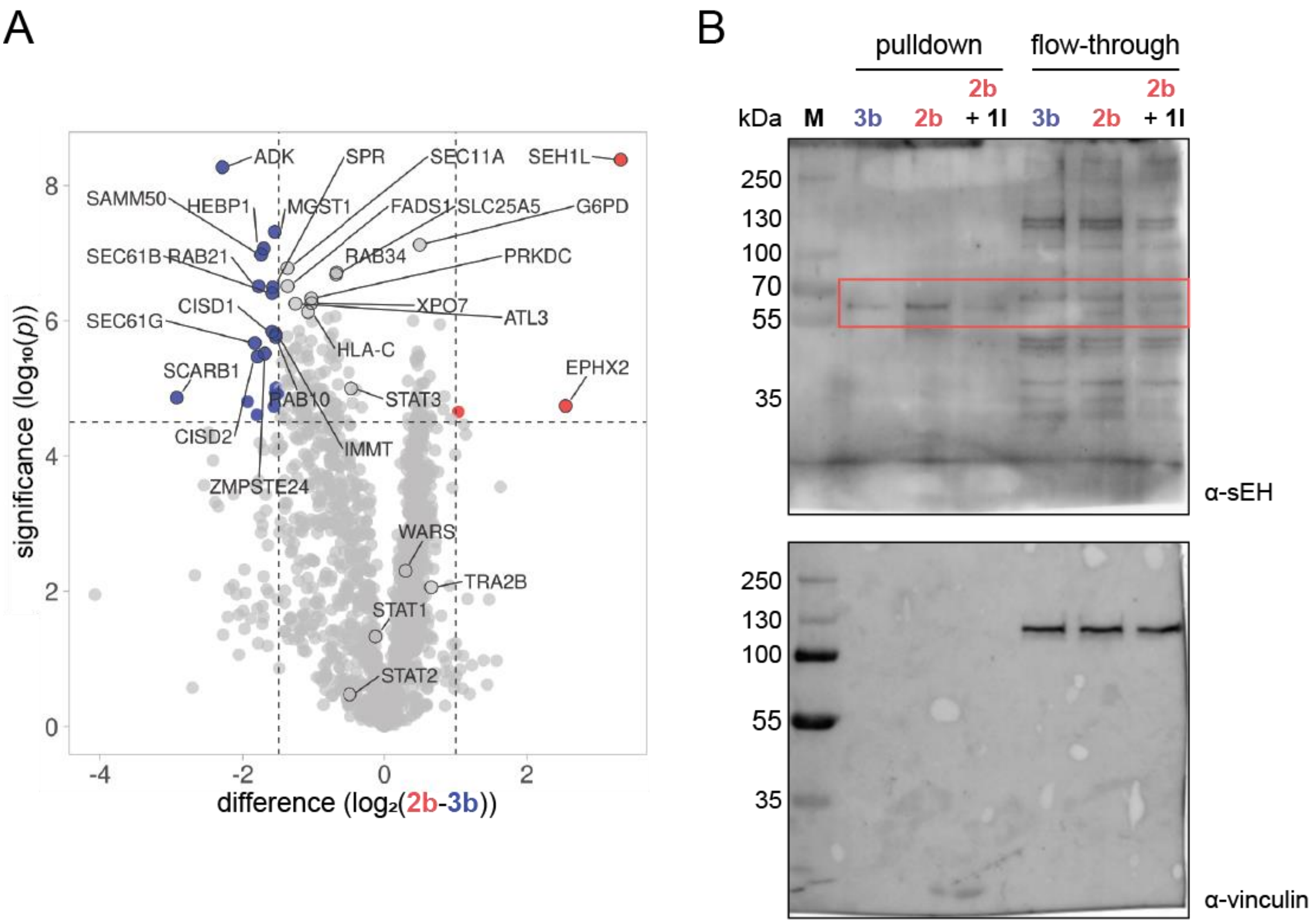

Figure S3. Affinity-based chemical proteomics (pulldown). A) The affinity probes **2b** and **3b** were immobilized on NHS-activated beads and incubated with HeLa cell lysate for 2 h at 4°C. Enriched proteins were analyzed using HRMS (n=2, N=4, FDR 0.01), representative replicate is shown, see also Table S2 and S3. Volcano plot for proteins enriched by probe **2b** (red) or probe **3b** (blue) created with VolcaNoseR.[1] B) Selective enrichment of EPHX2 (sEH) using the probe **2b** and competition with compound **1l** (epoxykynin). For the competition experiment, HeLa cell lysate was pre-incubated with 10 µM compound **1l** (epoxykynin) for 1 h at 4°C prior to incubation with the immobilized probes **2b** and **3b**. The enriched proteins were analyzed using immunoblot. sEH bands were monitored using an HRP-conjugated secondary antibody, the vinculin and marker (M) signal were monitored using an IRDye680RD secondary antibody. Related to Figure 2.

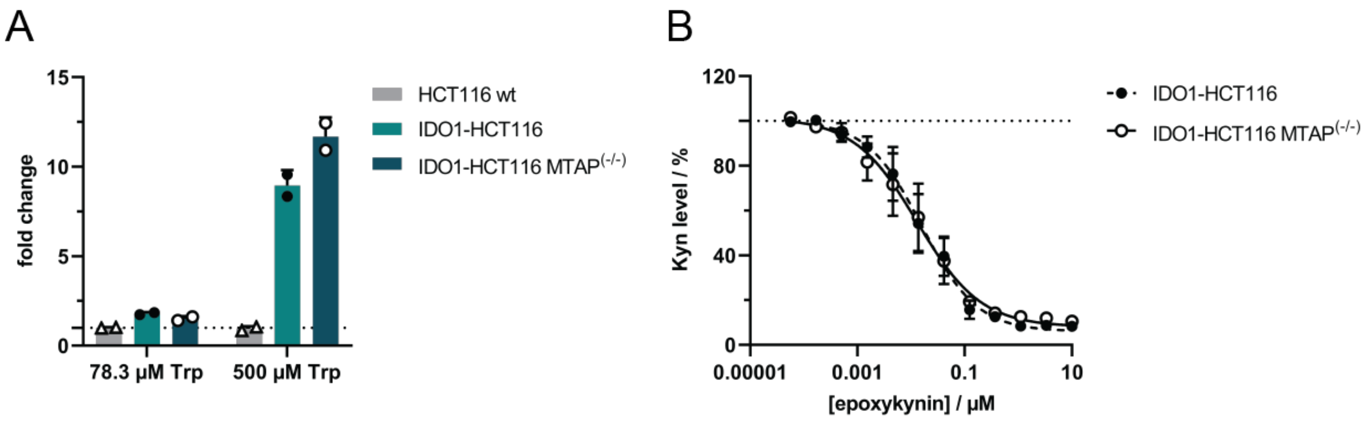

Figure S4. Devalidation of MTAP as target of epoxykynin using HCT116 MTAP$^{(-/-)}$ cells that transiently express IDO1. A) HCT116 wildtype (wt) and HCT116 MTAP (-/-) cells were transiently transfected with IDO1 expression construct for 24 h prior to addition of no (78.3 µM Trp already included in the medium) or 500 µM Trp for 36 h. Kyn levels were quantified using *p*-DMAB (mean values±SD, n=2). B) Kyn assay in HCT116 and HCT116 MTAP (-/-) cells that transiently express IDO1. Cells were transfected with IDO1 for 24 h prior to addition of 500 µM Trp and epoxykynin for 48 h. Kyn levels were quantified using *p*-DMAB (mean values ± SD, n=3). IC_{50}(IDO1-HCT116) = 20.7 ± 11.5 nM; IC_{50}(IDO1-HCT116 MTAP$^{(-/-)}$) = 20.5 ± 15.9 nM.

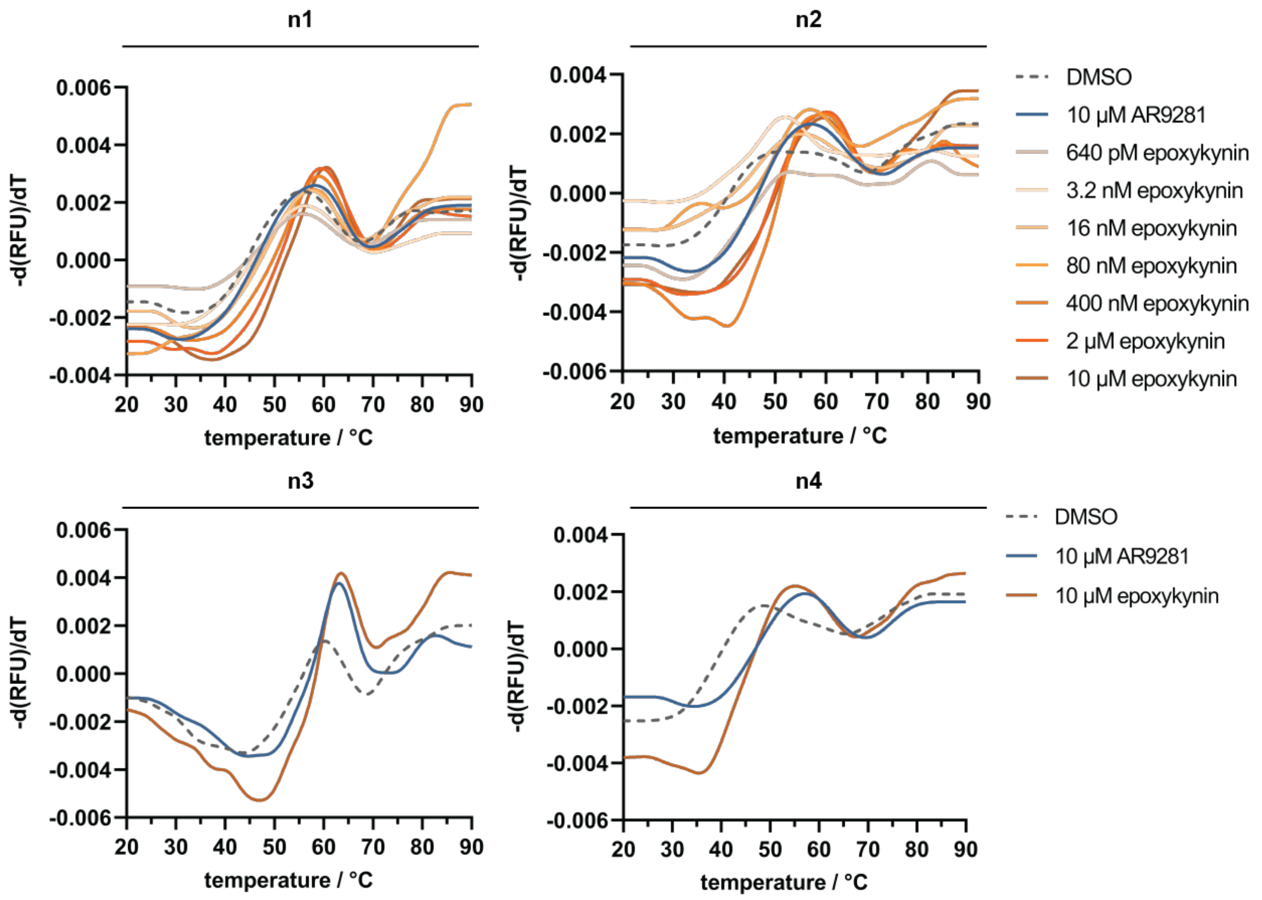

Figure S5. Dose-dependent T_m shift of sEH upon binding of epoxykynin as detected using nanoDSF. Purified sEH was treated with epoxykynin or DMSO for 10 min at room temperature prior to detection of the intrinsic tryptophan/tyrosine fluorescence upon thermal denaturation. Related to Figure 3A.

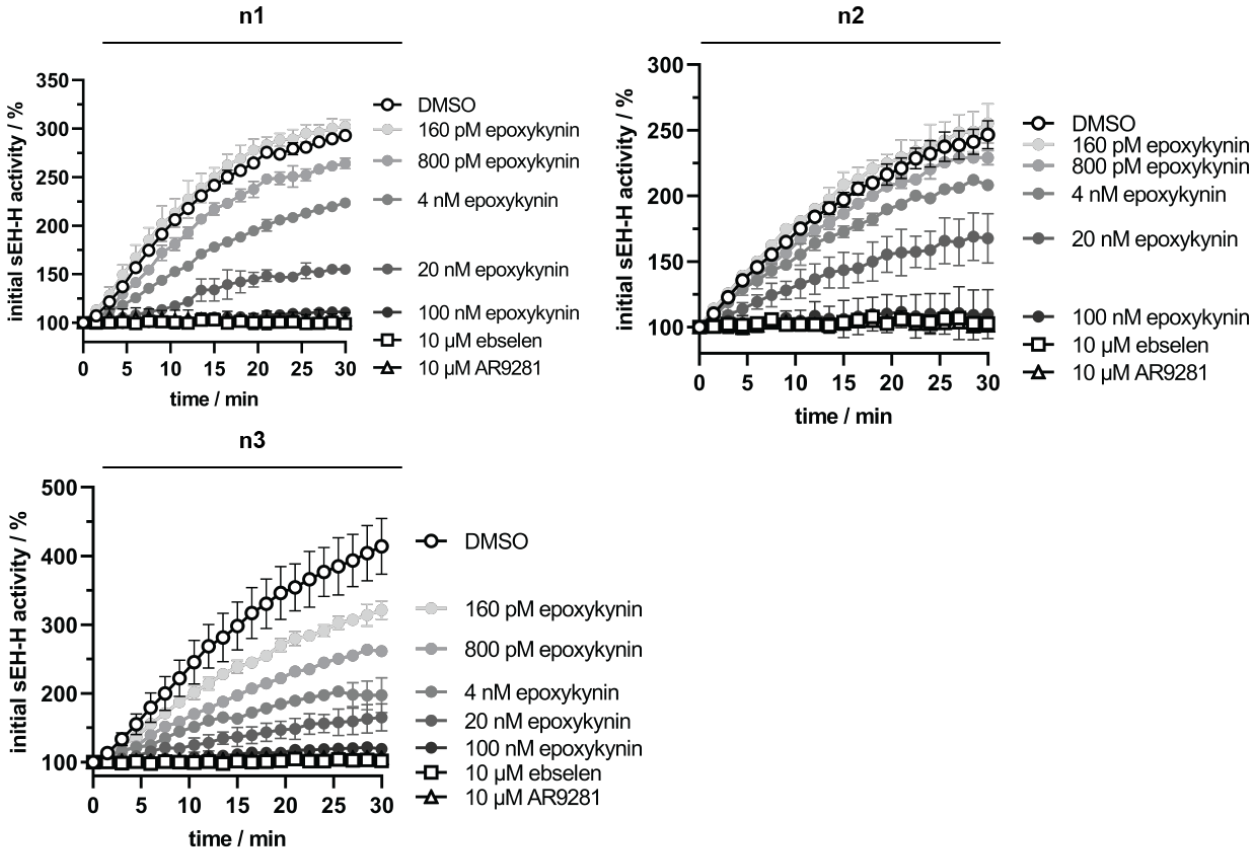

Figure S6. Dose-dependent inhibition of sEH-H by epoxykynin. The epoxide hydrolase activity of purified sEH (sEH-H) was measured by means of the conversion of the fluorogenic sEH-H substrate PHOME upon treatment with epoxykynin, 10 µM ebselen and 10 µM AR9281. Related to Figure 3B.

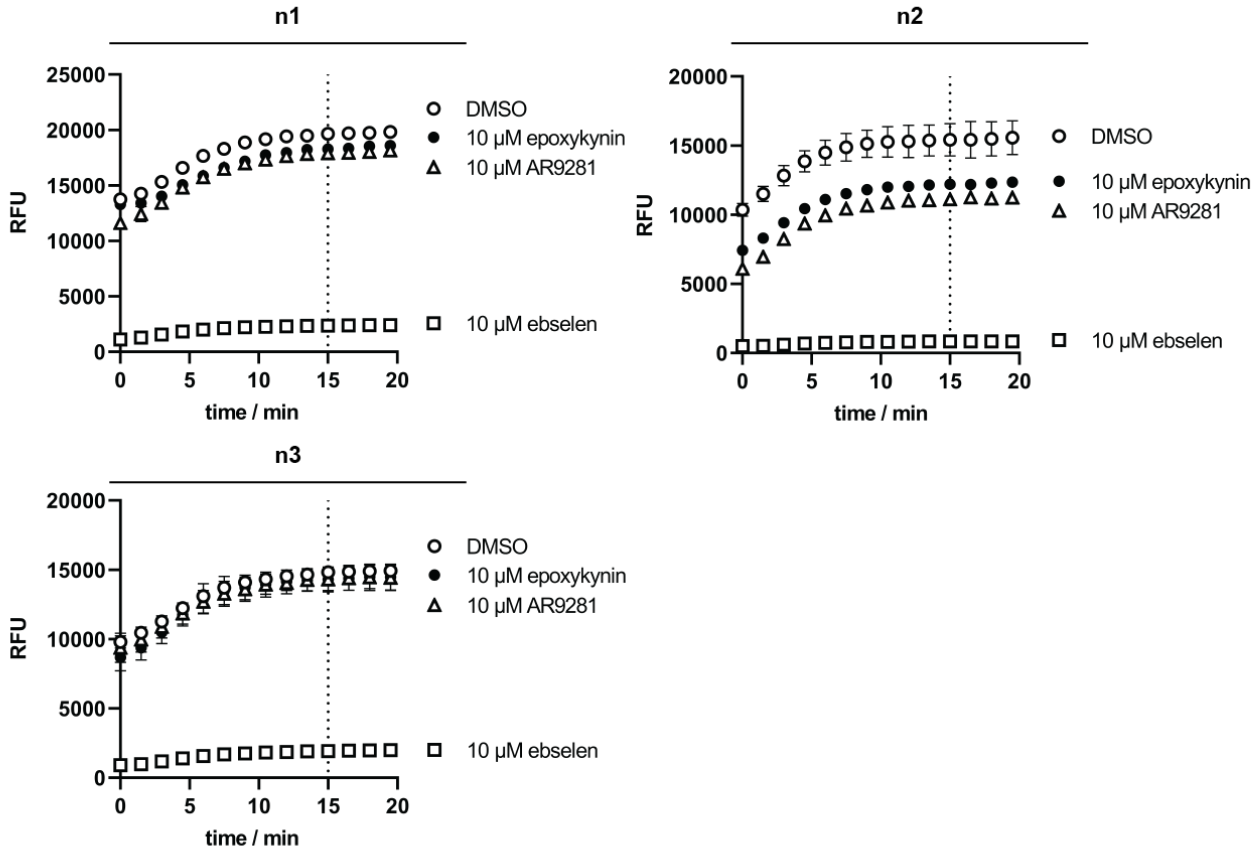

Figure S7. The phosphatase activity of purified sEH (sEH-P) was measured by means of an AttoPhos-based assay upon treatment with epoxykynin or AR9281 and ebselen as controls. Related to Figure 3C.

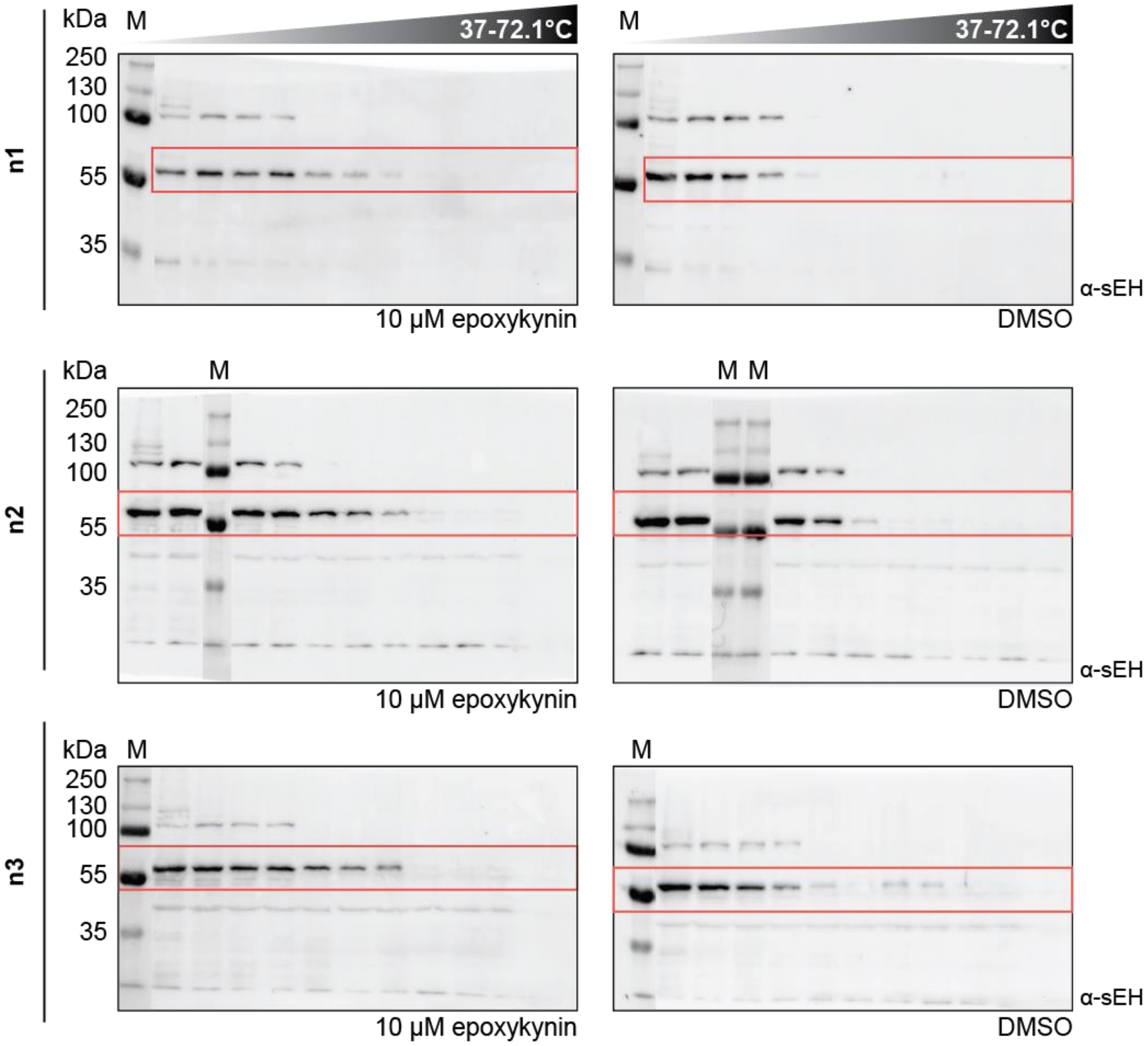

Figure S8. Cellular thermal shift assay (CETSA) for sEH in Jurkat cells. Cells were treated with 10 µM epoxykynin or DMSO for 15 min prior to heat treatment and cell lysis. Soluble proteins were analyzed using immunoblotting. sEH bands were acquired using an IRDye800CW secondary antibody, the marker (M) signal was acquired in the IRDye680RD channel. Related to Figure 3D.

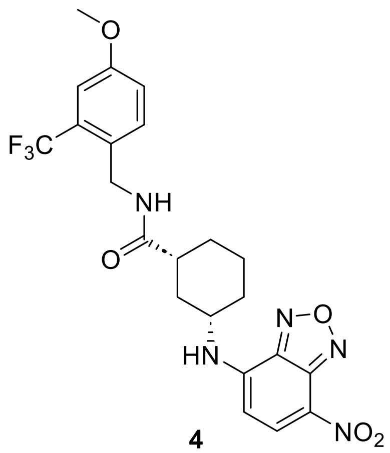

4

Figure S9: Structure of fluorescent tracer **4**[2] used for the sEH-H nanoBRET.

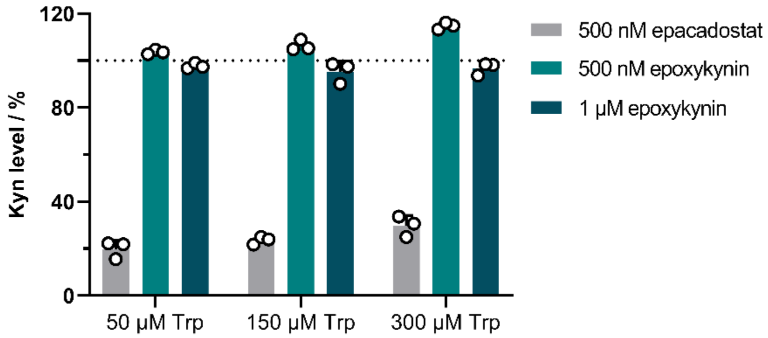

Figure S10. Kyn assay in IFN-γ-HAP1 wt cells. HAP1 cells were treated with epacadostat or epoxykynin, Trp and IFN-γ for 48 h. Kyn levels were quantified using *p*-DMAB (mean values ± SD, n=3).

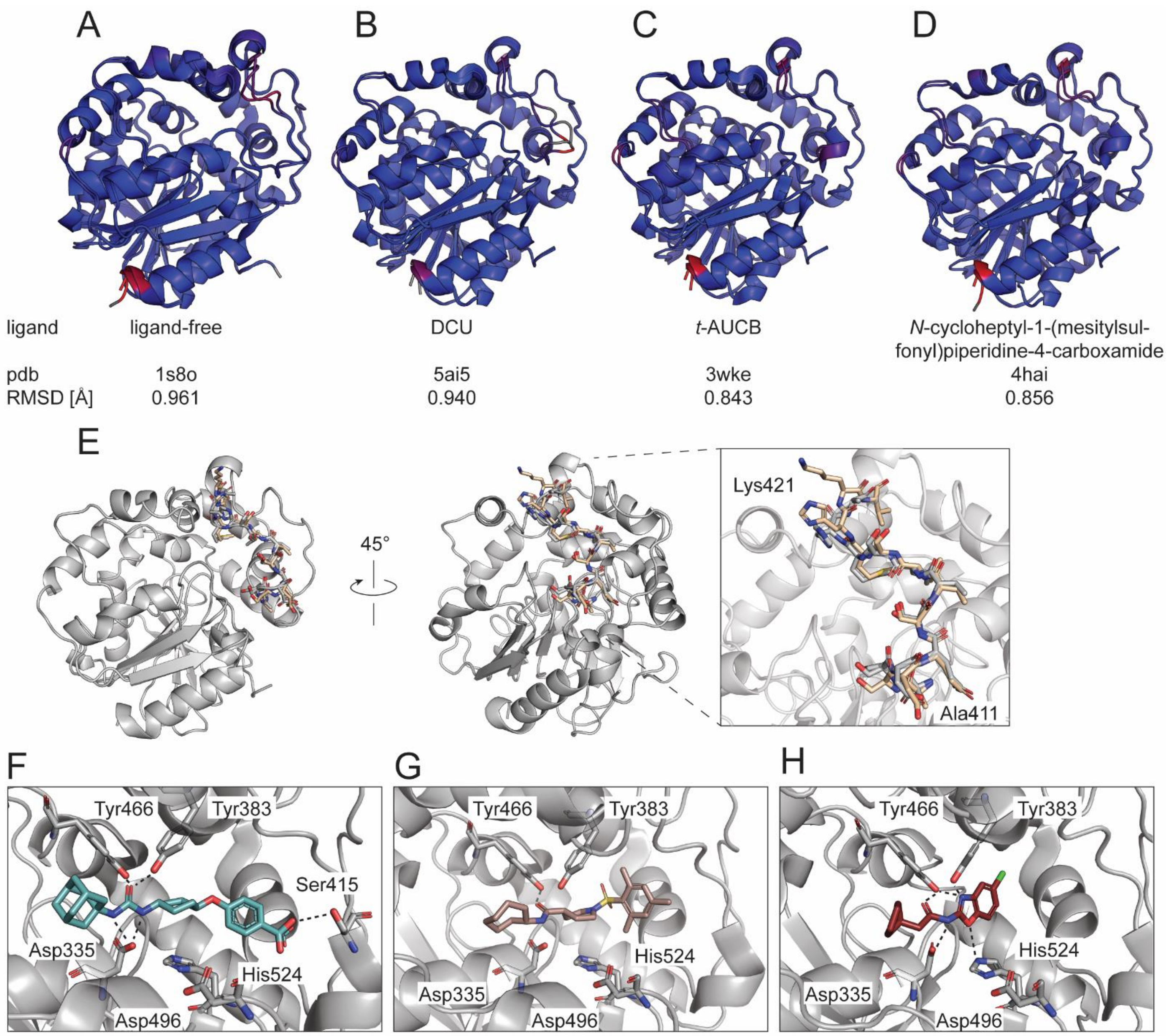

Figure S11. Alignments of co-crystal structures of epoxykynin bound to human sEH-H (pdb 8qzd) with ligand-free sEH-H (A, pdb 1s8o), *N*,*N*'-dicyclourea- (B, DCU, pdb 5ai5), *trans*-4-(4-(3-adamantan-1-ylureido)cyclohexyloxy)benzoic acid- (C, *t*-AUCB, pdb 3wke) and *N*-cycloheptyl-1-(mesitylsulfonyl)piperidine-4-carboxamide-bound sEH-H (D, pdb 4hai). Alignments were performed by superposition of the protein structures. The average distance between the atoms is represented by the root mean square deviation (RMSD). Blue color represents good alignment; red color represents high deviation; grey color represents unaligned residues. E) Overlay of co-crystal structures of epoxykynin (grey, pdb 8qzd) and *t*-AUCB (wheat, pdb 3wke) bound to human sEH-H. Residues Ala411 to Lys421 of the cap domain of sEH-H are shown as sticks. The amino acids in the active site are labeled with the three-letter code. Heteroatoms of the amino acid side chains are depicted in red (oxygen), blue (nitrogen) and yellow (sulfur). F-H) Crystal structures of *t*-AUCB (F, teal sticks, pdb wke), *N*-cycloheptyl-1-(mesitylsulfonyl)piperidine-4-carboxamide (G, brown sticks, pdb 4hai) and *N*-(5-chloro-1,3-benzoxazol-2-yl)-2-cyclopentylacetamide (H, red sticks, pdb 3pdc) bound to human sEH. Polar interactions are indicated by the dotted black lines. The amino acids in the active site are labeled with the three-letter code. Heteroatoms of the ligands and amino acid side chains are depicted in red (oxygen), blue (nitrogen), yellow (sulfur) and green (chlorine). Amino acids 497-500 are omitted for clarity.

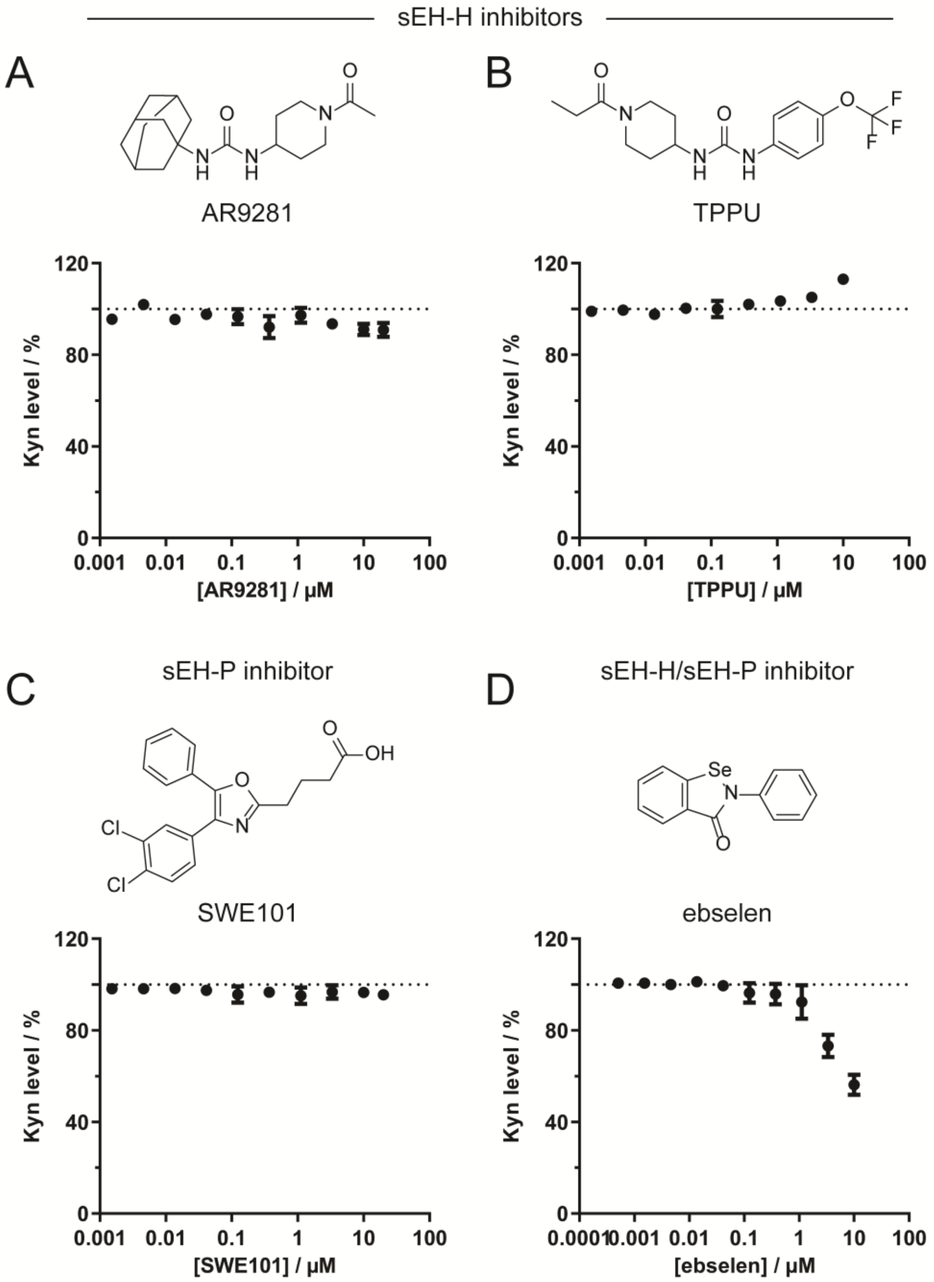

Figure S12: Kyn assay for sEH inhibitors in IFN-γ-HeLa cells. Cells were treated with IFN-γ, Trp and compounds for 48 h prior to measuring Kyn levels using *para*-dimethylaminobenzaldehyde (*p*-DMAB, mean values ± SD, n=3).

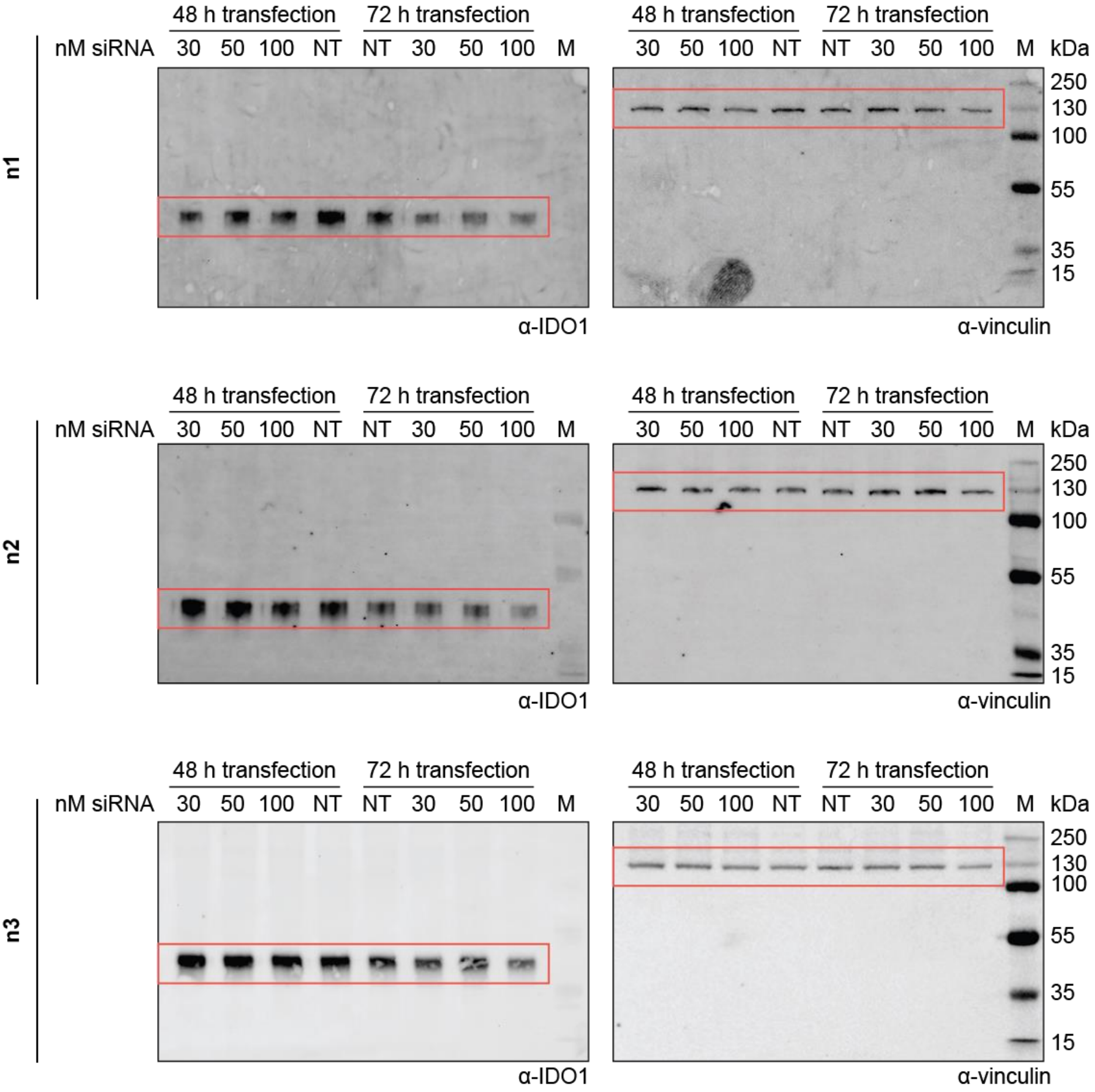

Figure S13. Knockdown (KD) of sEH resulted in decreased IDO1 levels. HeLa cells were transfected with non-targeting (NT) or sEH siRNA for 48-72 h and treated with IFN-γ for 48 h prior to quantification of protein levels *via* immunoblotting. IDO1 protein bands were acquired in the IRDye800CW channel, the vinculin control and marker (M) bands were acquired in the IRDye680RD channel. Related to figure 4A.

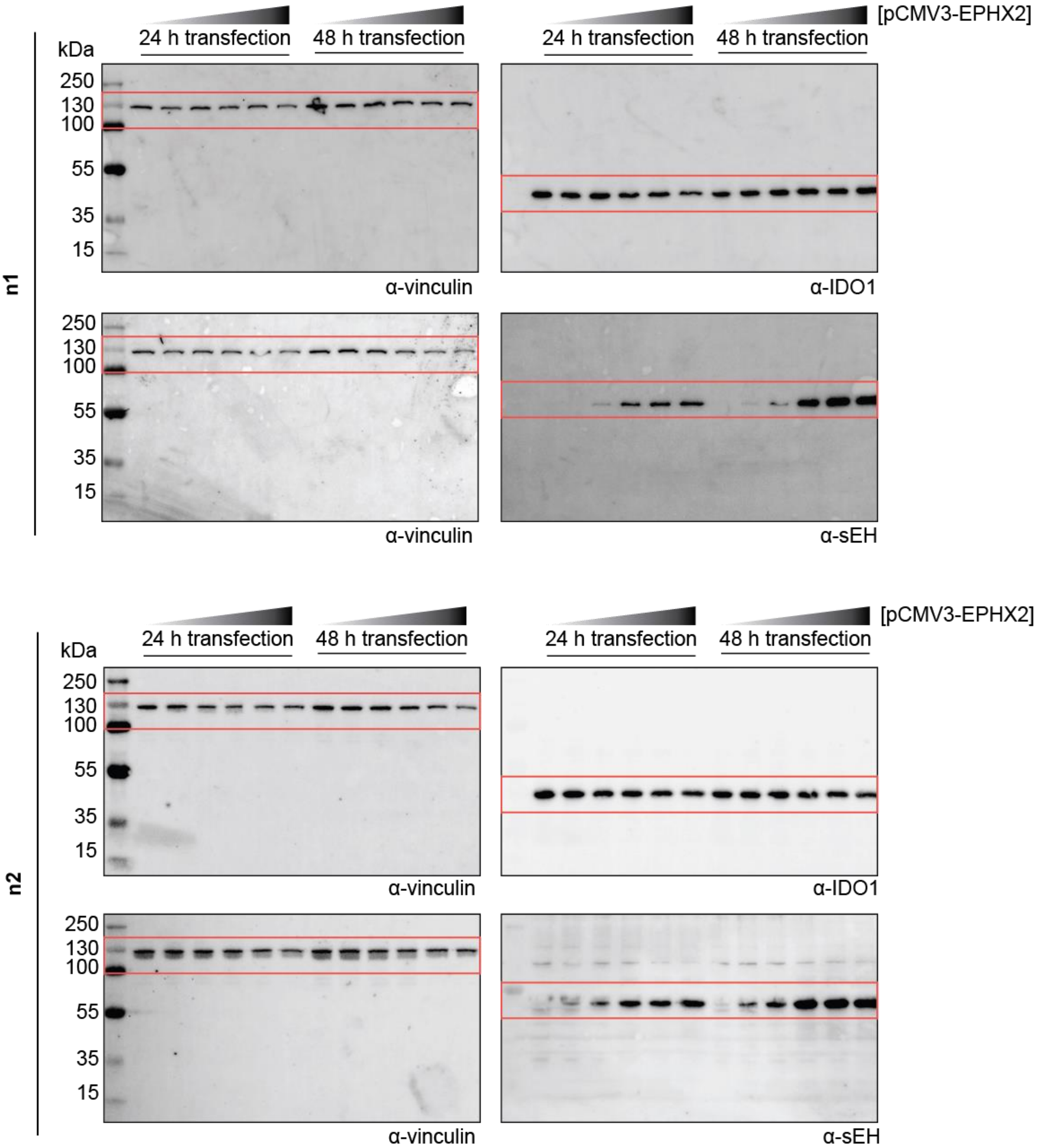

Figure S14. Overexpression of sEH resulted in increased IDO1 levels. HeLa cells were transfected with empty vector or pCMV3-EPHX2 for 24-48 h and treated with IFN-γ for 48 h prior to quantification of protein levels *via* immunoblotting. IDO1 and sEH protein bands were acquired in the IRDye800CW channel, the vinculin control and marker bands were acquired in the IRDye680RD channel. Related to figure 4B.

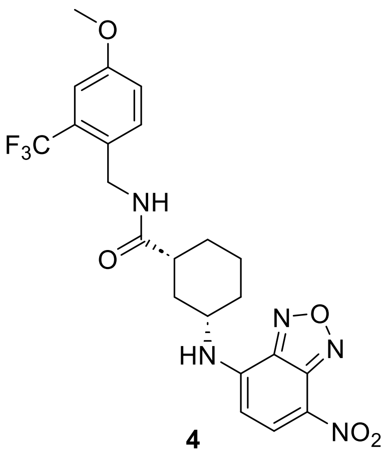

4

Figure S15: Structure of fluorescent tracer **4**[2] used for the sEH-H nanoBRET.

2. Chemical Synthesis

2.1 NMR Spectra

tert-butyl (15-(1-(2-(cycloheptylamino)-2-oxoethyl)-3-(2,2,2-trifluoroacetyl)-1*H*-indol-5-yl)-3,6,9,12-tetraoxa-pentadec-14-yn-1-yl)carbamate (**2a**)

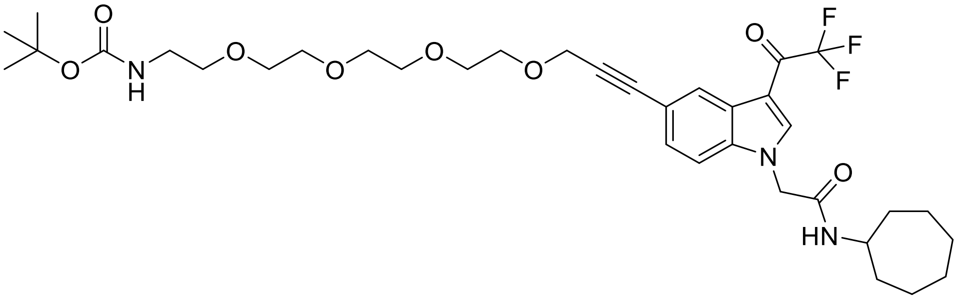

^{1}H NMR

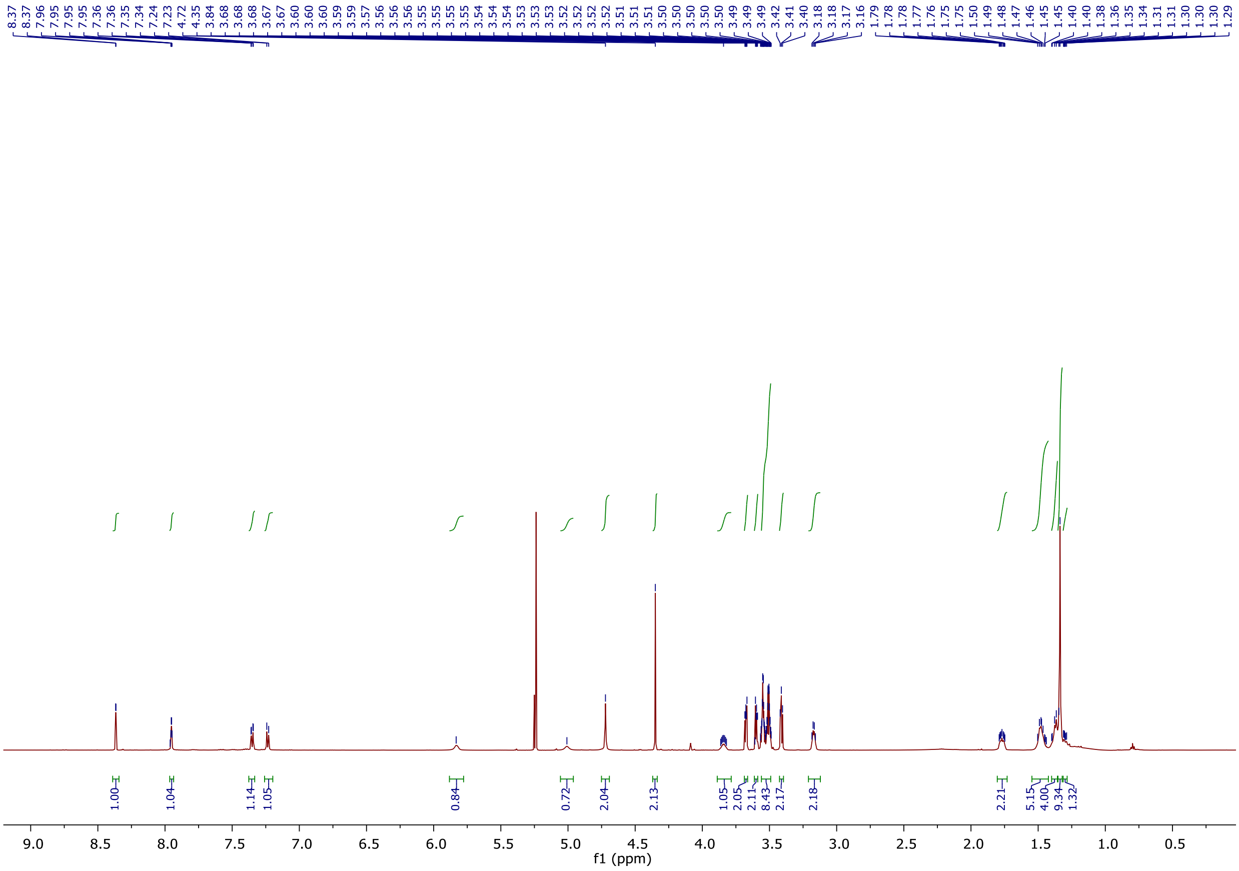

^{13}C NMR

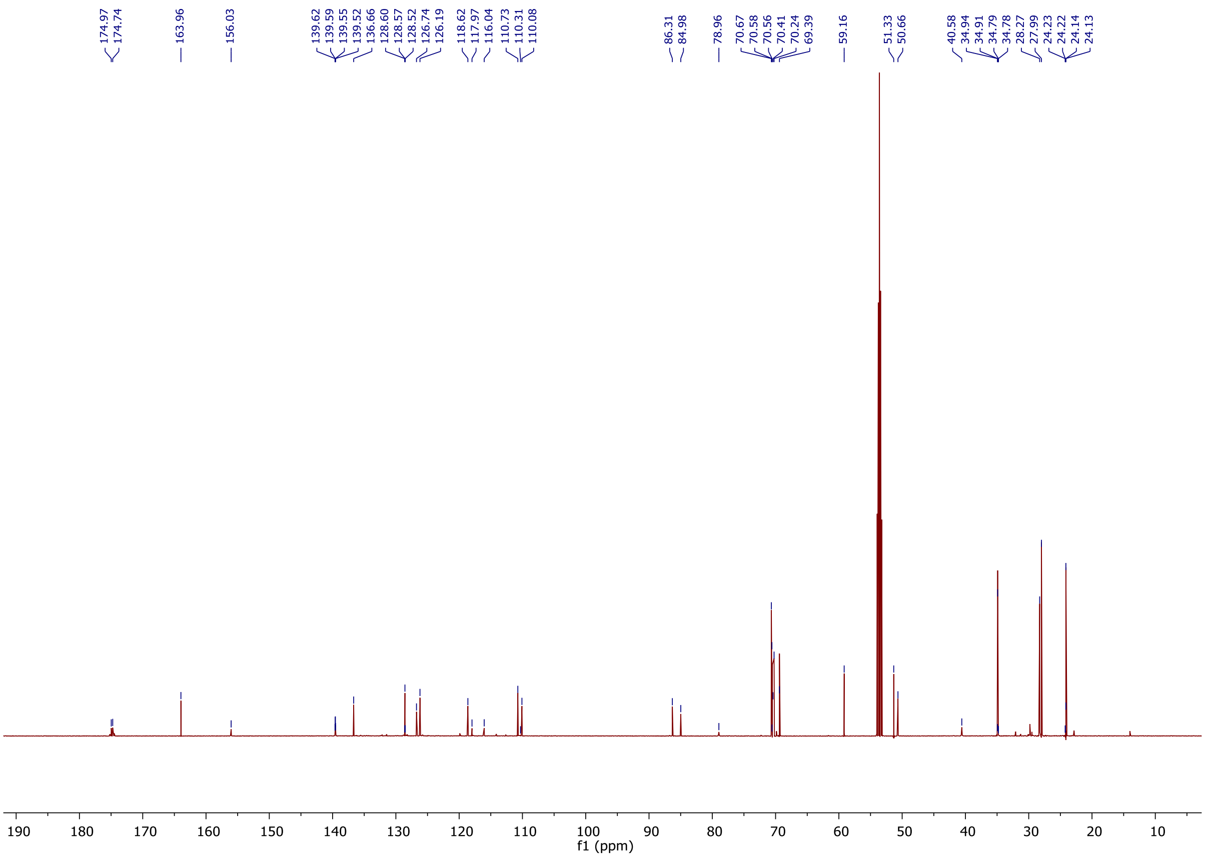

^{19}F NMR

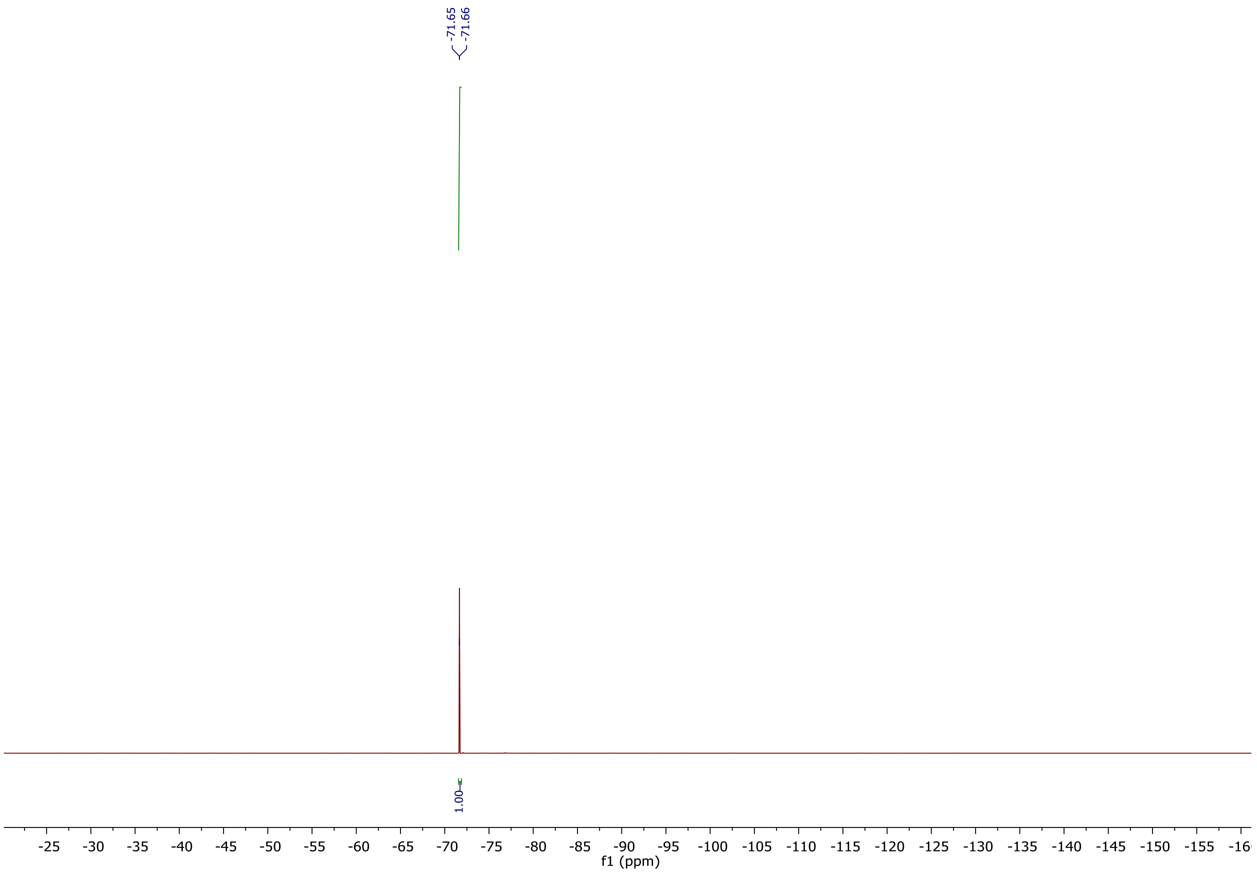

tert-butyl (15-(1-benzyl-3-(2,2,2-trifluoroacetyl)-1*H*-indol-5-yl)-3,6,9,12-tetraoxapentadec-14-yn-1-yl)carbamate (**3a**)

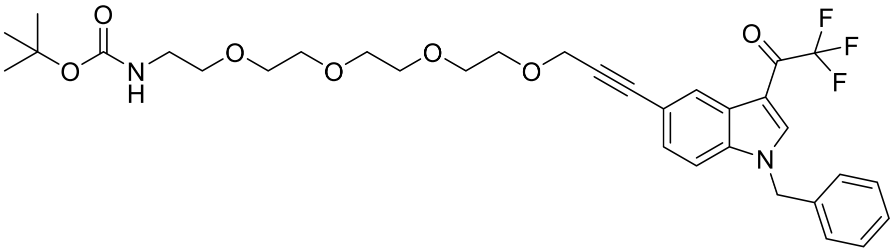

^{1}H NMR

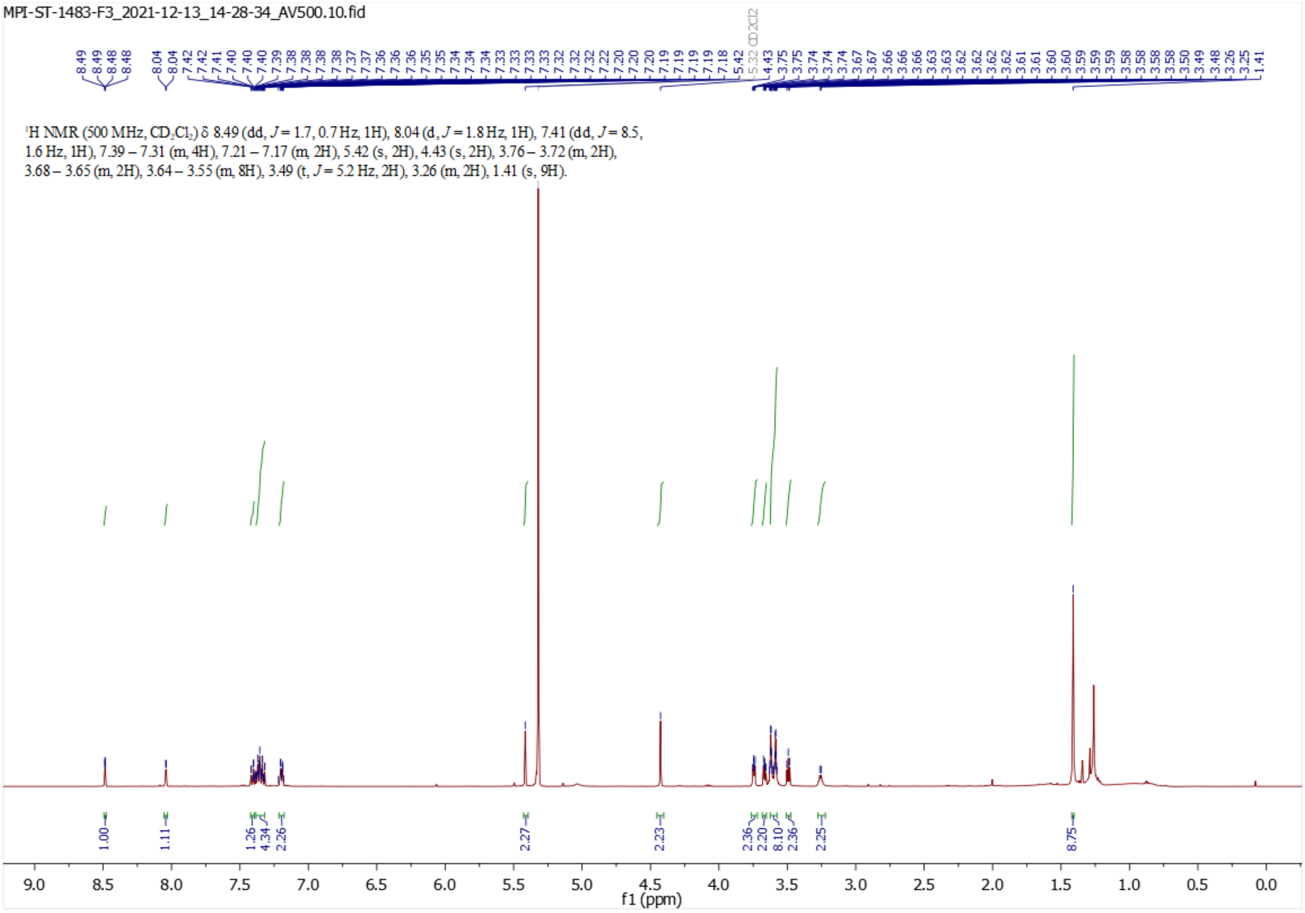

^{13}C NMR

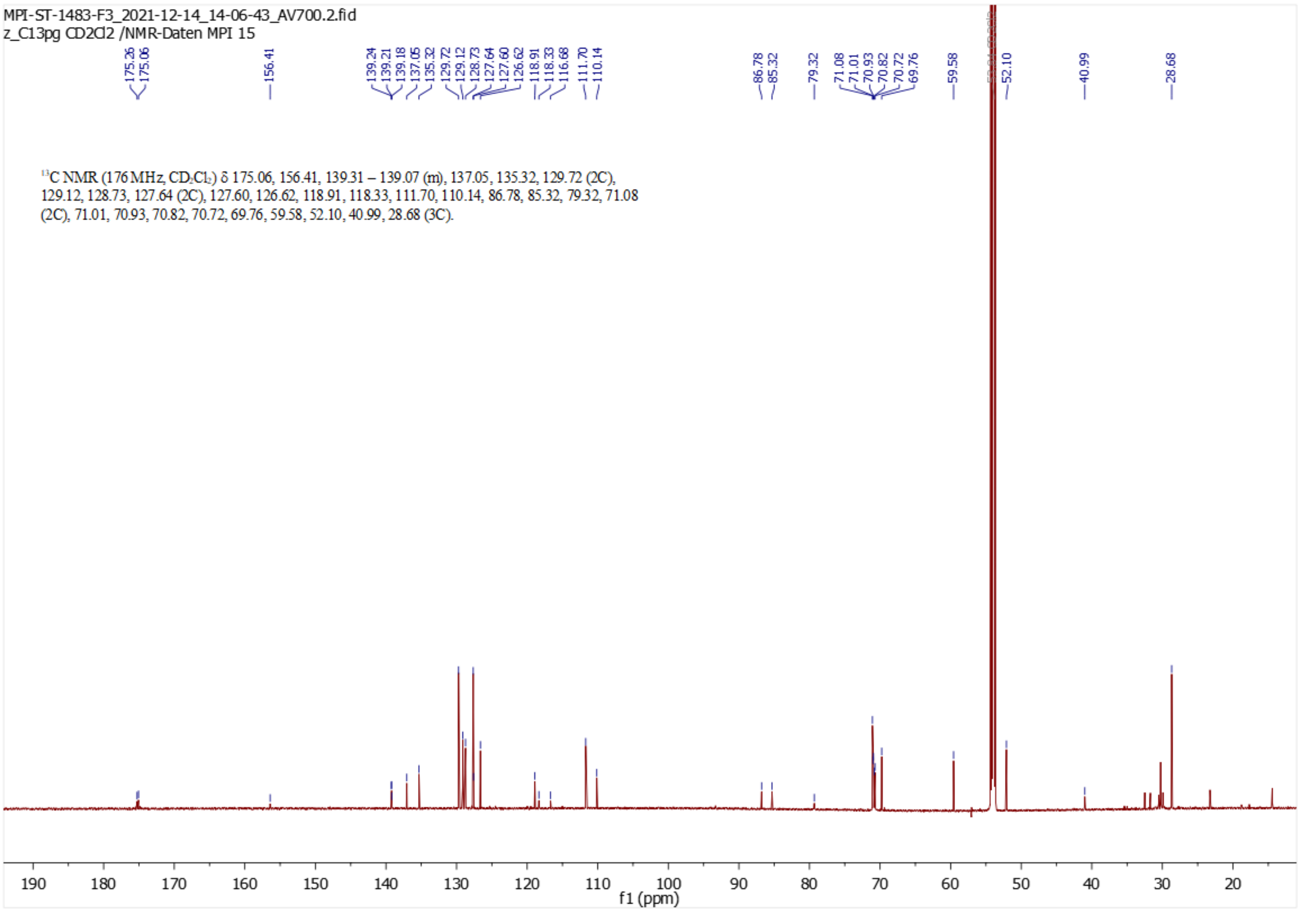
MPI-ST-1483-F3_2021-12-14_14-06-43_AV700.2.fid
z_C13pg CD2Cl2 /NMR-Daten MPI 15
175.26
175.06
156.41
139.24
139.21
139.18
137.05
135.32
129.72
129.12
128.73
127.64
127.60
126.62
118.91
118.33
116.68
111.70
110.14
86.78
85.32
79.32
71.08
71.01
70.93
70.82
70.72
69.76
59.58
52.10
40.99
28.68
13C NMR (176 MHz, CD2Cl2) δ 175.06, 156.41, 139.31 – 139.07 (m), 137.05, 135.32, 129.72 (2C), 129.12, 128.73, 127.64 (2C), 127.60, 126.62, 118.91, 118.33, 111.70, 110.14, 86.78, 85.32, 79.32, 71.08 (2C), 71.01, 70.93, 70.82, 70.72, 69.76, 59.58, 52.10, 40.99, 28.68 (3C).
190
180
170
160
150
140
130
120
110
100
90
80
70
60
50
40
30
20
f1 (ppm)

^{19}F NMR

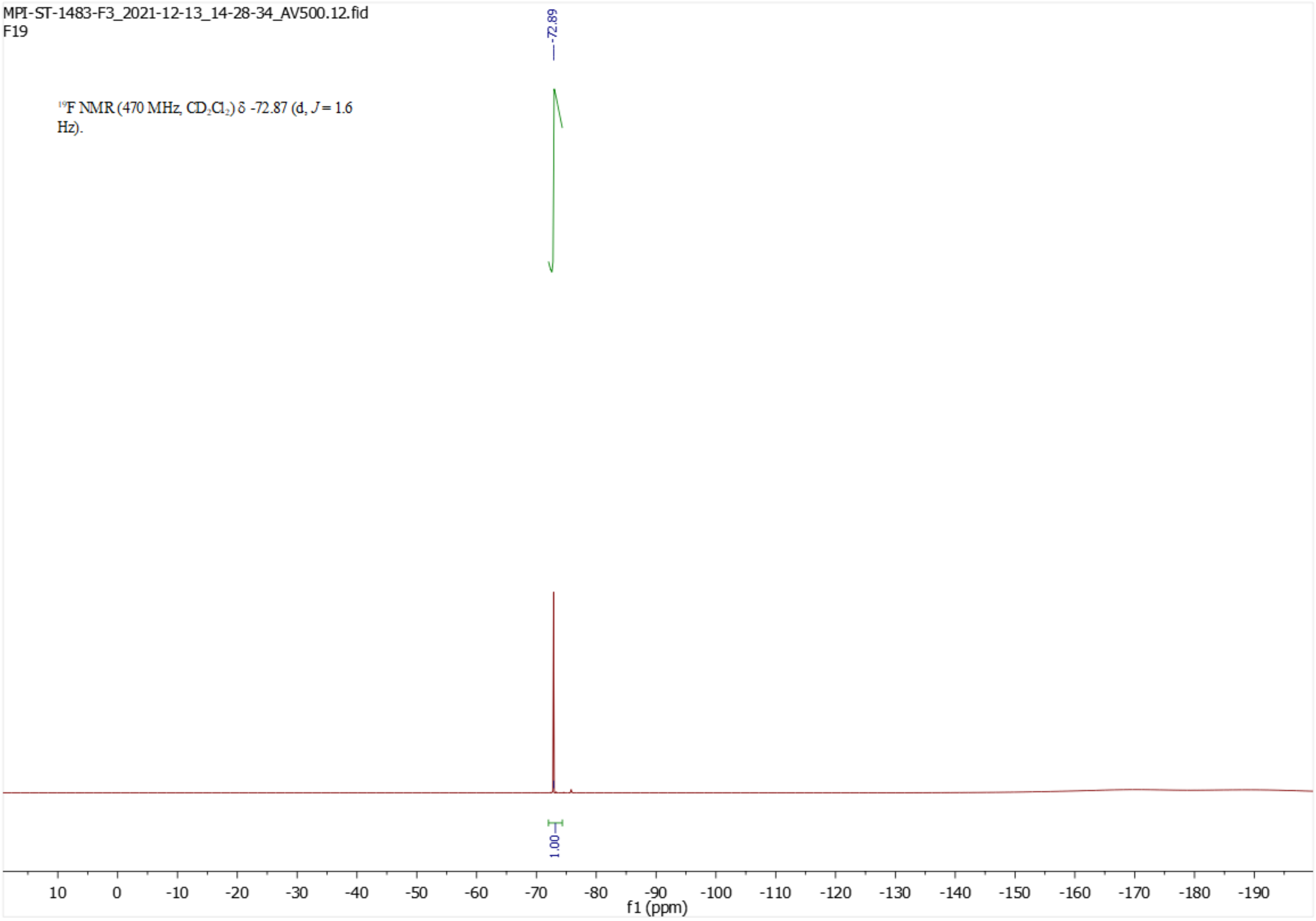

2.2 HPLC Spectra

2-(5-(3-(2-(2-aminoethoxy)ethoxy)prop-1-yn-1-yl)-3-(2,2,2-trifluoroacetyl)-1*H*-indol-1-yl)-N-cycloheptyl-acetamide (**2b**)

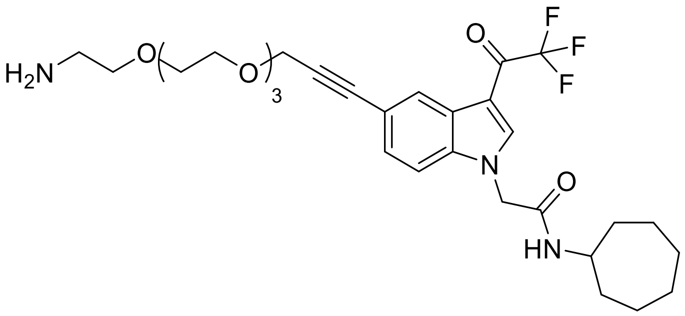

Brown solid, 98.12% yield. **HRMS**: calculated for $[M+H]^+$, $C_{30}H_{41}F_3N_3O_6^+$ = 696.29, found: 696.26; ; calculated for $[2M+H]^+$, $C_{60}H_{81}F_6N_6O_{12}^+$ = 1191.58, found: 1090.52.

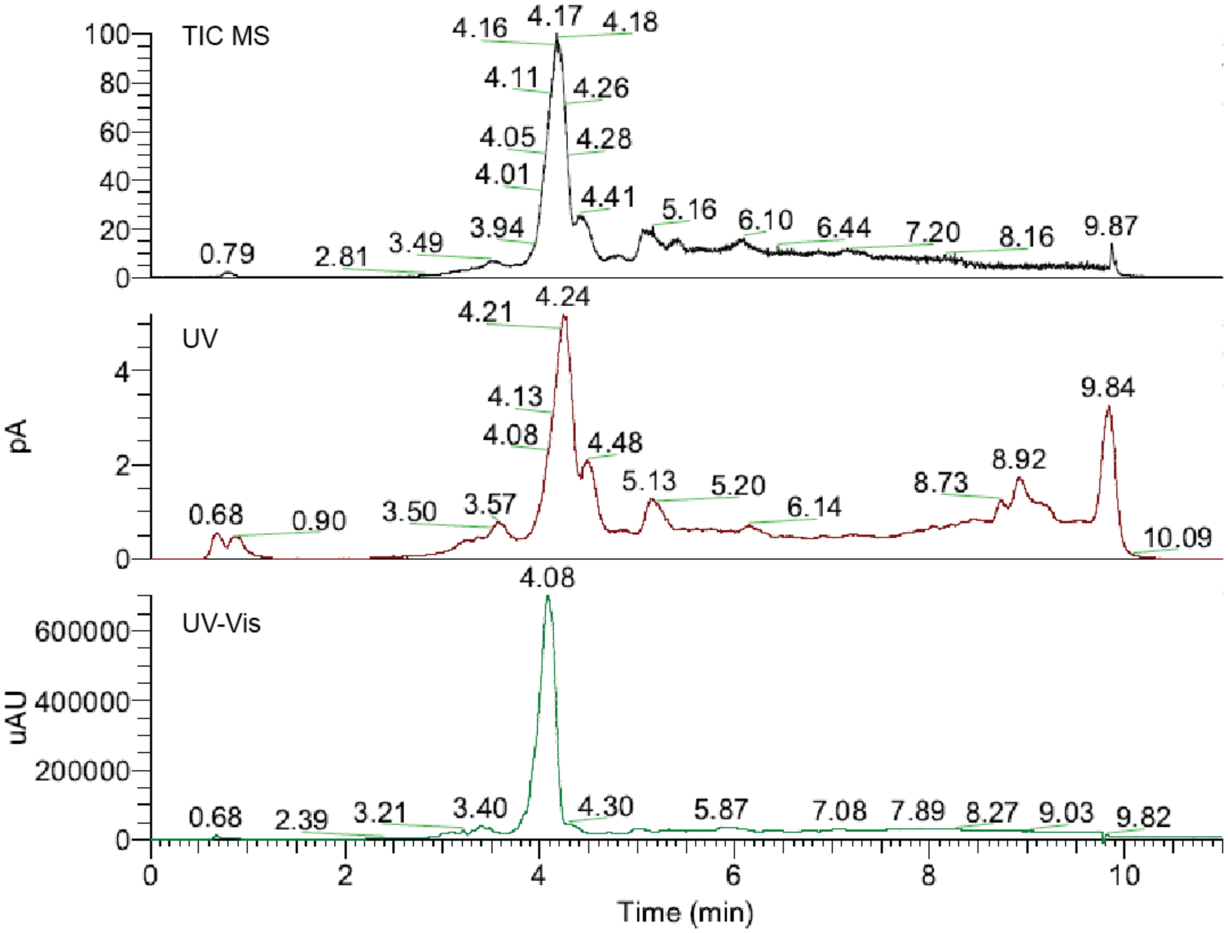

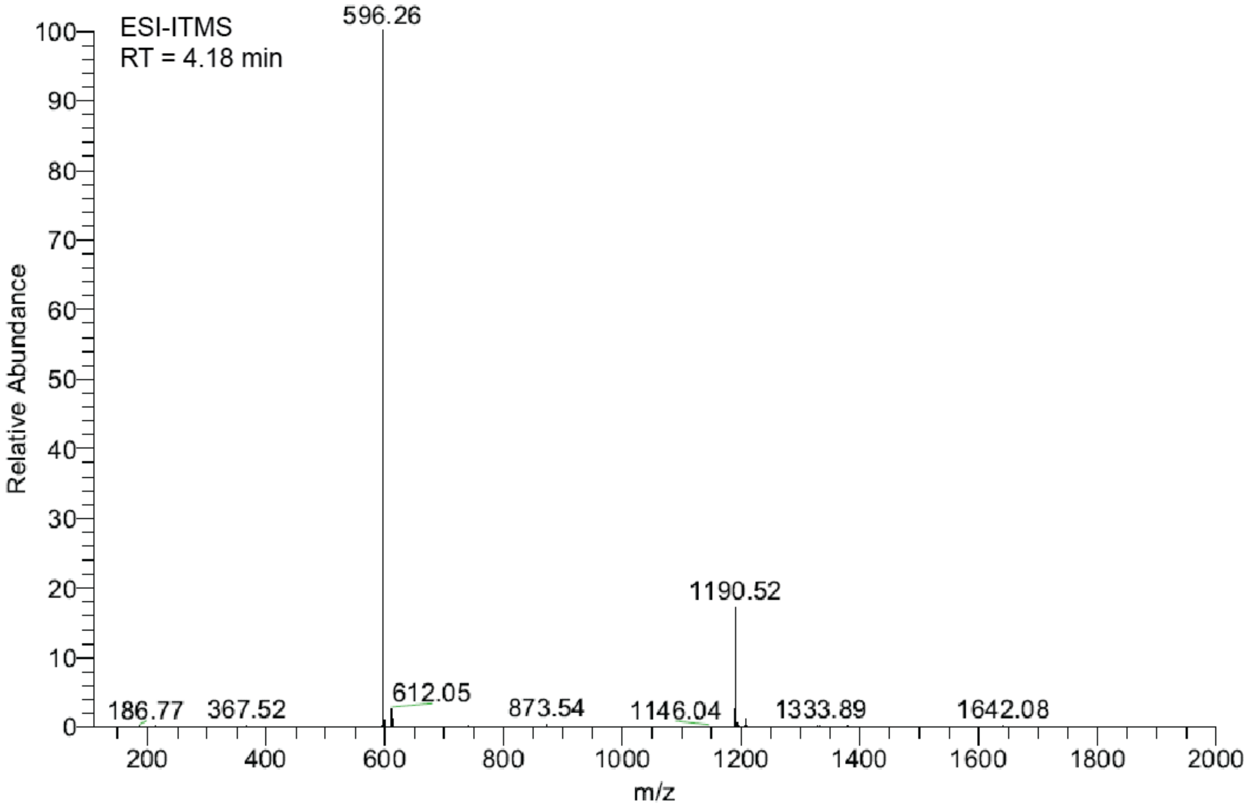

1-(5-(3-(2-(2-aminoethoxy)ethoxy)prop-1-yn-1-yl)-1-benzyl-1*H*-indol-3-yl)-2,2,2-trifluoroethan-1-one (**3b**)

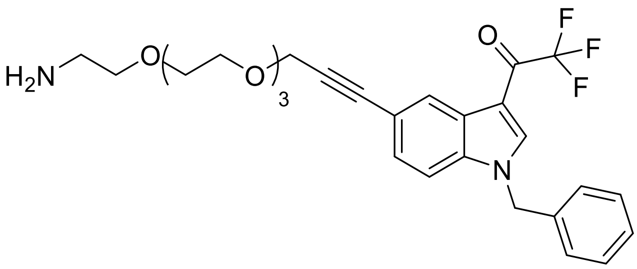

Brown solid, 99.79% yield. **HRMS**: calculated for [M+H]$^+$, $C_{28}H_{32}F_3N_2O_5^+$ = 533.23, found: 533.20; calculated for [2M+H]$^+$, $C_{56}H_{63}F_6N_4O_{10}^+$ = 1065.44, found: 1064.42.

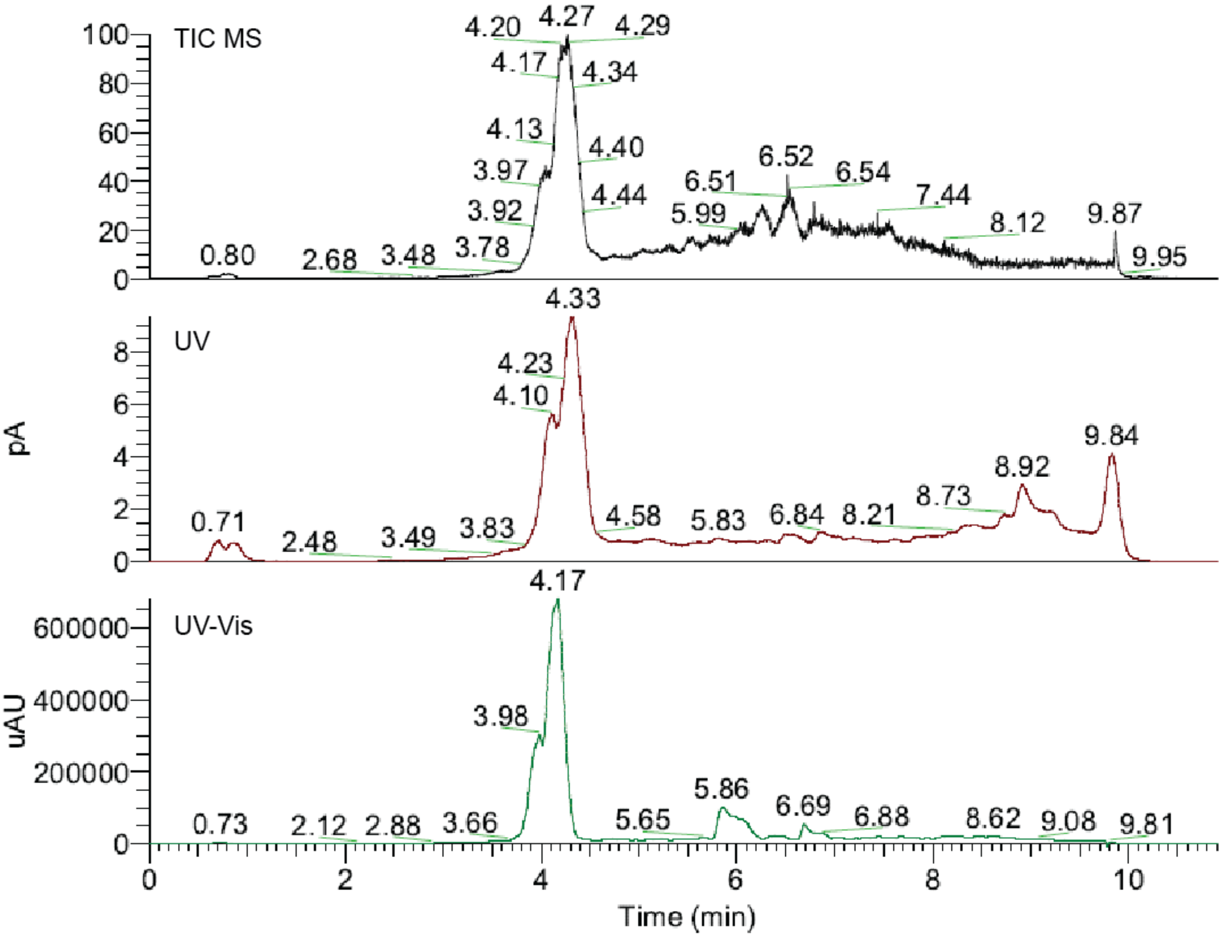

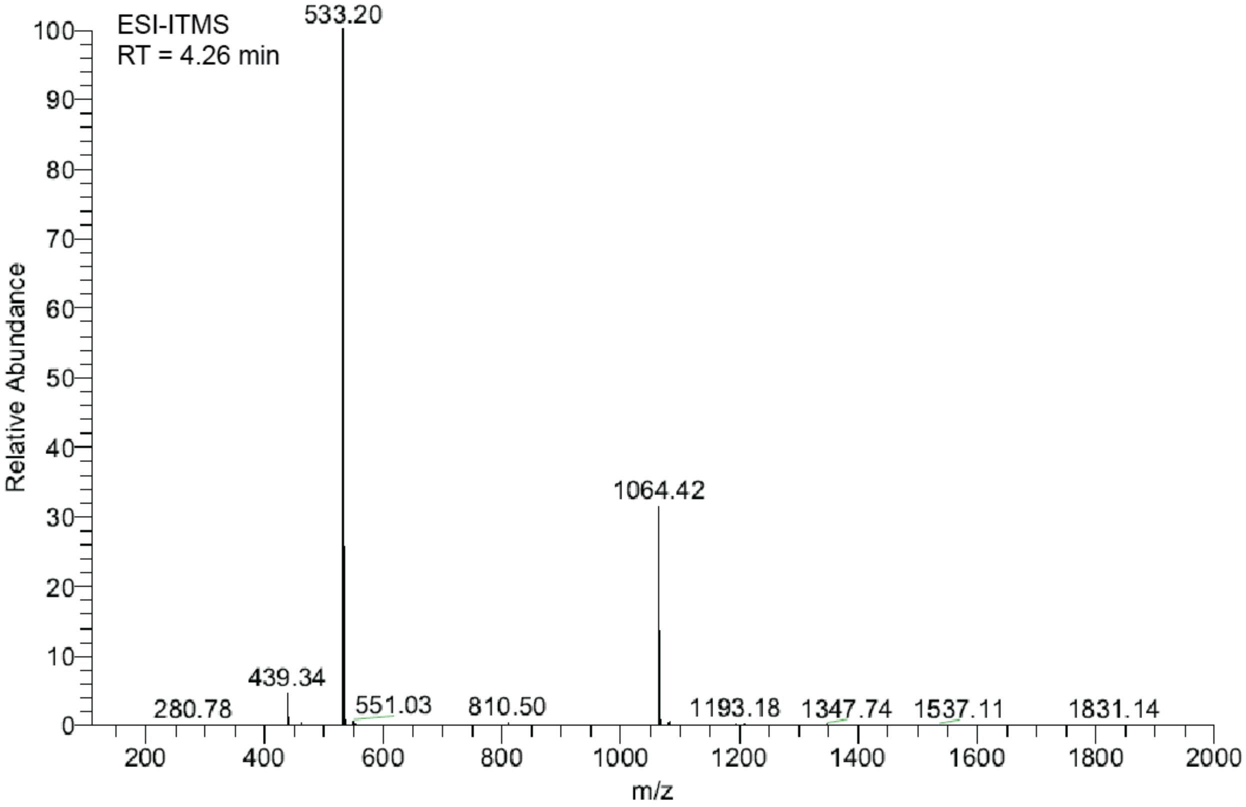
ESI-ITMS
RT = 4.26 min
533.20
1064.42
439.34
280.78
551.03
810.50
1193.18
1347.74
1537.11
1831.14
Relative Abundance
m/z

3. Supplementary References

1. Goedhart, J.; Luijsterburg, M. S. VolcaNoseR is a web app for creating, exploring, labeling and sharing volcano plots. *Sci Rep* **2020**, *10* (1), 20560. DOI: 10.1038/s41598-020-76603-3.

2. Brunst, S.; Schönfeld, J.; Breunig, P.; Burgers, L. D.; DeMeglio, M.; Ehrler, J. H. M.; Lillich, F. F.; Weizel, L.; Hefendehl, J. K.; Fürst, R.; et al. Designing a Small Fluorescent Inhibitor to Investigate Soluble Epoxide Hydrolase Engagement in Living Cells. *ACS Med Chem Lett* **2022**, *13* (7), 1062-1067. DOI: 10.1021/acsmedchemlett.2c00073.
